# Moderately Hydrophobic Polymer Protective Layer Enables Zn (100) Deposition for High Utilization Zinc Anodes

**DOI:** 10.1007/s40820-026-02208-6

**Published:** 2026-05-26

**Authors:** Xiaoming Fan, Peiyi Wu, Yucong Jiao

**Affiliations:** State Key Laboratory of Advanced Fiber Materials, College of Chemistry and Chemical Engineering, Donghua University, Shanghai, 201620 People’s Republic of China

**Keywords:** Polymer protective layer, Moderate hydrophobicity, Zn (100) plane, High Zn utilization, Aqueous Zn batteries

## Abstract

**Highlights:**

- The moderately hydrophobic property of polymer protective layer (PDMAG) prevents the "Grotthuss" effect on Zn surface to inhibit hydrogen evolution reaction.
- The PDMAG protective layer guides Zn^2+^ deposition along (100) plane for rapid stripping/plating.
- PDMAG enables efficient Zn utilization under high DOD_Weight_ for aqueous zinc ion batteries.

**Abstract:**

Evolving Zn anodes with high areal capacity at elevated depths of discharge (DOD) is crucial for scalable aqueous battery, yet plagued by dendritic growth and parasitic reactions. Here, we constructed a polymer protective layer (PDMAG@Zn) comprising hydrophobic poly(N,N-dimethylacrylamide) (PDMAA) and zincophilic cationic guar gum (CGG) via an in situ strategy. The moderately hydrophobic property of PDMAA prevents the "Grotthuss" effect on Zn surface to inhibit parasitic reactions while synergistically accelerates Zn^2+^ desolvation behavior with a molecular lubrication mechanism. Additionally, the PDMAG layer guides Zn^2+^ deposition along (100) plane for rapid stripping/plating performance. Coupled with CGG-induced interfacial stabilization, the symmetrical batteries achieve ultralong cycling of 6580 h at 1 mA cm^−2^, 1 mAh cm^−2^, and 300 h at 1 mA cm^−2^, 15 mAh cm^−2^ with 91.5% DOD_Weight_. Furthermore, the Zn||V_2_O_5_ full battery delivers a high capacity of 335 mAh g^−1^ at 1 A g^−1^, underscoring superior feasibility in practical applications.

**Figure:**
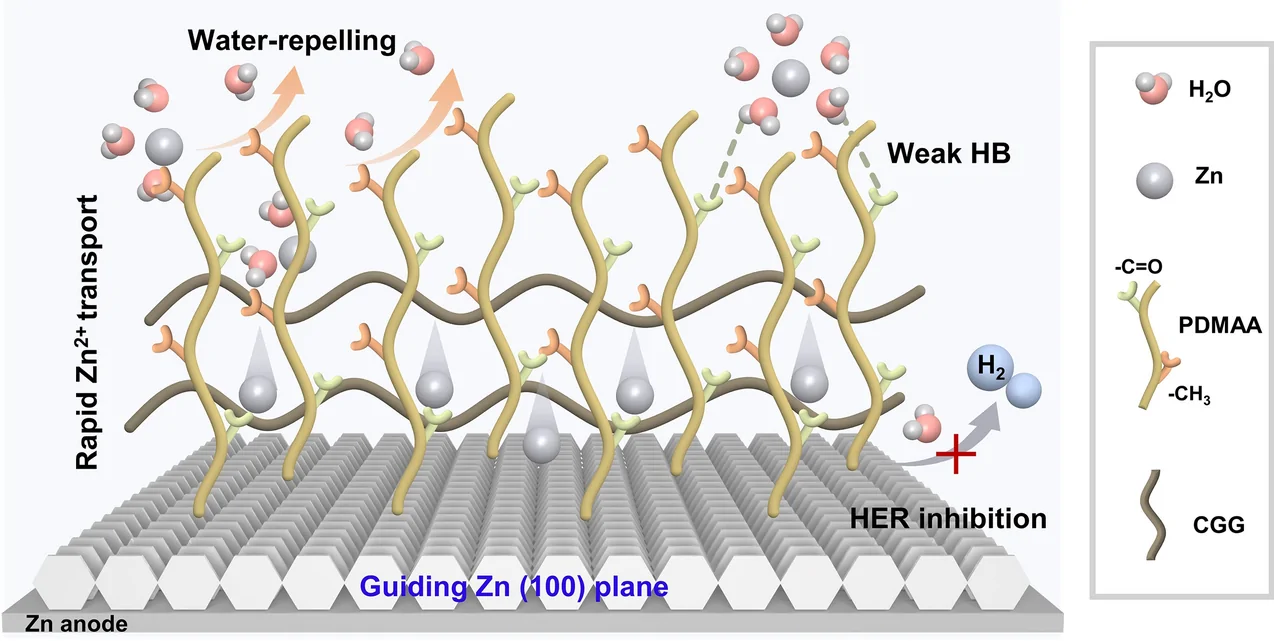

**Supplementary Information:**

The online version contains supplementary material available at 10.1007/s40820-026-02208-6.

## Introduction

Rechargeable aqueous zinc ion batteries (AZIBs) are attractive for large-scale energy storage systems owning to its natural abundance, intrinsic safety, facile manufacturability, and high theoretical specific capacity (820 mAh g^−1^) [1–3]. Unfortunately, Zn inherently exhibits hydrogen evolution reaction (HER) catalytic activity in aqueous electrolytes, leading to gas evolution and uncontrollable dendrite growth. These issues compromise the reversibility of Zn electrodes [4, 5]. Conventional AZIBs typically employ excessive Zn to compensate for irreversible losses during the stripping/plating process, which accelerates rapid electrolyte depletion, lowers Zn utilization, and causes pronounced degradation in energy density. These drawbacks often limit battery performance, especially at high current densities and deep depths of discharge (DOD), thereby severely restricting their practical applications. Therefore, constructing dendrite free and anti-corrosion Zn electrodes with high reversibility is urgently essential for long-term stability.

**Fig. 1:**
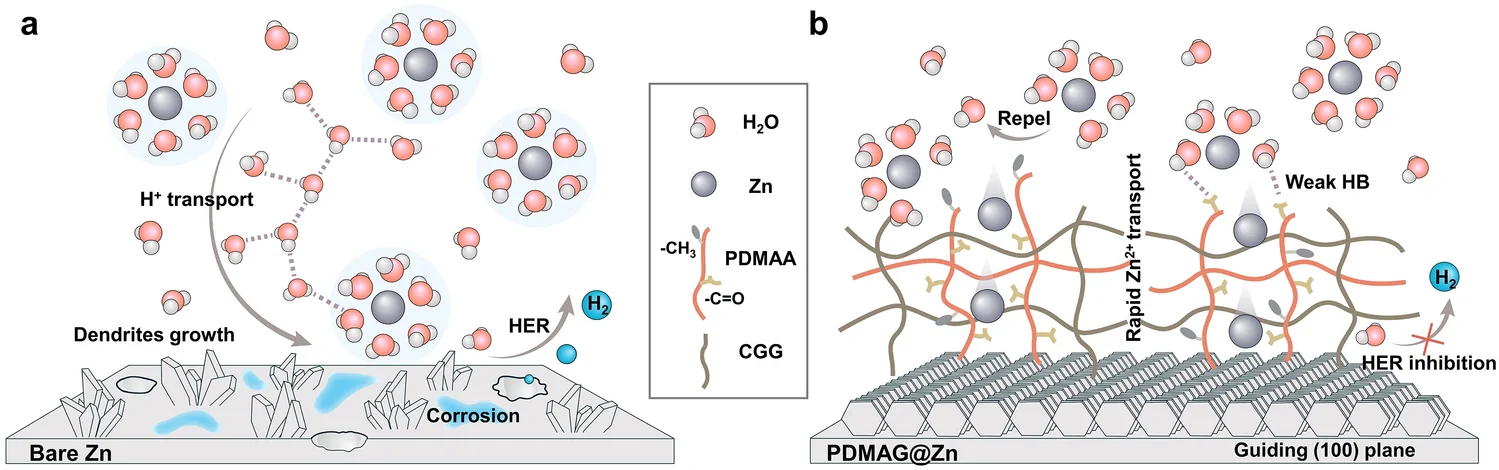
Schematic illustrations and interfacial molecule engineering of **a** bare Zn and **b** PDMAG@Zn

Optimizing a viable protective layer on Zn electrodes to mitigate parasitic reactions by blocking direct contact with H_2_O offers notable advantages for enhancing anode performance [6–8]. Particularly, polymer protective layers (e.g., polydopamine (PDA) [9], polyamide (PA) [10] and polyacrylamide (PAM) [11]) afford mechanical adaptability and polar functional groups, which enable good HER suppression and Zn crystallographic orientation regulation. These advantages reasonably inhibit dendrite growth and improve the reversibility of Zn^2+^ stripping/plating. Unfortunately, although the polymer protective layers with abundant hydrophilic groups restrict H_2_O activity through the formation of strong hydrogen bond (HB) interaction [12], they result in a weak barrier against free water molecules, which partially restrict water mobility, excessive hydrophilicity inevitably leads to interfacial H_2_O enrichment, accelerating severe HER of Zn electrode [13]. The resulting HB networks promote H^+^ transport to the Zn electrode via the "Grotthuss" mechanism, similar to that in aqueous electrolytes (Fig. 1a), thereby limiting both HER suppression and Zn utilization [14]. Recently, incorporating hydrophobic moieties in polymers at the electrode interface has been demonstrated to be effective on blocking the contact between interfacial H_2_O and the Zn anode for facilitated side reaction inhibition [15]. However, excessive hydrophobicity of protective layer with poor electrode wettability hinders Zn^2+^ transport kinetics and imposes a high energy barrier for Zn^2+^ desolvation, resulting in increased charge-transfer resistance (R_ct_) and poor Zn^2+^ stripping/plating reversibility [16]. Therefore, designing polymeric protective layers with relative hydrophobicity is crucial for realizing highly reversible Zn anodes with improved utilization efficiency.

Guiding Zn growth along the (002) plane with polymers is favorable in inhibiting dendrite formation and side reactions [17]. However, its intrinsically lower surface activity and slower crystal growth rate unignorably hinder the efficient Zn^2+^ plating/stripping, thereby compromising AZIB reversibility at high areal capacities [18, 19]. Recent studies have indicated that the Zn (100) plane with intrinsically low surface diffusion barrier preferentially forms vertically aligned, thick, and highly ordered facets with minimized aspect ratio. Moreover, the vertically aligned Zn (100) planes with faster growth kinetics favor-oriented deposition suppress the internal short circuits and deliver the highest critical areal capacity [20–23], indicating that is suitable for achieving fast Zn^2+^ stripping/plating process and ensuring optimal cycling reversibility with high areal capacity and deep DOD conditions. Meanwhile, Zn electrodes with (100) orientation possess the lowest electrochemically active surface area (ECSA) and limited electrolyte accessibility, which helps inhibit side reactions [24–26]. Therefore, harnessing polymer protective layers to modulate the Zn (100) plane could be promising for enhancing Zn utilization and achieving high performance in Zn batteries.

Here, poly(N,N-dimethylacrylamide) (PDMAA) is integrated with cationic guar gum (CGG) to construct a multifunctional polymer protective layer (PDMAG) with balanced hydrophobicity and zincophilicity (Fig. 1b). Compared to PAM, the steric hindrance of the methyl groups in PDMAA endows the PDMAG with moderate hydrophobicity. This property blocks the continuous HB network of H_2_O in the bulk electrolyte and confines H_2_O activity through weak HB interactions between the –C=O groups and H_2_O. Consequently, side reactions are effectively suppressed, and Zn^2+^ desolvation behavior is promoted via a molecular lubrication mechanism. Moreover, PDMAG preferentially adsorbs on the Zn (100) plane to facilitate Zn^2+^ deposition along the (100) plane for fast stripping/plating process, ultimately enabling efficient Zn utilization under high DOD_Weight_. As a result, the PDMAG endows the asymmetrical Zn||Cu batteries with cycling stability for more than 320 h at 5 mA cm^−2^ and 5 mAh cm^−2^ (30.5% DOD_Weight_), and the Zn||V_2_O_5_ pouch battery with a high areal capacity of 28.9 mAh at 0.5 A g^−1^. This work demonstrates a practical strategy for constructing stable and efficient Zn anodes toward advanced aqueous energy storage systems.

## Experimental Section

### Materials

N, N-dimethylacrylamide (DMAA) was purchased from TCL Chemical, and cation guar gum (CGG, viscosity > 2000 mPas) was obtained from Yuanye. Acrylamide (AM) and citric acid (C_6_H_8_O_7_N, 99.5%) were purchased from Sigma-Aldrich, ammonium metavanadate (NH_4_VO_3_, 99%) was supplied by Aladdin. N, N-methylene bis(acrylamide) (MBAA, 99%), potassium persulfate (KPS), and Zn(OTf)_2_ were provided by Adamas Reagent. Zinc foil (thickness: 30 μm) was supplied by Hebei Qingyuan Co., Ltd. All chemicals were used without any further purification.

### Preparation for PDMAG@Zn

The PDMAG polymer protective layer was fabricated via the following procedure. Generally, DMAA monomer (36 wt% relative to deionized water) and CGG were dissolved in deionized water, with a mass ratio of CGG to DMAA is 1:3. After stirring for 5 h, 0.025 wt% MBAA (vs. monomer) and 0.5 wt% KPS (vs. monomer) were added to the above mixture solution as the crosslinking agent and initiator, followed by 20 min of ultrasonic degassing to remove bubbles. The as-prepared precursor solution was then uniformly blade-coated on Zn foil surface and polymerized with ultraviolet curing at the power of 40 W for 40 min to obtain PDMAG@Zn. For comparison, PACG@Zn was prepared using an identical procedure, replacing DMAA with AM as the primary monomer.

### Preparation for V_2_O_5_ Cathode

The V_2_O_5_ cathode was synthesized according to the following method. In a typical process, 20 mL of 1.5 mM aqueous C_6_H_8_O_7_N solution was slowly added to 30 mL of 2 mM NH_4_VO_3_ solution under continuous stirring at 65 °C for 2 h to ensure complete complexation. The resulting solution was then transferred into a Teflon-lined autoclave and subjected to hydrothermal treatment at 170 °C for 12 h. After cooling to room temperature, the precipitate was collected via centrifugation and sequentially washed with deionized water and ethanol for five times. The final product was dried in a vacuum oven at 60 °C for 10 h to obtain the V_2_O_5_ powder. The V_2_O_5_ cathode was obtained by mixing the V_2_O_5_ powder, acetylene black, and PVDF with a weight ratio of 7:2:1 in NMP solvent and then homogeneously casting onto carbon paper with an average mass loading of approximately 1.2 mg cm^−2^.

### Preparation of I_2_ Cathode

The I_2_ cathode was fabricated by homogeneously mixing activated carbon (AC), Ketjen black (KB), and I_2_ as a powder mixture. Subsequently, PTFE slurry and isopropanol were gradually added. The as-prepared mixture was subjected to iterative roll-pressing and then laminated onto Ti mesh. After vacuum drying at 45 °C for 12 h, the I_2_ cathode was finally obtained.

### Materials Characterization

Material structure and molecular interactions were analyzed by Fourier transform infrared spectroscopy (FTIR Nicolet IS50), Raman spectra (Thermo Fisher Scientific DXR2xi) and ^1^H nuclear magnetic resonance (NMR Bruker AV-400). Low-field ^1^H NMR measurements of PACG-Zn^2+^ and PDMAG-Zn^2+^ hydrogels were conducted at room temperature using a VTMR20-010V-I NMR analyzer (Suzhou Niumag Analytical Instrument Corporation, China). During testing, samples were sealed in glass vials and the background signals were subtracted to eliminate interference from ambient moisture and the sample container. The morphologies of Zn anodes after cycling were characterized by scanning electron microscopy (SEM, HitachiSU8230) and Atom Force Microscope (AFM, Dimension Icon). Powder X-ray diffractometer (XRD, Rigaku D/max-2550VB) was employed to confirm the side reaction products on the Zn surface after the cycling process.

### Electrochemical Measurements

The symmetrical/asymmetrical Zn batteries were assembled by 2016-type coin cells with Zn foils (30 μm), Cu foils and Ti foils as electrodes, 2 M Zn(OTf)_2_ and glass fibers were separately used as electrolyte and separators. The cycle performance of symmetrical/asymmetrical Zn batteries and Zn||V_2_O_5_ batteries were conducted on the Neware battery test system. The cyclic voltammetry (CV), Linear sweep voltammetry (LSV), electrochemical impedance spectroscopy (EIS) and Chronoamperometry (CA) tests were tested on the Autolab 204 workstation. Gas chromatography (GC) for H_2_ generation was detected by GC-2060 coupled with an electrochemical workstation. The LSV tests were conducted with a three-electrodes system, which Zn metal (1 cm × 1 cm), Pt plate (1 cm × 1 cm) and Ag/AgCl were employed as working, counter and reference electrodes, respectively.

The Arrhenius activation energy (*E*_*a*_) was calculated according to Eq. (1):

1 $$ \frac{1}{R^{ct}} = A \exp\left( \frac{ - E_{a}}{RT} \right)  $$

The R_ct_, A, T, and R represent the interface resistance, frequency factor, gas constant, and the absolute temperature, respectively.

The transference number of Zn^2+^ (t_Zn_^2+^) was calculated by Eq. (2):

2 $$ t_{Zn^{2 +}} = \frac{I_{\mathrm{s}{(\Delta V - I_{0}R_{0})}}}{I_{0}{{(\Delta V - I_{s}R_{s})}}} $$

where I_0_ and R_0_ are separately represent the initial current and related resistance, I_S_ and R_S_ are the current and the related resistance after polarization, the applied polarization potential (ΔV) is 20 mV.

The electrical double-layer capacitance (C_EDL_) was obtained with Eq. (3):

3 $$ C_{EDL} = \frac{i}{v} $$

where i represents the capacitive current and v is corresponding to the scan rate, C_EDL_ can be obtained from the fitted slope of i versus v curves.

The two typical instantaneous and progressive nucleation were normalized to (I/I_m_)^2^-(t/t_m_) curves and analyzed using the Scharifker-Hills mode [27], which describes as Eqs. (4) and (5):

4 $$ {\text{Instantaneous }}\;{\mathrm{nucleation}}:(I/I_m)^2 = \frac{1.9542}{{(t/t_m)}}\{ 1 - \exp [ - 1.2564(t/t_m)]\} ^2 $$

5 $$ {\mathrm{Progressive}}\;{\text{ nucleation}}:(I/I_m)^2 = \frac{1.2254}{{(t/t_m)}}\{ 1 - \exp [ - 2.3367(t/t_m)]\} ^2 $$

where I_m_ and t_m_ are separately stand for the peak current response at the early stage of nucleation and the correspond reaching time derived from the CA curves.

The theoretical specific capacity of zinc metal is 820 mAh g^−1^, here, we use the weight of Zn to calculate the value of depth of discharge (DOD_Weight_) [28], which the weight of Zn at 1 × 1 cm^2^ size used for testing is about 20 mg, providing a capacity of 20 mg × 820 mAh g^−1^ = 16.4 mAh. Therefore, the DOD_Weight_ was calculated according to Eq. (6):

6 $$ {\mathrm{DOD}}_{{{\mathrm{Weight}}}} (\% ) \, = \, \frac{{{\text{areal }}\;{\text{capacity }}({\text{mA h cm}}^{ - 2} ) \, \times \, 1{\text{ cm}}^{{2}{}} }}{{16.4{\text{ mA h}}}} $$

### Theoretical Computation

The theoretical computation based on the density functional theory (DFT) was performed by using a program of DMol^3^ in the Materials Studio to visualize the interactions between the interface of electrode/electrolyte. The Perdew-Burke-Ernzerhof (PBE) functional combined with generalized gradient approximation (GGA) was used to describe the exchange correlation energy. The core electrons were treated with DFT semi-core pseudopotentials. The binding energies (E_binding_) were calculated through Eq. (7) [29]:

7 Ebinding=EAB-EA-EB

## Results and Discussion

### Interfacial Engineering and Interactions Characterizations

The preparation of the hydrophobic polymer protective layer on the Zn metal surface is illustrated in Fig. S1. As shown in the cross-sectional SEM image (Fig. S2), the PDMAG layer adheres firmly to the Zn surface with a thickness of approximately 3 μm, anchored by the strong zincophilicity of the natural polymer CGG bearing quaternary ammonium groups (-N(CH_3_)_3_^+^). This robust interfacial integration contributes to enhanced electrochemical stability. The hydrophobicity of the artificial layer is critical for mitigating side reactions at the Zn electrode.

**Fig. 2:**
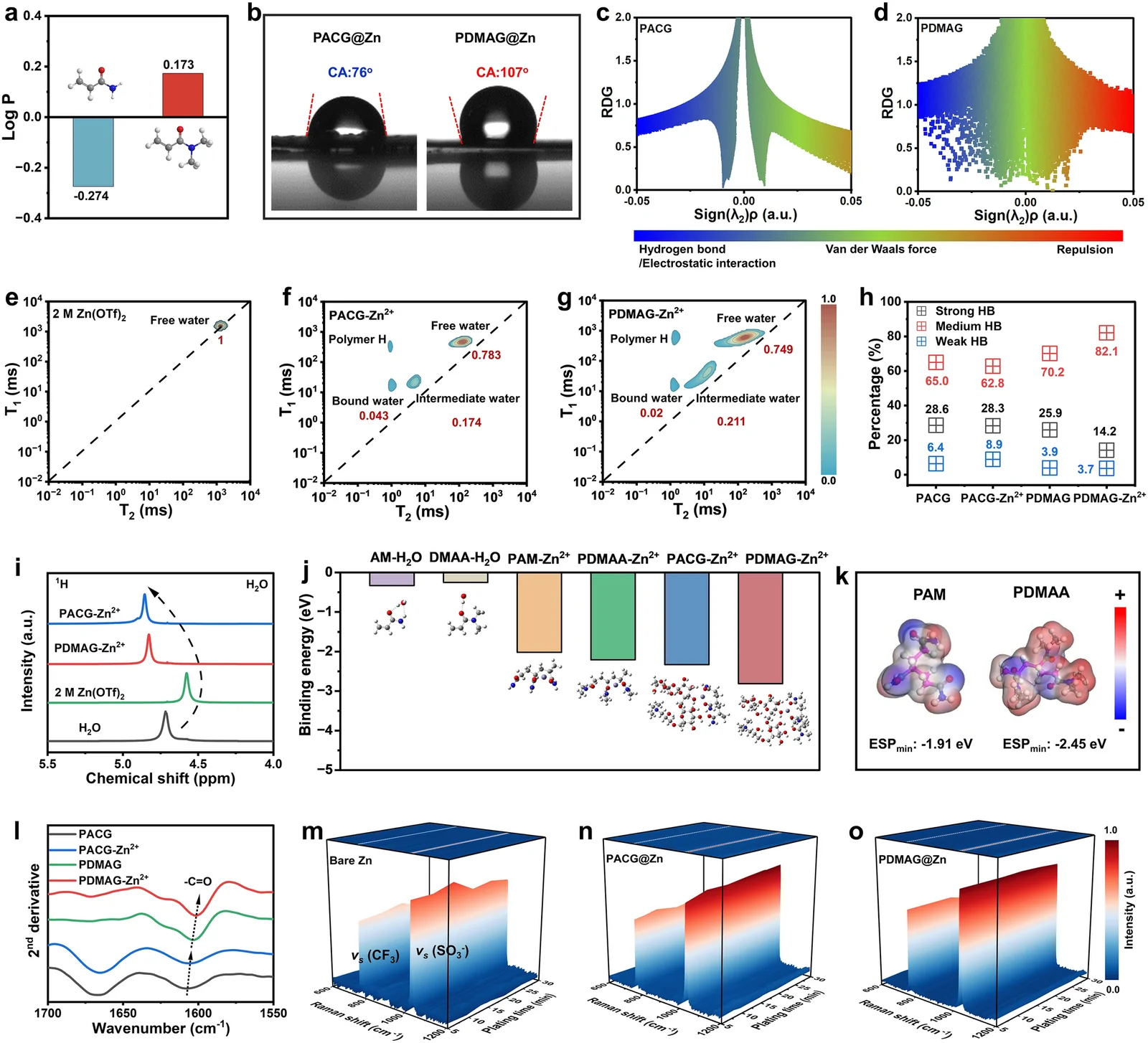
Water activity characterization. **a** *LogP* value comparisons of PAM and PDMAA. **b** The contact angle tests of bare Zn, PACG@Zn, and PDMAG@Zn. RDG scatter plots for **c** PACG, and **d** PDMAG. 2D LF-^1^H *T*_*1*_*-T*_*2*_ NMR spectra of **e** 2 M Zn(OTf)_2_, **f** PACG-Zn^2+^, and **g** PDMAG-Zn^2+^ hydrogels. **h** Percentage of different HB fitted areas calculated from Raman spectra. **i** ^1^H NMR spectra of H_2_O in 2 M Zn(OTf)_2_, PACG-Zn^2+^, and PDMAG-Zn^2+^.** j** Binding energy calculations. **k** The ESP distribution. **l** Second-derivative FTIR curves. In situ Raman spectra at the electrode/electrolyte interface during Zn deposition with **m** bare Zn, **n** PACG@Zn, and **o** PDMAG@Zn

To evaluate this property, the octanol–water partition coefficient (*logP*) was calculated for PAM and PDMAA separately. As revealed in Fig. 2a, PDMAA exhibits a higher *logP* value (0.173) than PAM (-0.274), indicating the enhanced lipophilicity (hydrophobicity) of PDMAA imparted by its dual -CH_3_ substituents. Contact angle tests (Figs. 2b and S3) further demonstrate that the PDMAG@Zn features a higher contact angle value of 107° with the 2 M Zn(OTf)_2_ aqueous electrolyte compared to bare Zn (87°) and PACG@Zn (76°), representing that the two hydrophobic -CH_3_ groups on PDMAA effectively repel H_2_O from the Zn surface for the water-driven corrosion suppression.

To highlight the molecular interactions of PACG and PDMAG, reduced density gradient scatters (RDG) were conducted [30]. As shown in Fig. 2c, d and the corresponding spatial distribution maps in Fig. S4, the scatter for PDMAG exhibits weaker HB and stronger repulsive interactions compared to PACG. Particularly, the observed electrostatic interaction region confirms the formation of an adsorbed layer on the Zn anode surface, which originates from the -N(CH_3_)_3_^+^ of CGG component. Furthermore, the X-ray photoelectron spectra (XPS) in Fig. S5 illustrate that PDMAA@Zn shows a N 1*s* peak at 399.7 eV (–N(CH_3_)_2_), while PDMAG@Zn exhibits a new 402.2 eV peak of –N(CH_3_)_3_^+^, and the Zn 2*p*_3/2_ peak shifts to a higher binding energy. These results further confirms the adsorb interaction of -N(CH_3_)_3_^+^ groups of CGG on the Zn surface. Two dimensional (2D) low-field ^1^H *T*_*1*_*-T*_*2*_ NMR relaxation maps were subsequently implemented to investigate the dynamic behavior of H_2_O molecules. Generally, the *T*_*1*_*/T*_*2*_ ratio is indicative of ^1^H mobility, with lower values presenting higher mobility [31]. As depicted in Fig. 2e–g, the spin population of free water located at the transverse relaxation time (*T*_*2*_) of 200–3000 ms in 2 M Zn(OTf)_2_ exhibits a *T*_*1*_*/T*_*2*_ ratio of 1.2, whereas PACG-Zn^2+^ and PDMAG-Zn^2+^ display higher ratios of 3.9 and 3.5. The increased* T*_*1*_/*T*_*2*_ ratio in PACG-Zn^2+^ and PDMAG-Zn^2+^ hydrogels indicate strong active H_2_O restriction in polymers. However, the decreased *T*_*1*_/*T*_*2*_ ratio in PDMAG-Zn^2+^ hydrogel indicates the enhanced higher polymer chains mobility compared to PACG-Zn^2+^ hydrogel, which facilitates Zn^2+^ transport and enables excellent rate capability. Moreover, the PACG-Zn^2+^ and PDMAG-Zn^2+^ hydrogels display three spin populations in the *T*_*2*_ ranges of 0.1–3 and 3–20 ms, corresponding to bound water, polymer H, and intermediate water. After normalization to hydrogen content, the proportion of free water in PDMAG-Zn^2+^ (0.749) is lower than in PACG-Zn^2+^ (0.783). In contrast, the fraction of intermediate water in PDMAG-Zn^2+^ (0.211) is higher than in PACG-Zn^2+^ (0.174). These findings suggest that the PDMAG-Zn^2+^ polymer preferentially forms weaker-bound hydration structures with H_2_O, in contrast to the more strongly confined bound water dominating in PACG-Zn^2+^. The presence of weakly associated intermediate water, coupled with the inherent mobility of the polymer chains, may act as a molecular lubrication channel to facilitate Zn^2+^ migration along the polymer chains and enable fast regulation of Zn deposition. The tested ionic conductivity of separator with PDMAG is higher than that with PACG (Fig. S6), further implying that the mobility of the PDMAG polymer chains facilitates the fast Zn^2+^ transport. Additionally, to further substantiate the enhanced chain mobility of the PDMAG polymer, differential scanning calorimetry (DSC) measurements were performed. As presented in Fig. S7, PDMAG exhibits a lower glass transition temperature (T_g_) of −27.3 °C compare with PACG (−14.5 °C), indicating higher mobility of polymer chain.

Raman spectra were performed to probe the HB disruptions on H_2_O molecules within polymer protective layer at electrodes/electrolyte interface. The –OH stretching band in the range between 3800 and 3000 cm^−1^ was deconvoluted into three characteristic peaks corresponding to strong HB, medium HB, and weak HB for water, located at approximately 3230, 3425, and 3600 cm^−1^, respectively (Fig. S8). The HB states of H_2_O within different polymer matrices were further quantified by calculating the corresponding peak areas [32]. As presented in Fig. 2h, the proportion of medium HB in both PDMAG and PDMAG- Zn^2+^ is higher than that in PACG and PACG-Zn^2+^. Meanwhile, the content of bound water related weak HB with polymer is substantially lower in the PDMAG-based systems. These observations are consistent with the results obtained from LF ^1^H *T*_*1*_*-T*_*2*_ NMR analysis. Additionally, the ^1^H NMR spectra (Fig. 2i) display that, compared with the pure Zn(OTf)_2_ electrolyte, the ^1^H NMR chemical shift of H_2_O in PDMAG-Zn^2+^ and PACG-Zn^2+^ progressively moves downfield. This shift indicates strong intermolecular HB interactions between H_2_O and PACG, whereas PDMAG tends to form relatively weaker HB interactions that confine interfacial H_2_O.

To gain molecular-level insight into the interactions between Zn^2+^, H_2_O, and functional groups within the polymer matrix, density functional theory (DFT) calculations were conducted to support the protective mechanism of PDMAG layers on Zn anodes. As shown in Fig. 2j, the binding energy of AM-H_2_O (−0.33 eV) is more negative than DMAA-H_2_O (–0.25 eV), which can be attributed to the presence of hydrophobic –CH_3_ groups in PDMAA that weaken HB with H_2_O. This result further supports that PAM effectively restricts more bound water, thereby inducing excessive interfacial H_2_O. Moreover, the binding energies of PDMAA-Zn^2+^ and PDMAG-Zn^2+^ are higher than those of PAM-Zn^2+^ and PACG-Zn^2+^, which signifies the stronger interaction between Zn^2+^ and –C=O in PDMAA and PDMAG. This favors the regulation of the solvation structure in Zn(H_2_O)_6_^2+^ and facilitates Zn^2+^ diffusion.

To further prove the coordination interactions between polymer and Zn^2+^, electrostatic potential (ESP) maps were subsequently utilized to validate the electronegativity of the –C=O functional groups in PAM and PDMAA. As shown in Fig. 2k, the –C=O groups in PDMAA exhibit a more negative ESP value (−2.45 eV) than that in PAM (−1.91 eV), suggesting that the PDMAG polymer protective layer with more electronegative −C=O groups interacts more strongly with Zn^2+^, thereby regulating Zn^2+^ solvation and facilitating homogeneous deposition. A series of spectroscopic characterizations were conducted to further elucidate the Zn^2+^ coordination and desolvation modulation mechanism with the polymer protective layer. The ^1^H NMR spectra of the –CH- protons (Fig. S9) reveal a pronounced upfield shift in PDMAG-Zn^2+^ relative to PACG-Zn^2+^, indicating the increased electron density around the –CH– groups, which reflects the stronger Zn^2+^ coordination with the electron-rich –C=O groups in PDMAG [33, 34]. Fourier transform infrared (FTIR) spectra and the corresponding second-derivative curves (Figs. S10 and 2l) demonstrate that the peak for –C=O at 1607.8 cm^−1^ shifts to 1605.7 cm^−1^ upon Zn^2+^ introduction in PACG, confirming the presence of coordination interactions between Zn^2+^ and –C=O in PAM [35]. Notably, compared with PACG, PDMAG exhibits a redshift of the –C=O stretching band from 1604.4 to 1600.2 cm^−1^ upon Zn^2+^ introduction, evidencing stronger Zn^2+^ coordination with PDMAG. Such coordination effectively modulates Zn^2+^ solvation, thereby promoting transport and guiding uniform deposition. To further verify the coordination interaction between –C=O groups in PDMAG and Zn^2+^, XPS analysis was performed. As shown in Fig. S11, the O 1*s* XPS spectra of –C=O and Zn–O shift from 532.2 and 531.5 eV in PACG-Zn^2+^ to higher binding energies of 532.5 and 531.8 eV in PDMAG-Zn^2+^, whereas the Zn 2*p* peaks of PDMAG display a distinct shift toward lower binding energies. These results essentially support the formation of PDMAG-Zn^2+^ solvation structures that effectively regulate Zn^2+^ flux and enable stable Zn deposition.

As presented in Fig. S12, the Raman region at 1020–1050 cm^−1^ reflects the aggregation behavior of OTf^−^ anions by capturing the stretching of sulfonyl (–SO_3_) groups. Characteristic peaks at 1030.2, 1033.6, and 1037 cm^−1^ correspond to free anions (FA), solvent-separated ion pairs (SSIP, Zn^2+^-H_2_O-OTf^−^), and contact ion pairs/aggregates (CIP/AGG, Zn^2+^-OTf^−^, Zn^2+^-OTf^−^-Zn^2+^), respectively [36]. SSIP is the dominant solvation structure in both the Zn(OTf)_2_ electrolyte and PACG-Zn^2+^ hydrogels, whereas the zincophilic (-N(CH_3_)_3_^+^, –C=O) and hydrophobic -CH_3_ groups in PDMAG strengthen the Zn^2+^-OTf^−^ interactions, favoring CIP/AGG formation. Accordingly, PDMAG is preferable for modulating the Zn^2+^ solvation structure and effectively suppressing parasitic reactions [37]. The process of desolvation for Zn^2+^ constitutes a main hindrance to charge transport. The activation energy (*E*_*a*_) shown in Fig. S13 is approximated as a descriptor of the energy barrier for the desolvation step of hydrated Zn^2+^. Based on electrochemical impedance spectroscopy (EIS) measurements at various temperatures (Fig. S14), PDMAG@Zn exhibits a lower *E*_*a*_ (26.2 kJ mol^−1^) compared to bare Zn (48.6 kJ mol^−1^) and PACG@Zn (39.3 kJ mol^−1^). This reduction in *E*_*a*_ indicates that PDMAG effectively modulates the Zn^2+^ solvation structures, thereby facilitating the Zn^2+^ desolvation process and improving ion transport kinetics [38].

In situ Raman spectroscopy measurement was utilized to monitor the evolution of Zn^2+^ at the electrode/electrolyte interface during the deposition process. The Raman signals at 768 and 1064 cm^−1^ originate from νs (-CF_3_) and νs (SO_3_^−^) [39, 40], which can be utilized to ascertain changes in the local Zn^2+^ concentration [41]. As shown in Fig. 2m–o, bare Zn exhibits a rapid and pronounced decrease in the Raman intensities of both -CF_3_ and SO_3_^−^ during Zn deposition, reflecting a highly inhomogeneous Zn^2+^ ion flux near the electrode/electrolyte interface. While the variation in Raman intensity is alleviated with PACG@Zn, noticeable fluctuations still can be observed. In contrast, PDMAG@Zn exhibits negligible signal intensity variation, suggesting that the enhanced coordination between Zn^2+^ and the –C=O groups in PDMAG regulates a uniform and homogeneous Zn^2+^ distribution at the electrode/electrolyte interface throughout the deposition process.

### Side Reactions Inhibition Behavior and Electrochemical Analysis

**Fig. 3:**
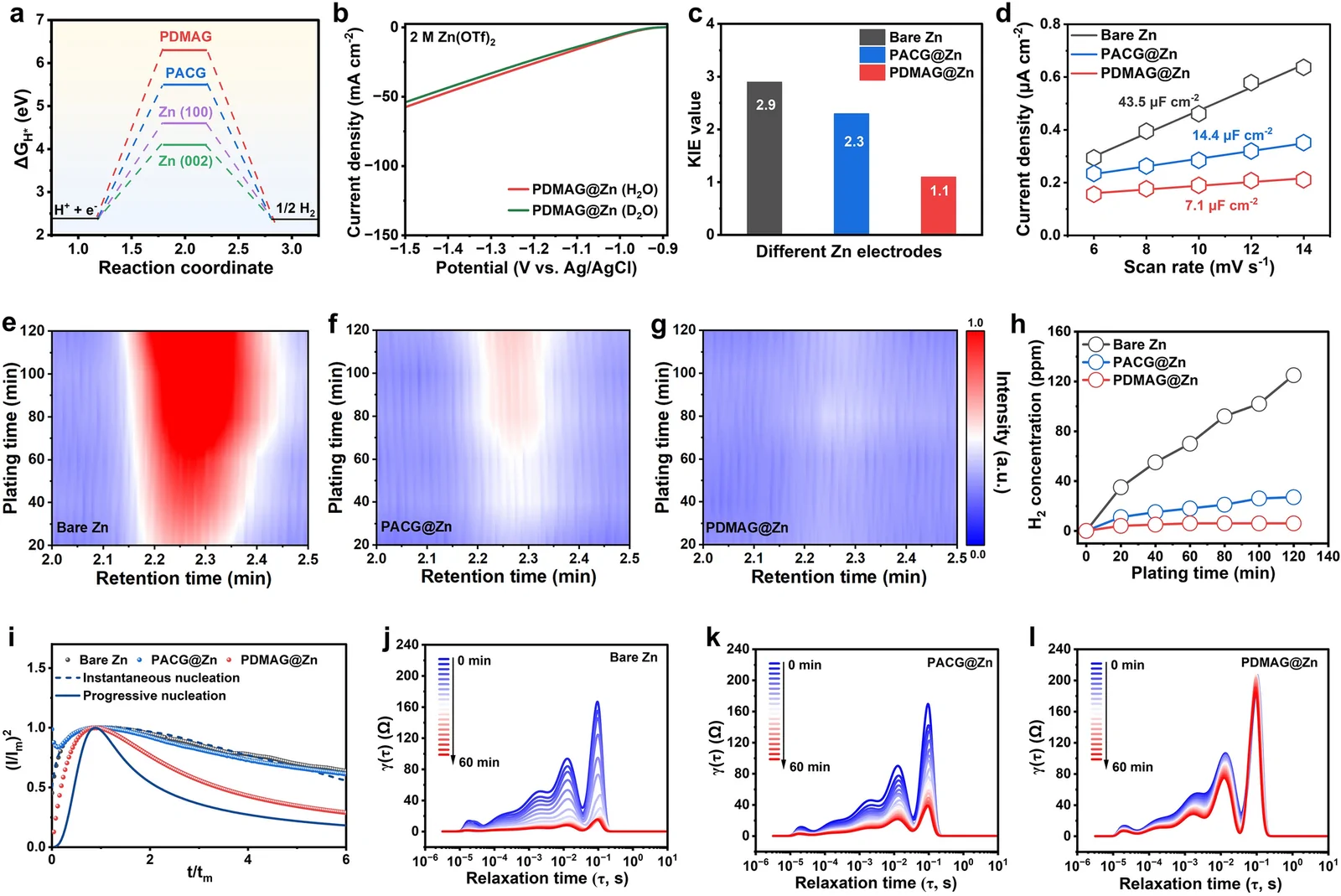
Side reaction inhibition evaluation. **a** The Gibbs free energy on bare Zn, PACG and PDMAG for HER. **b** LSV curves of PDMAG@Zn in 2 M Zn(OTf)_2_ (H_2_O and D_2_O) electrolytes. **c** Calculated KIE values. **d** EDLC for bare Zn, PACG@Zn, and PDMAG@Zn. In situ EC-GC profiles of the Zn plating process with **e** bare Zn, **f** PACG@Zn, and **g** PDMAG@Zn. **h** The comparison of different electrodes on the evolution of H_2_ concentration during Zn plating at 10 mA cm^−2^. **i** Comparison of experimental dimensionless transients with theoretical 3D nucleation models for different Zn electrodes. DRT analysis of EIS spectra during Zn deposition for** j** bare Zn, **k** PACG@Zn, and **l** PDMAG@Zn by assembling symmetrical Zn batteries

In bulk electrolyte, parasitic reactions typically compete for active sites on the Zn anode, hindering Zn deposition and elevating the local OH^−^ concentration, which promotes the formation of undesirable by-products. DFT calculations were conducted to elucidate the underlying mechanism by which the PDMAG interface suppresses the HER, through a comparative analysis of the Gibbs free energy for H adsorption (ΔG_H_*) at different interfaces. As depicted in Fig. 3a, the PDMAG owns a higher ΔG_H_* (6.3 eV) than that of bare Zn (5.0 eV) and PACG (5.5 eV), indicating the intrinsic capacity of PDMAG on suppressing interfacial proton reduction and mitigating H_2_ evolution [42]. The effect of hindered proton transfer on HER dynamics was subsequently verified by a series of extensive electrochemical characterizations. From the LSV curves shown in Fig. S15, the PDMAG@Zn exhibits the lowest onset potential for HER compared to bare Zn and PACG@Zn, implying that the employed PDMAG@Zn effectively suppresses the interfacial proton transport and retards the HER kinetics. The Tafel curves in Fig. S16 reveal that the symmetrical batteries with PDMAG@Zn presented a higher corrosion potential (−0.86 V) and a lower corrosion current density (5.2 mA cm^−2^) than those with bare Zn (−0.97 V, 47.8 mA cm^−2^) and PACG@Zn (−0.91 V, 18.6 mA cm^−2^), indicating that PDMAG@Zn provides enhanced stability and corrosion resistance [43, 44]. To further determine the ability of PDMAG to inhibit the continuous proton transport by non-covalent interactions with interfacial H_2_O molecules, kinetic isotope effect (KIE) experiments were conducted [45]. Generally, higher KIE values signify a greater contribution of proton transport via the "Grotthuss" mechanism to the HER. As shown in Figs. S17 and 3b, when H_2_O was substituted with D_2_O in 2 M Zn(OTf)_2_ electrolyte, the HER activity of bare Zn and PACG@Zn decreased, whereas the PDMAG@Zn exhibits almost overlapped curves both in H_2_O and D_2_O electrolytes, suggesting minimal proton involvement in the interfacial reaction. Consistently, the corresponding KIE values (J_D2O_/J_H2O_ at −1.0 V vs Ag/AgCl) calculated in Fig. 3c confirm that PDMAG@Zn owns the lowest KIE values of 1.1 compared to bare Zn (2.9) and PACG@Zn (2.3). These results reveal that the HER on bare Zn and PACG@Zn is influenced by the Grotthuss-type proton transport mechanism. In this process, H^+^ transport from the electrolyte to the electrode surface is facilitated by non-covalent H-bond networks between interfacial H_2_O-H_2_O and hydrophilic polymer-H_2_O, resulting in limited HER suppression. In contrast, PDMAG@Zn demonstrates a proton-blocking interfacial characteristic, which suppresses the HER activity. Considering the "Grotthuss" mechanism, the intrinsic HER activity of PAM and PDMAA was compared using PAM@NF and PDMAA@NF electrodes in 0.5 M H_2_SO_4_. As shown in the LSV curves (Fig. S18), PAM@NF exhibits a lower overpotential (196 mV at 10 mA cm^−2^) than bare NF (Ni foam) (332 mV) and PDMAA@NF (350 mV), confirming its superior HER activity via rapid H^+^ transport through continuous HB network. Linear sweep voltammetry (LSV) tests (Fig. S19) were utilized to assess the electrochemical stability windows (ESW) of different electrodes. PDMAG@Zn exhibits the broadest ESW of 2.72 V compared to 2.61 V for PACG@Zn and 2.50 V for bare Zn, attributing to its capability to suppress the side reactions.

The energy levels of the highest occupied molecular orbital (HOMO) and the lowest unoccupied molecular orbital (LUMO) energy levels for polymers were provided in Fig. S20. As depicted, PDMAG exhibits the narrowest energy level gap (4.66 eV) compared to H_2_O (9.53 eV) and PACG (5.54 eV). The reduced band gap represents enhanced electron transfer ability and therefore a greater tendency for PDMAG to absorb onto the Zn electrode surface [46]. Moreover, as the electric double-layer capacitance (EDLC) measurements revealed in Figs. 3d and S21, PDMAG@Zn exhibits the lowest capacitance value (7.1 μF cm^−2^) compared to bare Zn (43.5 μF cm^−2^) and PACG@Zn (14.4 μF cm^−2^). The reduced EDLC reveals that the PDMAG protective layer could effectually decrease the electrochemically active area of the Zn electrode, thereby suppressing the side reaction. Additionally, differential capacitance tests further confirm that the decreased interfacial capacitance is attributed to strong adsorption of PDMAG layer on Zn surface, thereby increasing the thickness of EDL [47] (Fig. S22). In situ Raman spectra of H_2_O signals were performed to comprehensively monitor the dynamic evolution and distribution of interfacial H_2_O at the Zn surfaces [48, 49]. As demonstrated in Fig. S23, the peak between 3000 and 3800 cm^−1^ corresponds to ν(O–H) stretching vibrations of H_2_O. Notably, the interfacial H_2_O signal of PDMAG@Zn/electrolyte represent a significant reduced intensity compared to bare Zn and PACG@Zn during the constantly Zn plating process, indicating the capacity of moderately hydrophobic PDMAG layer to suppress interfacial water accumulation and mitigate side reactions. In situ electrochemical gas chromatography (EC-GC) was exploited to further track the practicing H_2_ evolution during the Zn plating process at a constant current density of 10 mA cm^−2^. As illustrated in Figs. 3e–g and S24, a pronounced H_2_ signal was clearly observed for both bare Zn and PACG@Zn within the consecutive 120 min of plating, whereas H_2_ evolution was nearly undetectable for PDMAG@Zn. The corresponding temporal evolution of H_2_ concentration is detailed in Fig. 3h. Furthermore, the as calculated Faraday efficiency (Fig. S25) of PDMAG@Zn during the whole plating time is much lower than that with bare Zn and PACG@Zn. These in situ quantitative results provide direct evidence that the PDMAG@Zn could effectively suppress the HER during actual battery operation [50].

A fundamental mechanistic understanding of Zn nucleation behavior is critical for controlling dendritic growth, as it directly influences the subsequent deposition morphology and interfacial stability. The CV tests were conducted to obtain the nucleation overpotentials (NOP) for different electrodes **(**Fig. S26). PDMAG@Zn exhibits a higher NOP (82 mV) than bare Zn (61 mV) and PACG@Zn (64 mV), suggesting a fine-grained Zn deposition with favorable crystallographic orientation. SEM images (Fig. S27) further confirm that PDMAG@Zn produces smaller and more uniform nuclei, whereas bare Zn and PACG@Zn show irregular and coarse nucleation. This reduced nucleation size promotes homogeneous Zn deposition, effectively suppressing dendrite formation and directing oriented crystal growth. To elucidate the nucleation behavior during Zn deposition, the experimentally measured chronoamperometric (CA) curves were fitted by utilizing the Scharifker-Hills (S–H) models to compare the dimensionless experimental transients with the theoretical 3D nucleation models [51]. As shown in Fig. 3i, bare Zn and PACG@Zn exhibit instantaneous nucleation behavior, whereas the PDMAG@Zn exhibits a progressive nucleation process characterized by the formation of homogeneous, densely packed nuclei. This nucleation mode achieves dendrite-free deposition and ensures regulated Zn^2+^ ion flux. Furthermore, distribution of relaxation times (DRT) analysis [52] was subsequently applied to interpret the interfacial kinetics based on the in situ EIS results of symmetrical Zn batteries. As shown in Figs. 3j-l and S28, both the charge-transfer resistance (R_ct_) and the diffusion resistance (R_diffusion_) of PDMAG@Zn rapidly stabilize after a few cycles during the plating process and exhibit smaller relaxation time (τ) values compared to bare Zn and PACG@Zn. Distinct from the detrimental localized low impedance of bare Zn, the beneficial homogenized impedance of the polymer layer moderately increases the overall charge-transfer barrier to suppress localized Zn^2+^ deposition at hotspots and promote uniform planar growth [53, 54]. This further confirms the robust interfacial stability, which ensures uniform Zn nucleation. The SEM images (Fig. S29) of Zn anode with PDMAG layer illustrate that the polymer film remains intact on top of the deposit after plating at 1 mA cm^−2^ and 10 mAh cm^−2^, further confirming that it genuinely functions as a protective SEI to ensure interfacial stability. As shown in Fig. S30, the Zn^2+^ transference number of PDMAG@Zn reaches 0.81, which is higher than that of bare Zn (0.54) and PACG@Zn (0.65). This enhancement suggests that PDMAG effectively reduces the Zn^2+^ desolvation barrier and establishes fast Zn^2+^ diffusion channels to guide the uniform Zn deposition. These findings confirm the enhanced interfacial reaction kinetics and improved Zn^2+^ transport, contributing to reversible Zn deposition and effective dendrite suppression. To verify the importance of moderate hydrophobicity, a highly hydrophobic poly(diacetone acrylamide) (PDAAM) was introduced for comparison. PDAMG@Zn (PDAAM-CGG@Zn) shows a WCA of 123° (Fig. S31), indicating strong hydrophobicity that repels water but hinders Zn^2+^ desolvation and transport. Consequently, it exhibits a lower Zn^2+^ transference number (0.62) (Fig. S32), confirming excessive hydrophobicity degrades Zn^2+^ transport. To further visualize the ability on dendrite inhibition and anti-corrosion of PDMAG@Zn, Zn electrodes were separately soaked in 2 M Zn(OTf)_2_ electrolytes for various days. The SEM images of PDMAG@Zn shown in Fig. S33 invariably exhibit smooth and uniform morphologies even after soaking for 7 days, evidencing the capacity of PDMAG@Zn for corrosion resistance. Contrarily, bare Zn and PACG@Zn display varying degrees of dendrite and by-product accumulation. The anti-corrosion of Zn electrodes was further quantified by asymmetrical Zn||Ti batteries [55]. As exhibited in Fig. S34, complete Zn depletion occurred after 200 h for bare Zn, 390 h for PACG@Zn, and nearly 700 h for PDMAG@Zn. The corresponding corrosion rates were calculated as 3.5 μg h^−1^ for PDMAG@Zn, which is lower than those of bare Zn (12 μg h^−1^) and PACG@Zn (6.6 μg h^−1^). These results clearly demonstrate the superior anti-corrosion capability of PDMAG@Zn in effectively protecting Zn anode.

### Evaluation of Zn Plating/Stripping Performance

**Fig. 4:**
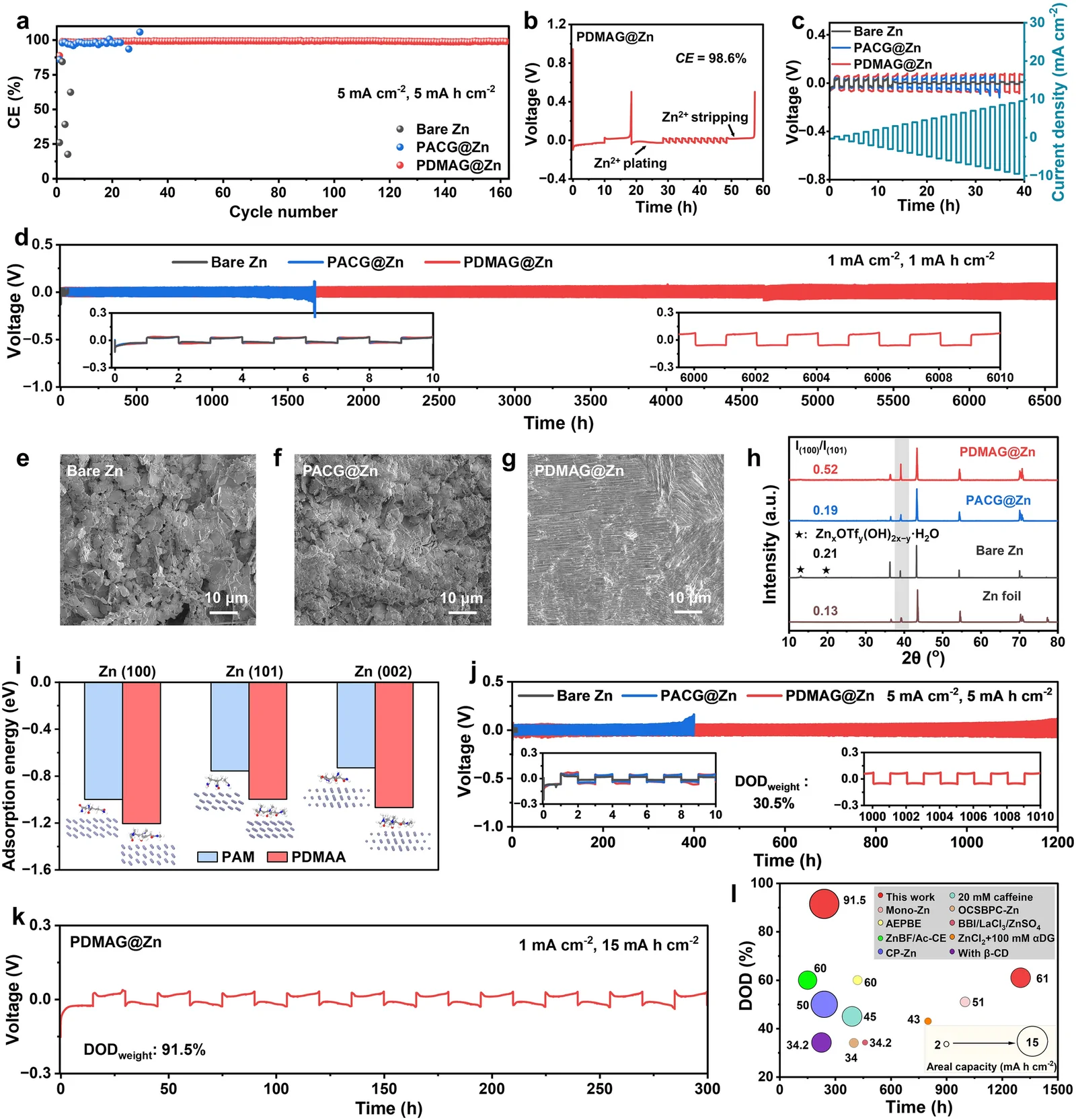
Performances for symmetrical batteries. **a** CE performance of the Zn||Cu batteries at 5 mAh cm^−2^, 5 mAh cm^−2^. **b** Reservoir-based Zn||Cu batteries for assessing average Zn stripping/plating CE of PDMAG@Zn. **c** Voltage evolution at step-increased current densities. **d** The performance of symmetrical Zn batteries at 1 mA cm^−2^, 1 mAh cm^−2^. SEM images of **e** bare Zn, **f** PACG@Zn, and **g** PDMAG@Zn after 100 h cycling at 1 mA cm^−2^. **h** XRD spectra after 100 h cycling. **i** Adsorption energy of PAM and PDMAA on the Zn (100), (101), and (002) planes. **j** The performance of symmetrical Zn batteries at 5 mA cm^−2^, 5 mAh cm^−2^. **k** The performance of symmetrical PDMAG@Zn battery at 1 mA cm^−2^, 15 mAh cm^−2^.** l** Comparison of DOD, cycling time, and areal capacity in this work with previous reports

Coulombic efficiency (CE) is a critical metric for assessing the reversibility of Zn^2+^ during the plating/stripping process. As exhibited in Fig. 4a, the CE of Zn||Cu batteries with bare Zn and PACG@Zn rapidly declines within the initial few cycles due to uncontrolled dendrite growth and severe side reactions. In contrast, the PDMAG@Zn||Cu asymmetrical battery exhibits stable cycling over 320 h at 5 mA cm^−2^, 5 mAh cm^−2^, maintaining a high average CE of 99.8%. To quantify CE more accurately, the reservoir-based Zn||Cu battery testing procedure [51] was applied (Fig. 4b). Specifically, a Zn layer with a capacity of 10 mAh cm^−2^ was first pre-deposited onto the Cu electrode, followed by complete stripping. The PDMAG@Zn achieves a CE of 98.6%, substantially higher than that of bare Zn and PACG@Zn (Fig. S35). Additionally, 10 mAh cm^−2^ of Zn was deposited onto the Cu electrode and stripped over 10 cycles at 1 mA cm^−2^, 1 mAh cm^−2^. These results further confirm the effective corrosion inhibition capability of PDMAG@Zn. As shown in Fig. 4c, the limiting current density was further evaluated by stepwise increases from 0.25 mA cm^−2^ (0.25 mAh cm^−2^) to 10 mA cm^−2^ (10 mAh cm^−2^) [33]. The battery with PDMAG@Zn exhibits enhanced rate capability, maintaining stable charge/discharge voltage profiles across the entire range of current densities progressively up to 10 mA cm^−2^. Nevertheless, both bare Zn and PACG@Zn suffer from severe overpotential accumulation and short-circuiting after only a few cycles. These results further underscore the fast Zn^2+^ transport kinetics and favorable electrochemical reversibility conferred by the PDMAG protective layer, consistent with the rate performance trends presented in Fig. S36.

Symmetrical Zn batteries were operated to evaluate the electrochemical reversibility and long-term stability of Zn anodes. As illustrated in Fig. 4d, the symmetrical battery with PDMAG@Zn demonstrates excellent cycling stability for over 6580 h (> 9 months) at the current density of 1 mA cm^−2^ and 1 mAh cm^−2^. SEM images of Zn electrodes after 100 h of cycling were examined in Fig. 4e-g. As illustrated, the bare Zn and PACG@Zn present rough surfaces with dendritic protrusions and notable by-product accumulation. In contrast, PDMAG@Zn maintains a compact and uniform surface morphology with a regulated crystallographic orientation. Cross-sectional SEM analysis of the PDMAG@Zn electrode was performed after 500 h of cycling at 1 mA cm^−2^ and 1 mAh cm^−2^. The results (Fig. S37) show that remains intact and uniformly adhered to the Zn substrate, continuously functioning as a robust protective layer. XRD analysis was further employed to verify the crystallographic orientations of Zn deposits directed by PDMAG. As depicted in Fig. 4h, apparent by-product peaks related to Zn_x_(OTf)_y_(OH)_2x-y_ꞏH_2_O appear after cycling, but no obvious signals are monitored in PDMAG, suggesting its superior ability in anti-corrosion. Furthermore, the Zn (100) plane becomes more pronounced after cycling with PDMAG compared to PACG and BE, revealing that PDMAG guides Zn deposition along the (100) plane and mitigates side reactions. As calculated, the intensity of I_(100)_/I_(101)_ for PDMAG@Zn after normalization is 0.52, which is higher than bare Zn (0.21) and PACG@Zn (0.19). Contributing to the preferential adsorption on Zn (100) plane, PDMAG induces the formation of Zn (100) plane with intrinsically high growth velocity. Moreover, as the XRD spectra presented in Fig. S38, the intensity of Zn (100) plane signals also exhibits a trend in parallel with the increase in deposition capacity from 1 to 15 mAh. This promotes rapid Zn^2+^ stripping/plating kinetics and enables extended cycling stability even under demanding conditions of high current densities and large areal capacities. Additionally, as provided in Fig. 4i, the adsorption energy of PDMAA on the Zn (100) crystal plane is -1.205 eV, lower than that on Zn (101), Zn (002), and PAM-coated Zn surfaces, respectively. This preferential adsorption on the Zn (100) plane corroborates the experimental observation that the PDMAG protective layer directs Zn deposition toward the (100) plane. According to the Wuff construction principle, the preferential adsorption of PDMAA on Zn (100), lowering its surface energy relative to other planes and making it thermodynamically favored for exposure [56, 57]. During initial deposition, Zn^2+^ nucleates preferentially on the stabilized (100) plane. Subsequently, PDMAA continuously stabilizes (100) nuclei and promotes epitaxial growth, maintaining the persistent (100) texture during the stripping/plating process. AFM images further demonstrate the convincing ability of the PDMAG protective layer to regulate Zn^2+^ flux distribution for uniform Zn deposition, even under electroplating at 10 mA cm^−2^ for 1 h (Fig. S39). Additionally, the symmetrical PDMAG@Zn batteries manifest long-term stability, operating for 2350 h at 3 mA cm^−2^ and 3 mAh cm^−2^ (Fig. S40) and 1200 h at 5 mA cm^−2^ and 5 mAh cm^−2^ with 30.5% DOD_Weight_ (Fig. 4j) separately. The shelving-recovery measurements were further conducted to evaluate the practical applicability of batteries. Impressively, the symmetrical Zn batteries with PDMAG@Zn with 40 h rest period after every 20 cycles remain stable for more than 5800 h at 1 mA cm^−2^ and 1 mAh cm^−2^ (Fig. S41), whereas bare Zn and PACG@Zn undergo severe deterioration after only 35 and 430 h cycling, respectively. Meanwhile, PDMAG@Zn batteries also exhibit robust shelving-recovery performance for over 810 h at 5 mA cm^−2^ and 5 mAh cm^−2^, thereby confirming the boosted resistance to side reactions under practical conditions (Fig. S42).

The cycling performance at high areal capacities and deep depth of discharge (DOD_Weight_) is essential for evaluating Zn metal utilization in practical applications [4]. As illustrated in Fig. S43, the symmetrical PDMAG@Zn battery delivers a superior lifespan of 1270 h with 61% DOD_Weight_ at 1 mA cm^−2^, 10 mAh cm^−2^, attributing to the diminished Zn anode corrosion. Moreover, when the areal capacity was further increased to 15 mAh cm^−2^, the symmetrical PDMAG@Zn battery maintains steady cycling for 300 h, realizing a high DOD_Weight_ of 91.5% (Fig. 4k). The SEM images (Fig. S44) show the morphologies of PDMAG@Zn operated under 1 mA cm^−2^, 15 mAh cm^−2^ conditions, where the initial stripping (1–15-S)/plating (1–15-P) sides of Zn surfaces and the sides (1cycle-S, 1cycle-P) after 1 cycle retain the smooth and flat morphology characteristics after different cycles. This result underscores that by confining interfacial H_2_O via weak HB interactions and promoting rapid Zn^2+^ stripping/plating along the (100) plane, PDMAG ensures high Zn utilization and sustained reversibility throughout the entire process. Combined with the XRD spectra (Fig. S45), the Zn electrodes show a gradual increase in orientation along the Zn (100) plane during the stripping/plating process, with the I_(100)_/I_(101)_ for PDMAG@Zn is from 1 to 15S (0.24) to 3 cycles (0.50). This signifies that the PDMAG induces Zn (100) plane with fast ion reaction kinetics, promoting easier Zn^2+^ stripping/plating behavior for compacted deposition and effective side reaction suppression. Furthermore, symmetrical PDMAG@Zn batteries can also achieve high-rate cycling stability, sustaining 1000 h at 10 mA cm^−2^, 5 mAh cm^−2^ and 210 h at 10 mA cm^−2^,10 mAh cm^−2^ with high Zn utilization of 30.5% and 61%, respectively (Fig. S46). These results underscore that the PDMAG protective layer can effectively guide the uniform Zn deposition even under constant high Zn^2+^ flux, while simultaneously suppressing parasitic reactions and ensuring high cycling reversibility to satisfy the high Zn utilization. Furthermore, Raman spectra of cycled PDMAG@Zn (10 mA cm^−2^, 10 mAh cm^−2^) were compared with pristine Zn. As exhibited, no new organic–inorganic hybrid peaks were observed after cycling (Fig. S47), which indicate the CGG functions through direct electrostatic adsorption to homogenize interfacial Zn^2+^ flux and electric-field distribution for uniform Zn nucleation and deposition [58], excluding an anion-mediated mechanism. Additionally, cross-sectional SEM image of PDMAG@Zn after 30 cycles at 10 mA cm^−2^, 10 mAh cm^−2^ (Fig. S48) depicts that after extended cycling under high areal capacities, the PDMAG protective layer remains intimately adhered to the Zn substrate without any evidence of delamination or detachment, indicating the robust adhesion of PDMAG interfacial layer. The performances of symmetrical PDMAG@Zn batteries surpass those of most previously reported works in aqueous Zn batteries (Fig. 4l), clearly highlighting that the engineered PDMAG interfacial architecture boosts the electrochemical reversibility and mitigates parasitic degradation to achieve deep cycling of Zn anodes [59–67].

### Electrochemical Performance of the Full Batteries

**Fig. 5:**
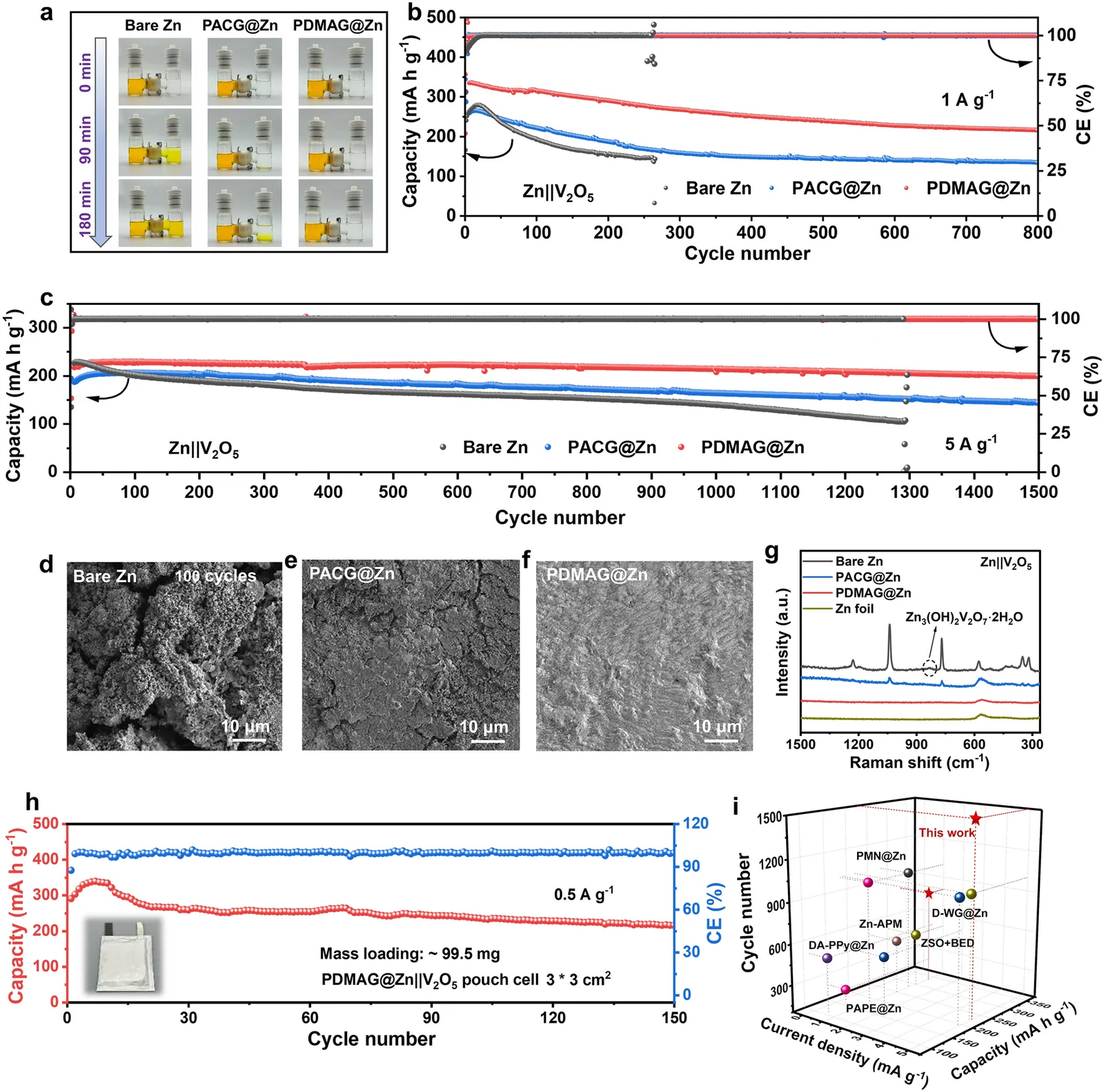
Electrochemical performances of Zn||V_2_O_5_ full batteries. **a** The diffusion tests of soluble vanadate species performed in H-type electrolytic cells with GF, PACG@GF and PDMAG@GF separators. The long-term cycling performance of Zn||V_2_O_5_ batteries at **b** 1 A g^−1^ and **c** 5 A g^−1^. SEM images of **d** bare Zn, **e** PACG@Zn, and **f** PDMAG@Zn after 100 cycles at 5 A g^−1^. **g** The Raman spectra of different Zn anodes after 100 cycles at 5 A g^−1^ in Zn||V_2_O_5_ batteries. **h** The cycling performance for PDMAG@Zn-V_2_O_5_ pouch cell at 0.5 A g^−1^. **i** Comparisons of cycle number and capacities of Zn||V_2_O_5_ batteries in this work and current reported literatures

Generally, V_2_O_5_ and its derivatives are recognized as promising Zn-ion battery cathodes, offering high capacity and long-term cycling stability. As reported, the V_2_O_5_ cathode is prone to dissolution during cycling, generating vanadate anions (V_2_O_7_^4−^). These anions can migrate through the separator and accumulate on the Zn anode surface as vanadium-based by-products, which in turn accelerate the degradation of the anode [68]. To verify the capacity of PDMAG in inhibiting the crossover effect of anions, GF, PACG, and PDMAG hydrogel coated GF separators were separately employed to conduct the diffusion experiments with H-type cells. The left chambers of H-type cells were filled with the pH-regulated V_2_O_5_ solutions containing the generated V_2_O_7_^4−^ species, and the right sides were just filled with deionized water. As demonstrated in Fig. 5a, color changes were observed in the right compartment of the H-type cells with GF and PACG separators after 90 and 180 min, respectively. However, the PDMAG coated GF exhibits the effect on anion diffusion blocking, where the right chamber with deionized water remains colorless and transparent throughout the entire 180-min duration. This result reveals that the strongly electronegative –C=O groups on PDMAA can electrostatically repel dissolved V_2_O_7_^4−^ anions, thereby preventing the vanadate induced by-products and dendrites deposited on the Zn surface. Therefore, this prevention contributes to the enhanced capacity and cycling stability for PDMAG@Zn||V_2_O_5_ batteries. To evaluate the practical applicability of the PDMAG functioned Zn anode, Zn||V_2_O_5_ batteries were assembled. As investigated in Fig. S49, the CV curves of PDMAG@Zn exhibit two typical pairs of redox peaks similar to bare Zn and PACG@Zn, confirming the stability of PDMAG@Zn during the redox reaction process. Likewise, the EIS curves (Fig. S50) reveal that the PDMAG@Zn||V_2_O_5_ battery possesses the lowest R_ct_, which further denotes the superior Zn^2+^||Zn reaction kinetics during charge/discharge processes. The Zn||V_2_O_5_ battery with PDMAG@Zn demonstrates the higher rate capacity with a gradually increased current density from 0.5 to 5 A g^−1^, thereby reflecting the efficient redox kinetics and stable electrode/electrolyte interfacial charge transfer at high current rates (Fig. S51).

Long-term cycling performance is a critical metric for assessing the electrochemical performance of full batteries. As shown in Fig. 5b, Zn||V_2_O_5_ battery assembled with bare Zn exhibits rapid capacity decay after approximately 20 cycles at a current density of 1 A g^−1^, and complete failure occurred after around 250 cycles. Meanwhile, the PACG@Zn||V_2_O_5_ battery delivers a similar initial capacity (~ 250 mAh g^−1^), and suffers poor cycling stability with only 54% capacity retention after 800 cycles. In contrast, the PDMAG@Zn||V_2_O_5_ battery displays a higher specific capacity of 335 mAh g^−1^ and is capable of an admirable capacity retention up to 67% even after 800 cycles. Additionally, when the current density was increased to 5 A g^−1^, the PDMAG@Zn||V_2_O_5_ battery can also achieve a reversible specific capacity of 227 mAh g^−1^ with 89% capacity retention after 1500 cycles (Fig. 5c). However, both the bare Zn and PACG@Zn batteries exhibit lower capacities and suffer from rapid capacity decline after 50 and 300 cycles, respectively. Additionally, as depicted in Fig. S52, a high specific capacity of 322.3 mAh g^−1^ and stable cycling performance are achieved by the PDMAG@Zn||V_2_O_5_ battery, even under a higher mass loading of 7.3 mg cm^−2^ at 0.5 A g^−1^. These results primarily signify that compared to bare Zn and PACG@Zn, the PDMAG may effectively inhibit severe dendrite growth and the accumulation of by-products on the Zn anode surface, contributing to its strong electronegative property for the crossover effect blocking. SEM images were utilized to disclose the morphologies of the Zn anodes after 100 cycles. As illustrated, Zn anodes represent the substantial irregular dendrites and by-products for bare Zn (Fig. 5d) and PACG@Zn (Fig. 5e), whereas the surface of the PDMAG@Zn after cycling delivers a dense and uniform morphology characteristic (Fig. 5f), suggesting the protective effect of the PDMAG layer throughout the extended cycling. To further evidence the effective inhibition of the PDMAG protective layer against the cathode-derived dissolution vanadate species deposition on the Zn anode during cycling, Raman spectroscopy was conducted after 100 cycles at a current density of 5 A g^−1^. As exhibited in Fig. 5g, peaks corresponding to the vanadium-based by-product Zn_3_(OH)_2_V_2_O_7_·2H_2_O (ZVO) [69] were observed on the surface of the bare Zn anode after cycling. This confirms that the localized pH elevation near the cathode caused by the proton intercalation, leading to the dissolution of vanadium species from V_2_O_5_. These species subsequently diffuse through the electrolyte and migrate toward the Zn anode, reacting with Zn^2+^ to form the insoluble ZVO deposits [70]. In contrast, no ZVO-related Raman signals were observed on the PACG@Zn or PDMAG@Zn, representing the anion-shielding ability of polymers. Furthermore, XPS characterizations reveal pronounced vanadium signals on bare Zn and PACG@Zn surfaces (Fig. S53), whereas such signals were absent on PDMAG@Zn after cycling, providing further evidence that PDMAG effectively protects the Zn anode from vanadium-based contamination.

High storage stability is essential to minimize irreversible capacity loss in rechargeable batteries [71]. After 48 h rest (Fig. S54), PDMAG@Zn||V_2_O_5_ retains 94.1% CE, surpassing bare Zn (78.9%) and PACG@Zn (89.4%), demonstrating that the PDMAG layer effectively suppresses side reactions. GITT measurements (Fig. S55) show PDMAG@Zn||V_2_O_5_ exhibits higher Zn^2+^ diffusion (2.81 × 10^–9^ to 2.96 × 10^–7^ cm^2^ s^−1^) than bare Zn (4.46 × 10^–10^ ~ 9.05 × 10^–8^ cm^2^ s^−1^) and PACG@Zn (7.39 × 10^–10^ ~ 2.43 × 10^–7^ cm^2^ s^−1^), reflecting enhanced Zn^2+^ intercalation/deintercalation and long-term cycling stability. To assess the practical applicability of PDMAG@Zn, the Zn||V_2_O_5_ pouch cell with the size of 3 × 3 cm^2^ was tested at 0.5 A g^−1^. As shown in Fig. 5h, the pouch cell still delivers a high initial capacity of 290.5 mAh g^−1^ (28.9 mAh) and maintains the capacity retention of 76% after 150 cycles at a low N/P ratio of 3.6, which convincingly proves the priority of the PDMAG for side reaction suppression. The full battery performance with PDMAG@Zn apparently surpasses the previously reported values for full batteries with V_2_O_5_ cathodes (Fig. 5i) [72–77]. Moreover, the assembled PDMAG@Zn||I_2_ battery with a high mass loading of 13.5 mg cm^−2^ comparably exhibits a high capacity retention of 97.5% after stably performing at the current density of 1 A g^−1^ for over 1100 cycles (Fig. S56). The promising performance of these batteries highlights the broad application potential of PDMAG coating in various Zn-based electrochemical devices.

## Conclusions

In summary, a moderately hydrophobic Zn anode protective layer PDMAG was engineered by an in situ polymerization strategy for AZIBs. The PDMAG polymer with the hydrophobic unit regulates interfacial H_2_O activity and repels H_2_O from the Zn surface, thereby suppressing parasitic reactions. Furthermore, the preferential adsorption of electronegative PDMAA on the Zn (100) plane guides Zn^2+^ deposition with controlled orientation. This directed growth accelerates Zn^2+^ stripping/plating kinetics and ultimately realizes high Zn utilization even under deep cycling conditions. Additionally, the electronegative PDMAG effectively hinders the migration of vanadate anions from the cathode to the Zn anode. This prevents vanadium-based by-products from forming on the Zn surface and thereby effectively improves the performance of full batteries. Consequently, the symmetrical Zn batteries with PDMAG deliver ultra-high Zn utilization, achieving 61% DOD_Weight_ at 10 mA cm^−2^, 10 mAh cm^−2^ for over 200 h. Additionally, the assembled PDMAG@Zn||V_2_O_5_ batteries demonstrate a high specific capacity of 227 mA g^−1^ at 5 A g^−1^, sustaining stable cycling for over 1500 cycles. This work provides a facile and effective strategy for constructing Zn anodes with high Zn utilization and extended energy density in aqueous AZIBs.

## Supplementary Information

Below is the link to the electronic supplementary material.

Supplementary file1 (DOCX 37669 KB)
