# Supplementary material for: Moderately Hydrophobic Polymer Protective Layer Enables Zn (100) Deposition for High Utilization Zinc Anodes
Source: Nanomicro Lett. 2026 May 26;18:386. doi: 10.1007/s40820-026-02208-6 (PMC13201826; doi:10.1007/s40820-026-02208-6)
Supplement: Supplementary file 1 — Supplementary file1 (DOCX 37669 KB) [file 40820_2026_2208_MOESM1_ESM.docx]

Supporting Information for

**Moderately Hydrophobic Polymer Protective Layer Enables Zn (100) Deposition for High Utilization Zinc Anodes**

Xiaoming Fan^1^, Peiyi Wu^1^*, Yucong Jiao^1^*

^1^State Key Laboratory of Advanced Fiber Materials, College of Chemistry and Chemical Engineering, Donghua University, Shanghai 201620, P. R. China

*Corresponding authors. E-mail: [wupeiyi@dhu.edu.cn](mailto:wupeiyi@dhu.edu.cn) (Peiyi Wu); [yucong.jiao@dhu.edu.cn](mailto:yucong.jiao@dhu.edu.cn) (Yucong Jiao)

**Supplementary Figures**


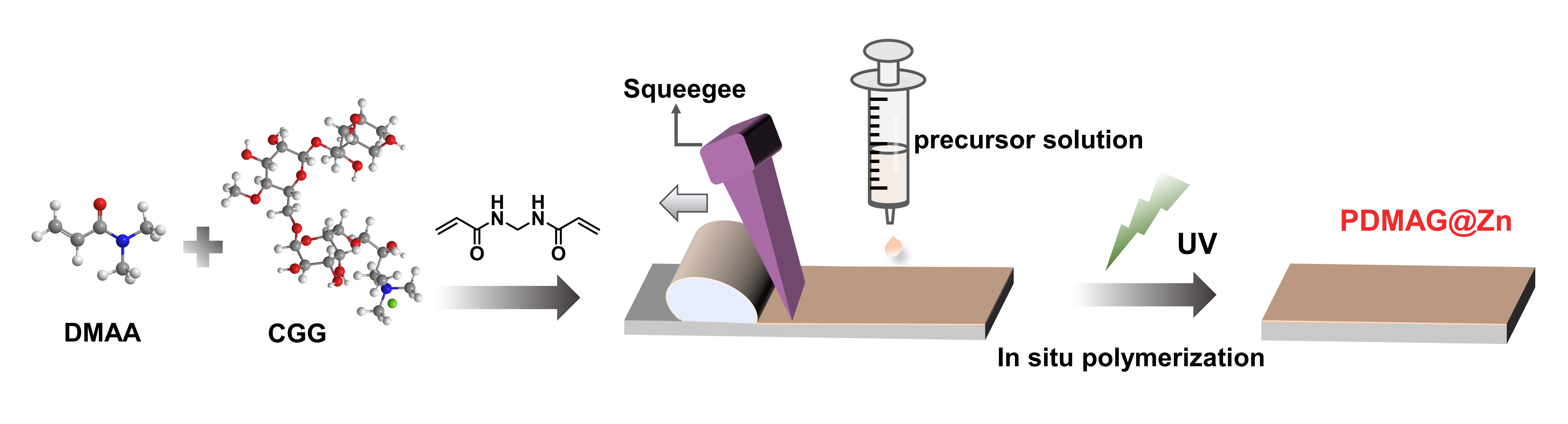


**Fig. S1** Schematic illustration of the preparation process for the PDMAG@Zn

**
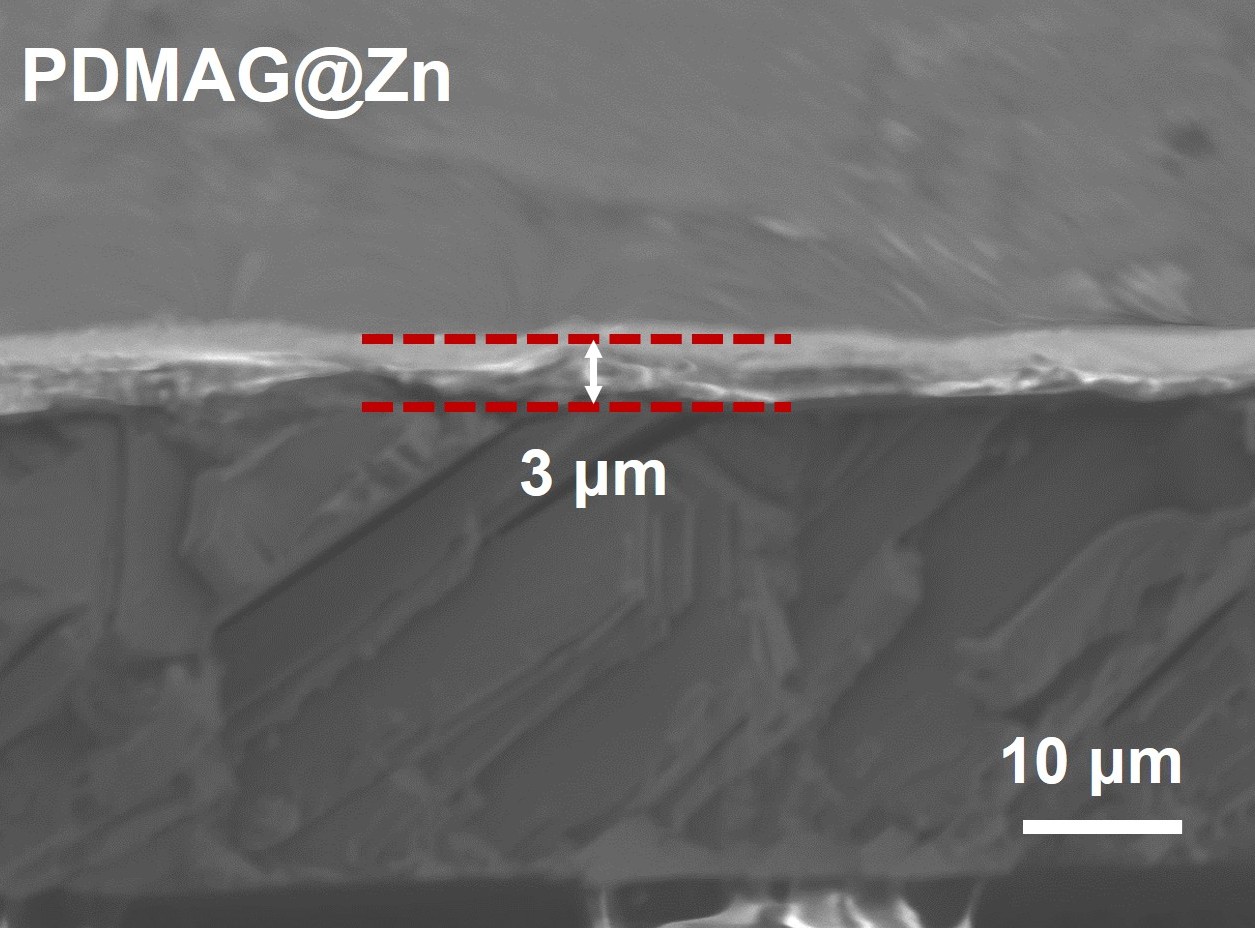
**

**Fig. S2** Cross-sectional SEM image of PDMAG@Zn

**
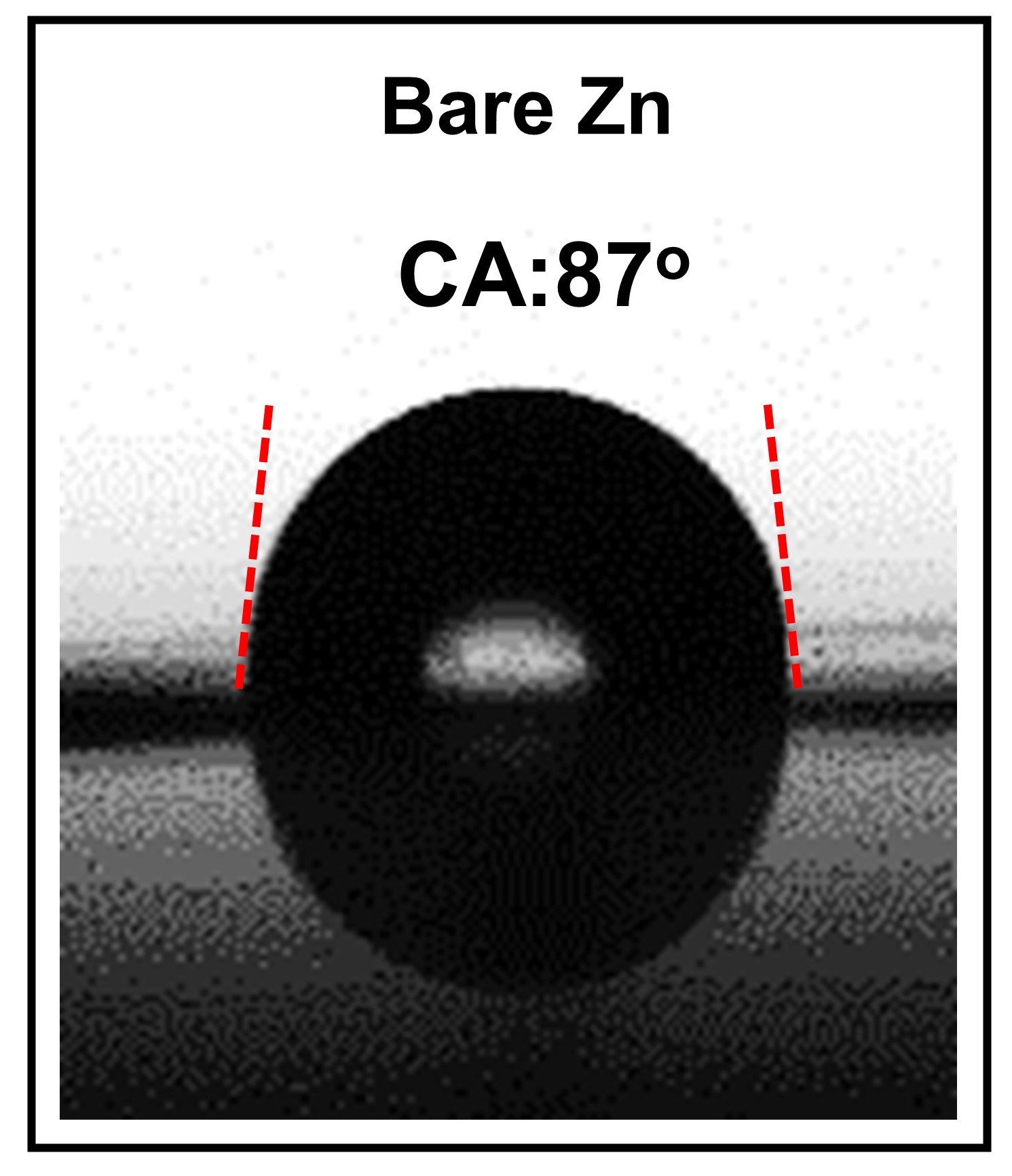
**

**Fig. S3** Contact angle test of bare Zn


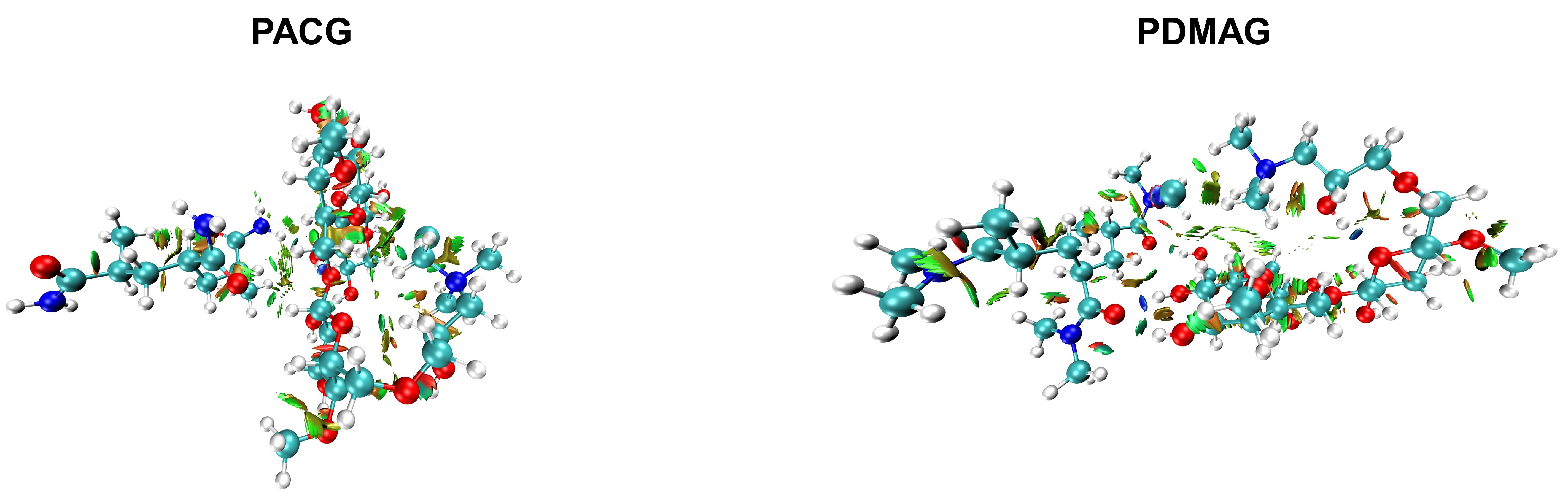


**Fig. S4** The corresponding spatial distribution maps of PACG and PDMAG


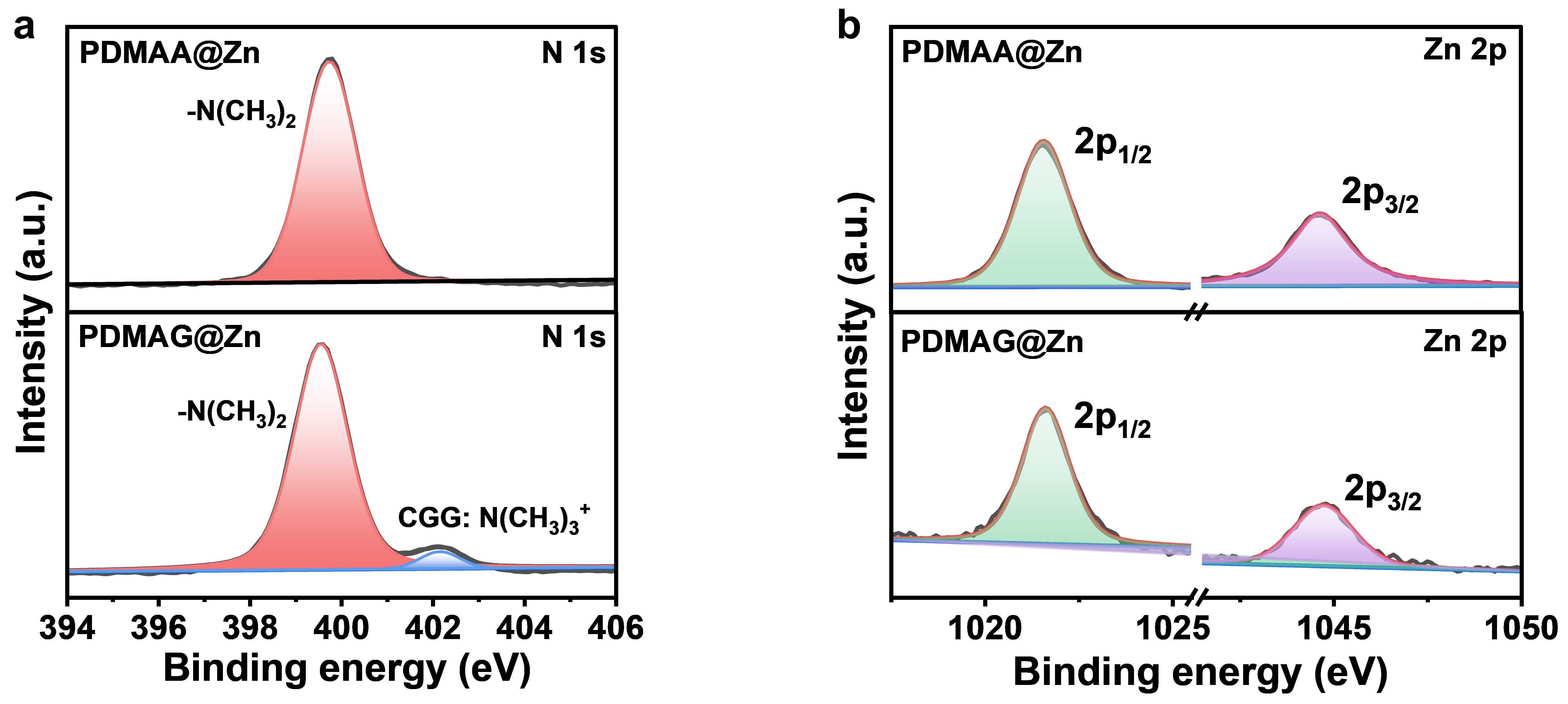


**Fig. S5** The XPS spectra of **a** N 1s and **b** Zn 2p with PDMAA@Zn and PDMAG@Zn


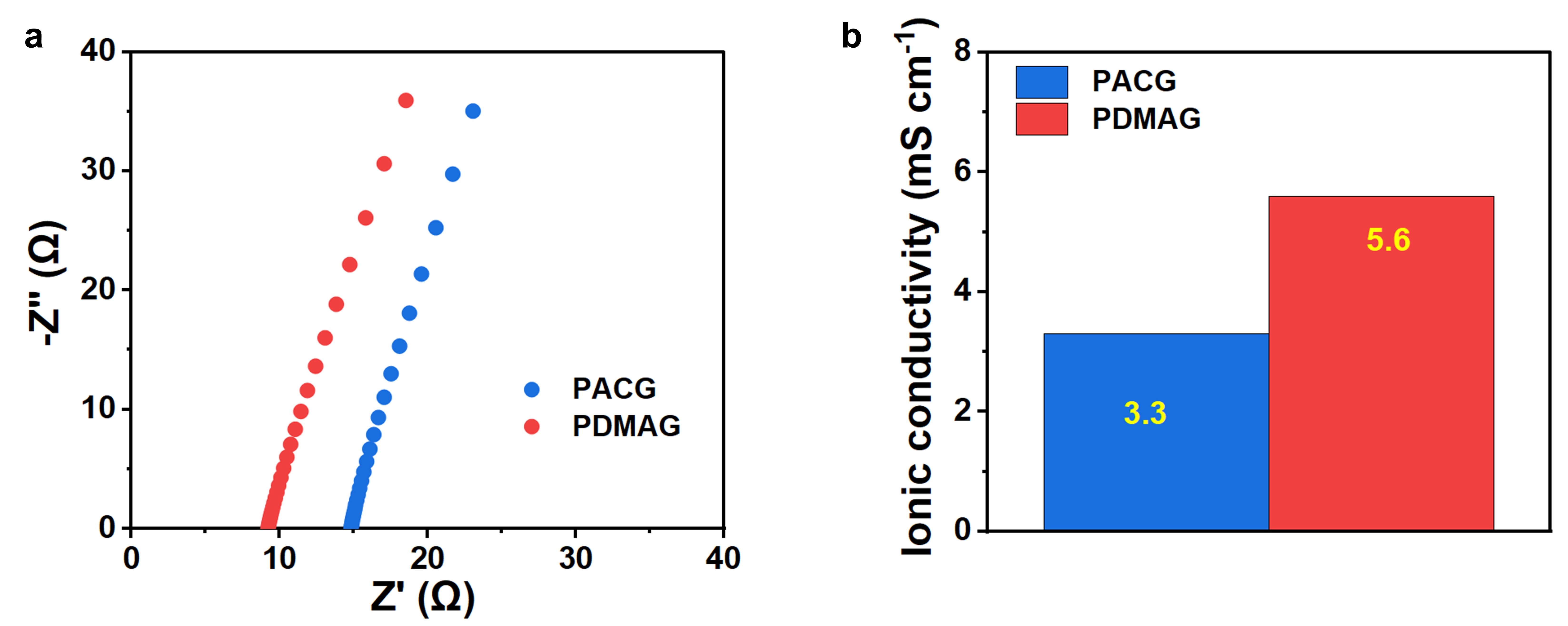


**Fig. S6** Ionic conductivity tests of PACG and PDMAG


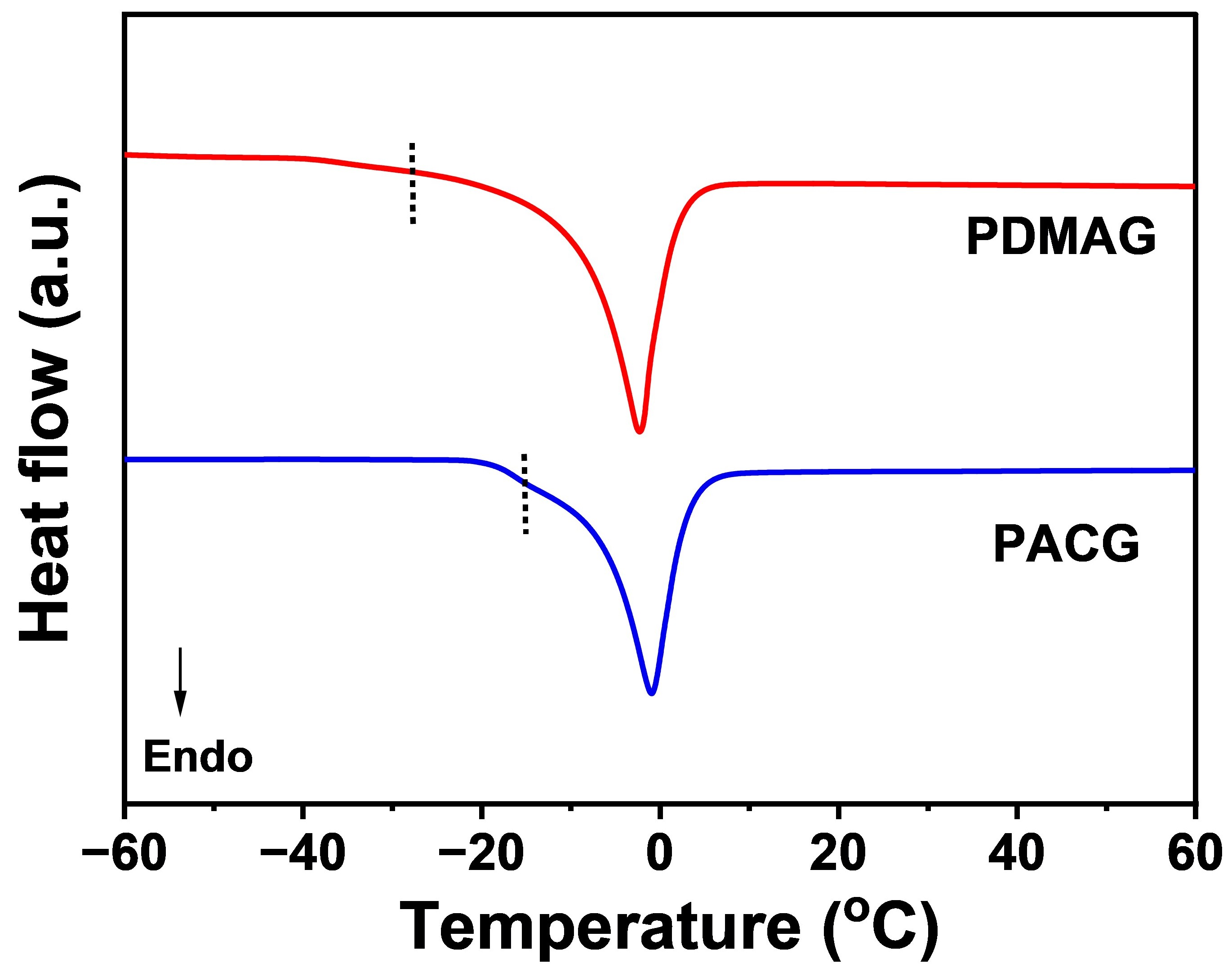


**Fig. S7** DSC measurements of PDMAG and PACG hydrogels

**
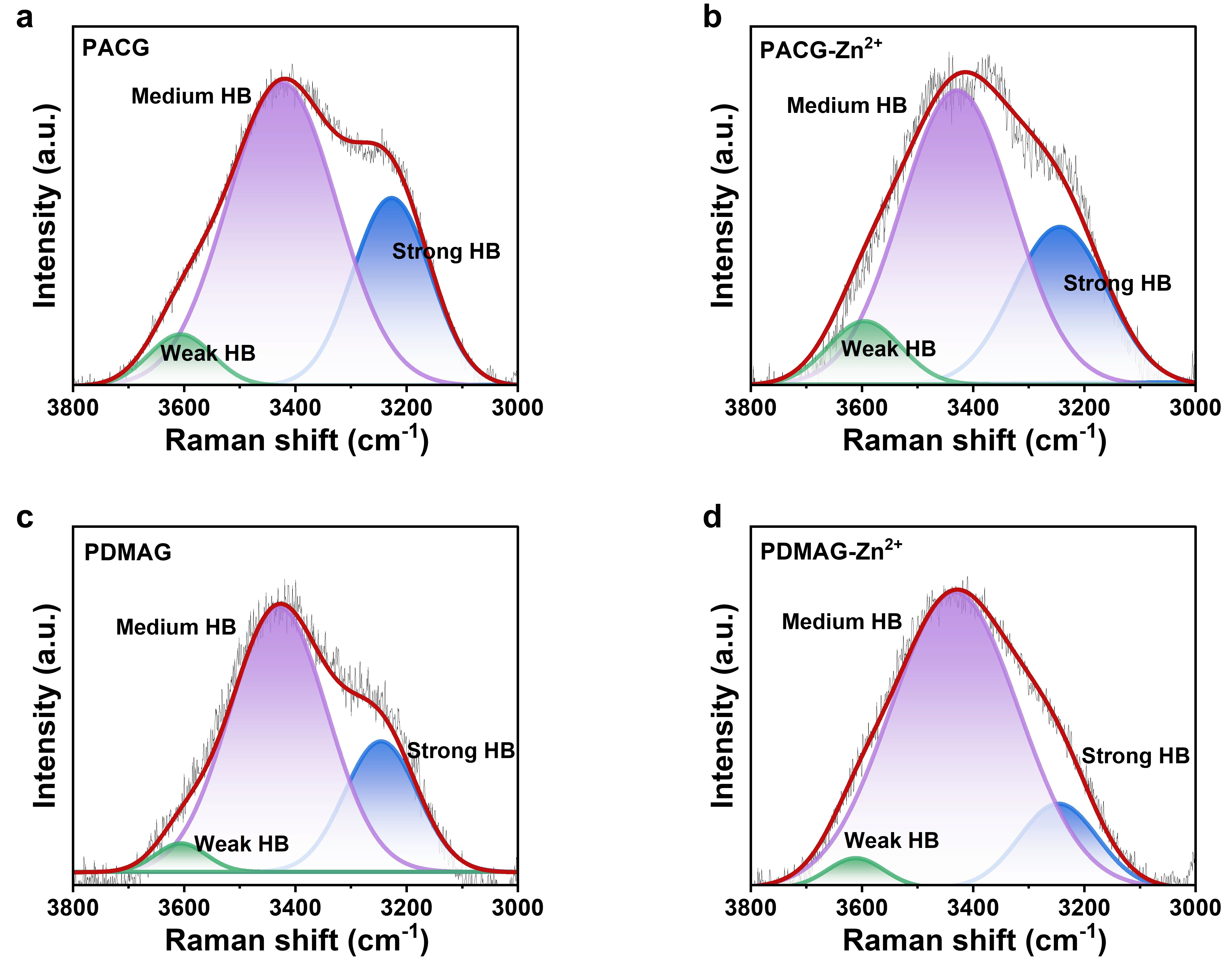
**

**Fig. S8** The Raman spectra peak fittings of **a** PACG, **b** PACG-Zn^2+^, **c** PDMAG, and **d** PDMAG-Zn^2+^


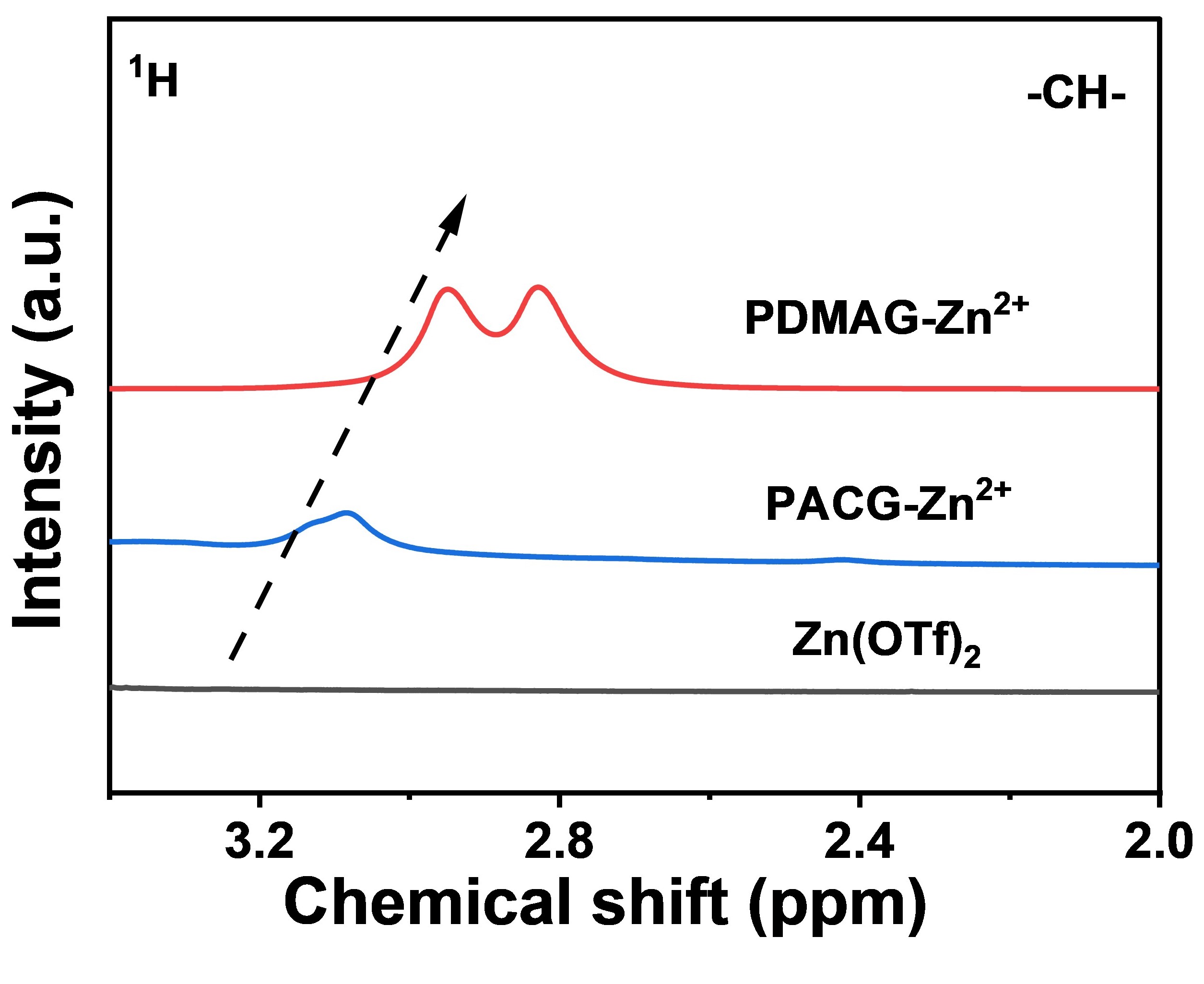


**Fig. S9** The ^1^H NMR spectra of -CH- in 2 M Zn(OTf)_2_, PACG-Zn^2+^, and PDMAG-Zn^2+^


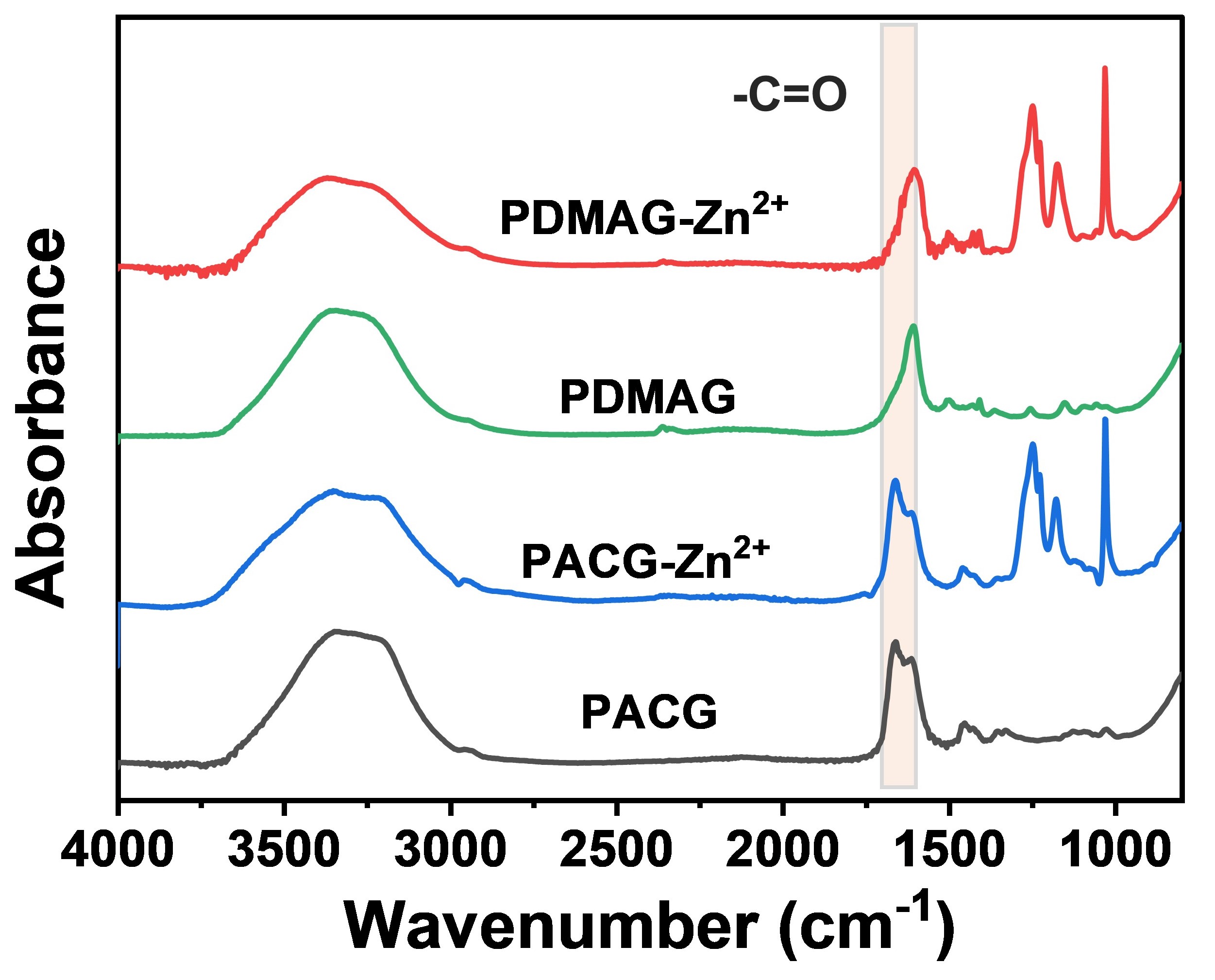


**Fig. S10** FTIR spectra of PACG, PACG-Zn^2+^, PDMAG, and PDMAG-Zn^2+^

**
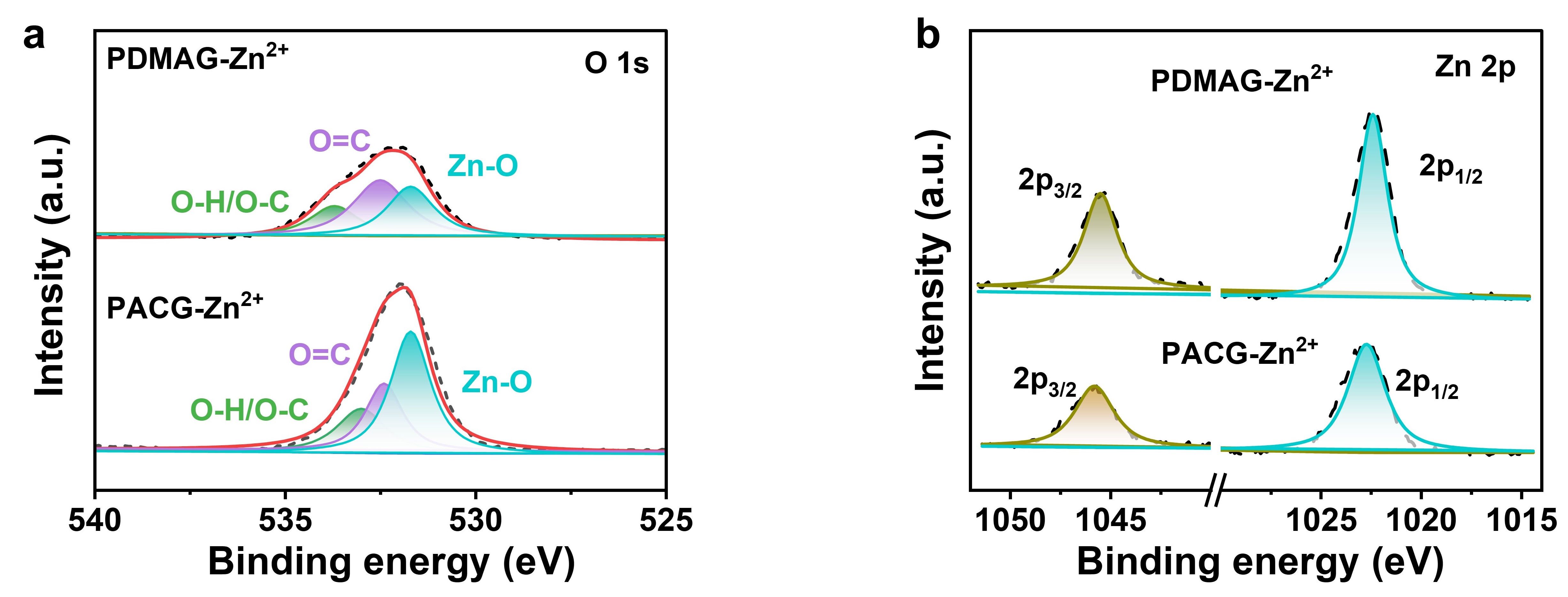
**

**Fig. S11** XPS spectra of **a** O 1s and **b** Zn 2p for PACG-Zn^2+^ and PDMAG-Zn^2+^


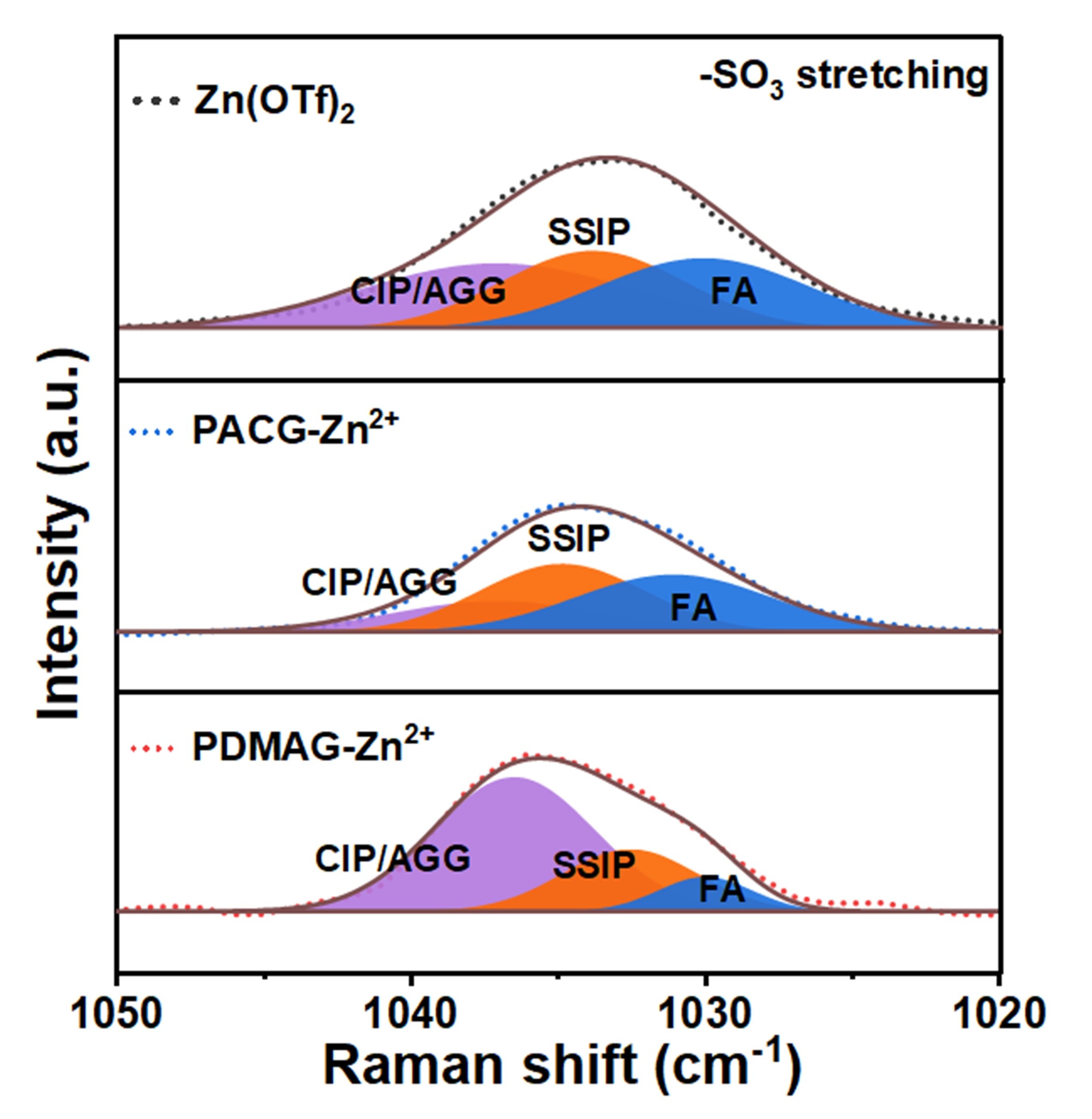


**Fig. S12** Raman spectra for -SO_3_ stretching with 2 M Zn(OTf)_2_, PACG-Zn^2+^, and PDMAG-Zn^2+^


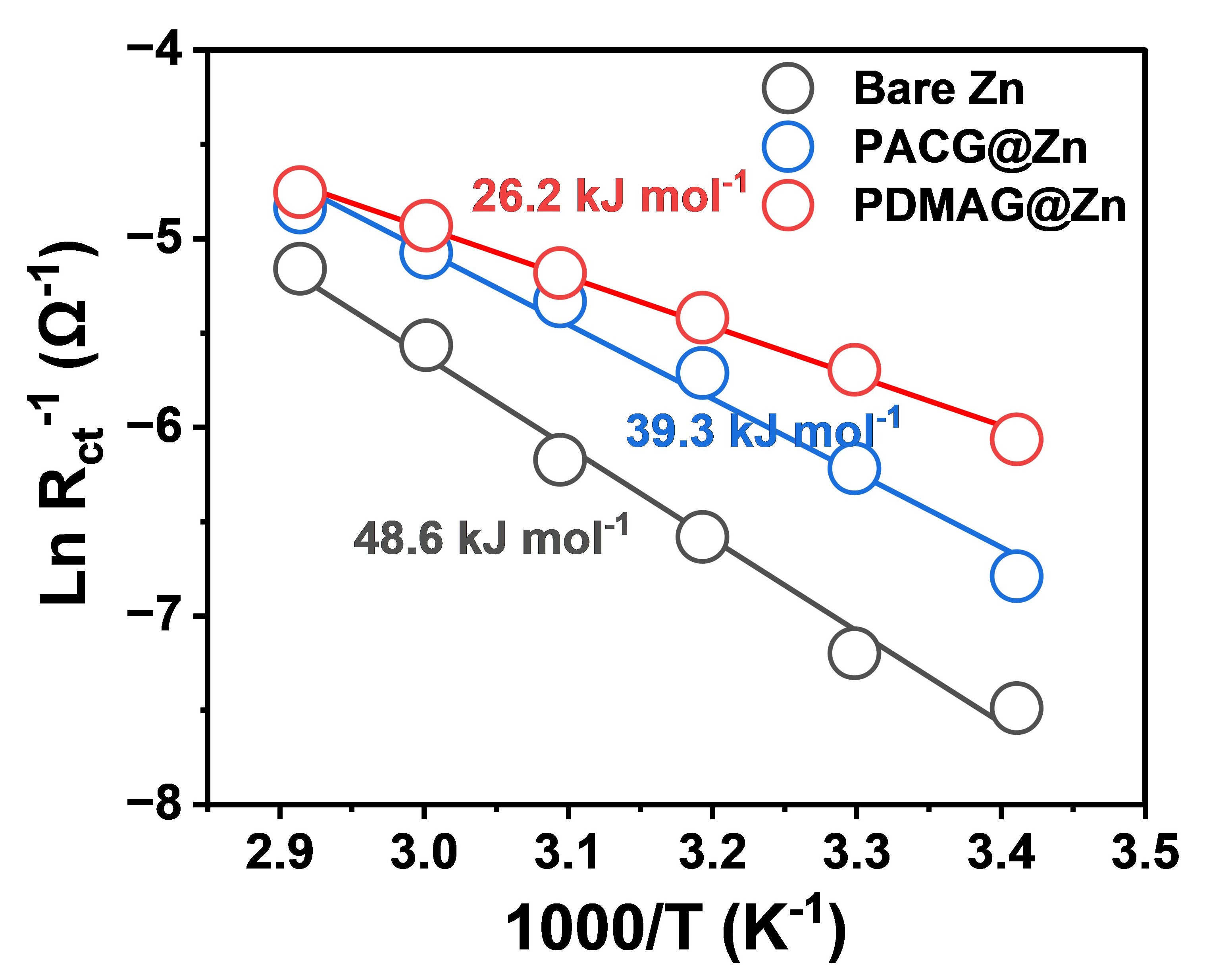


**Fig. S13** Arrhenius curves and the activation energies (*E_a_*)


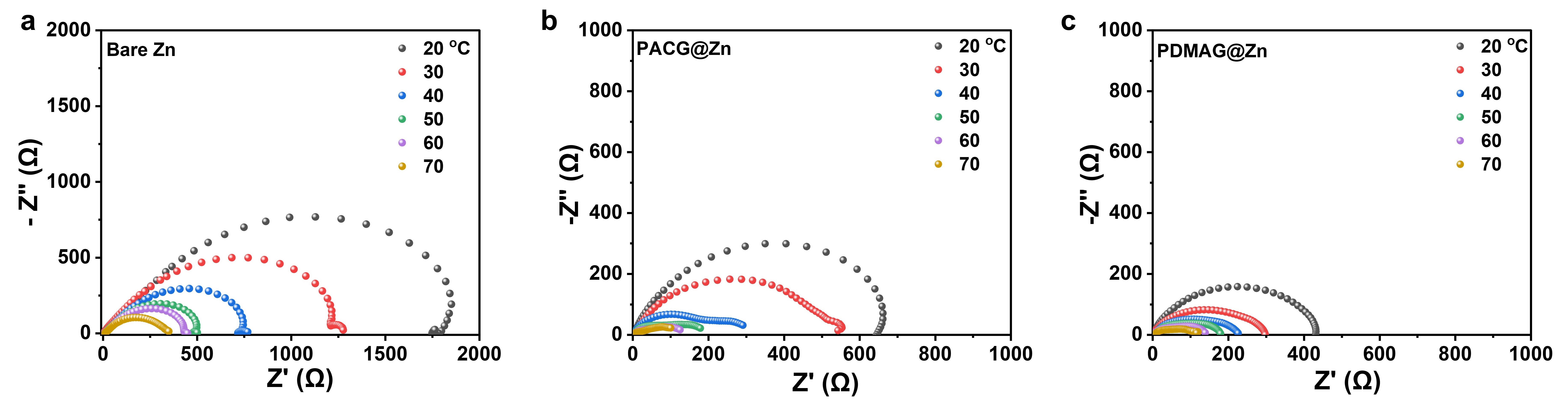


**Fig. S14** The EIS curves of the symmetrical Zn batteries with **a** bare Zn, **b** PACG@Zn, and **c** PDMAG@Zn under the temperatures from 20 to 70 ^o^C


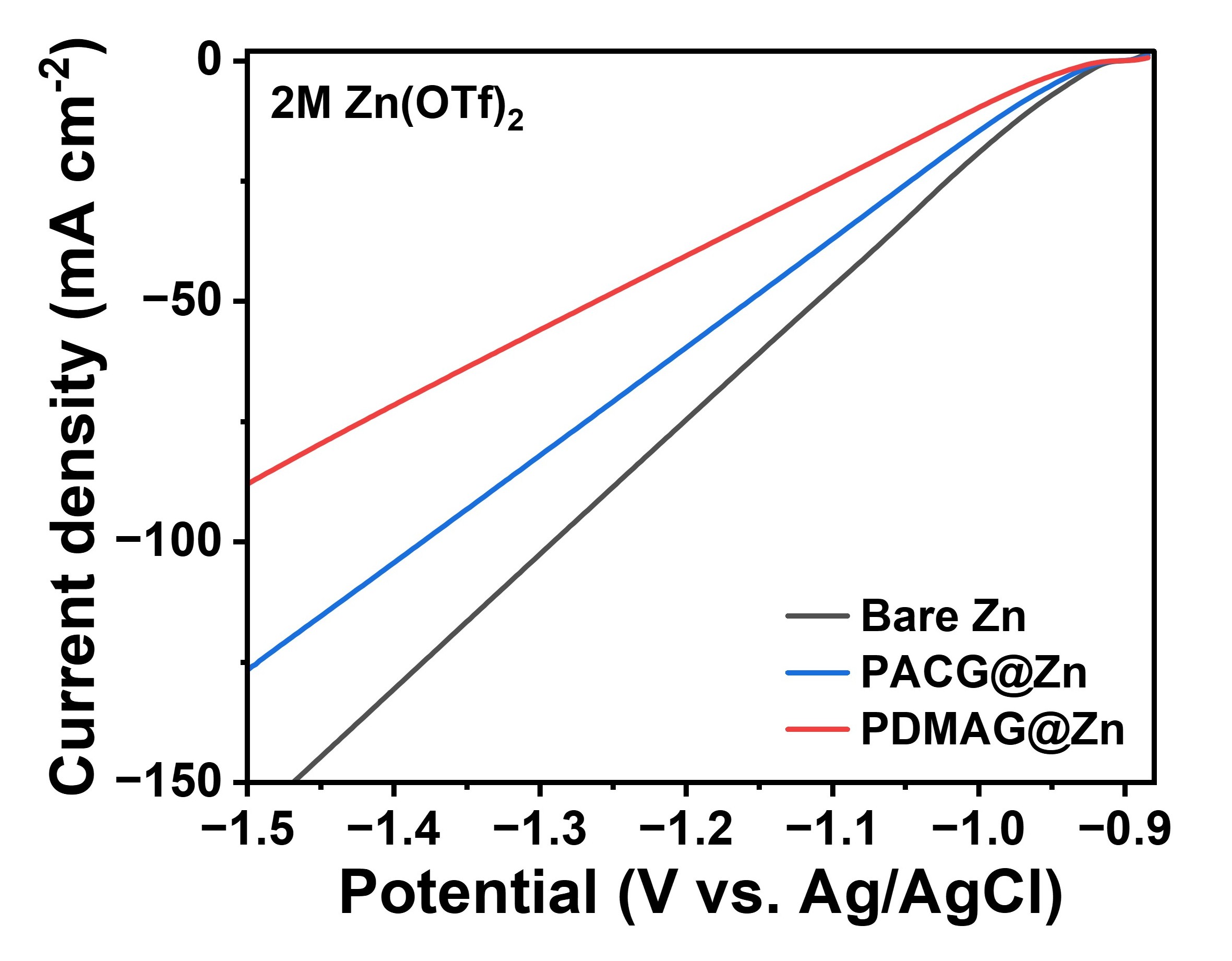


**Fig. S15** LSV curves of bare Zn, PACG@Zn, and PDMAG@Zn in 2 M Zn(OTf)_2_ electrolyte


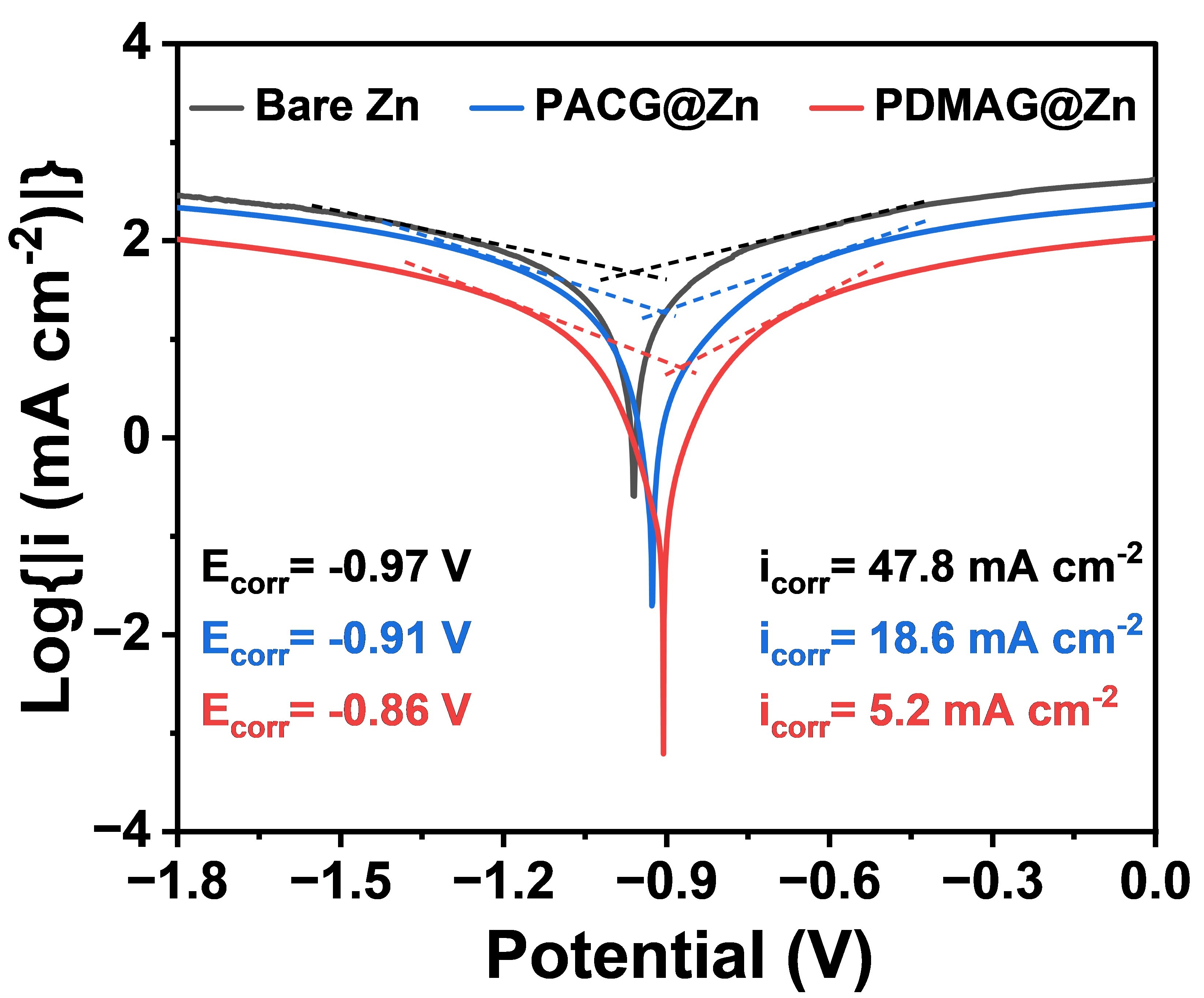


**Fig. S16** Tafel curves of the symmetrical Zn batteries measured with different electrodes


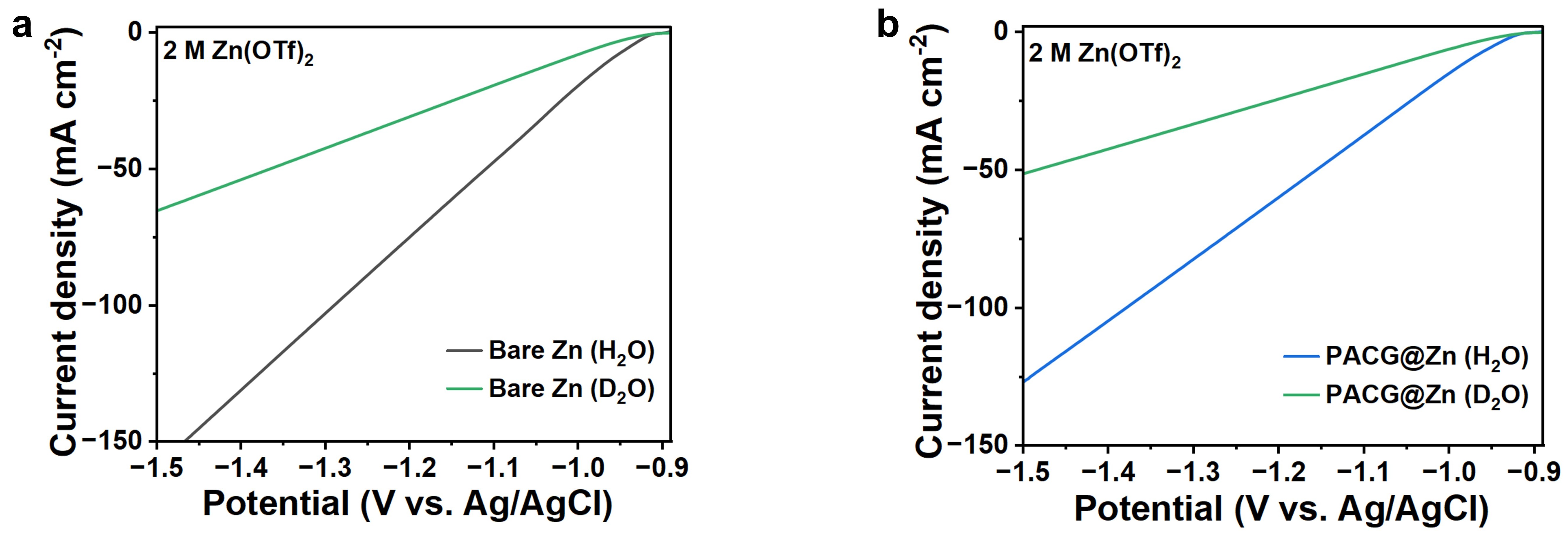


**Fig. S17** The LSV curves of **a** bare Zn and **b** PACG@Zn in 2 M Zn(OTf)_2_ (H_2_O and D_2_O) electrolytes

**
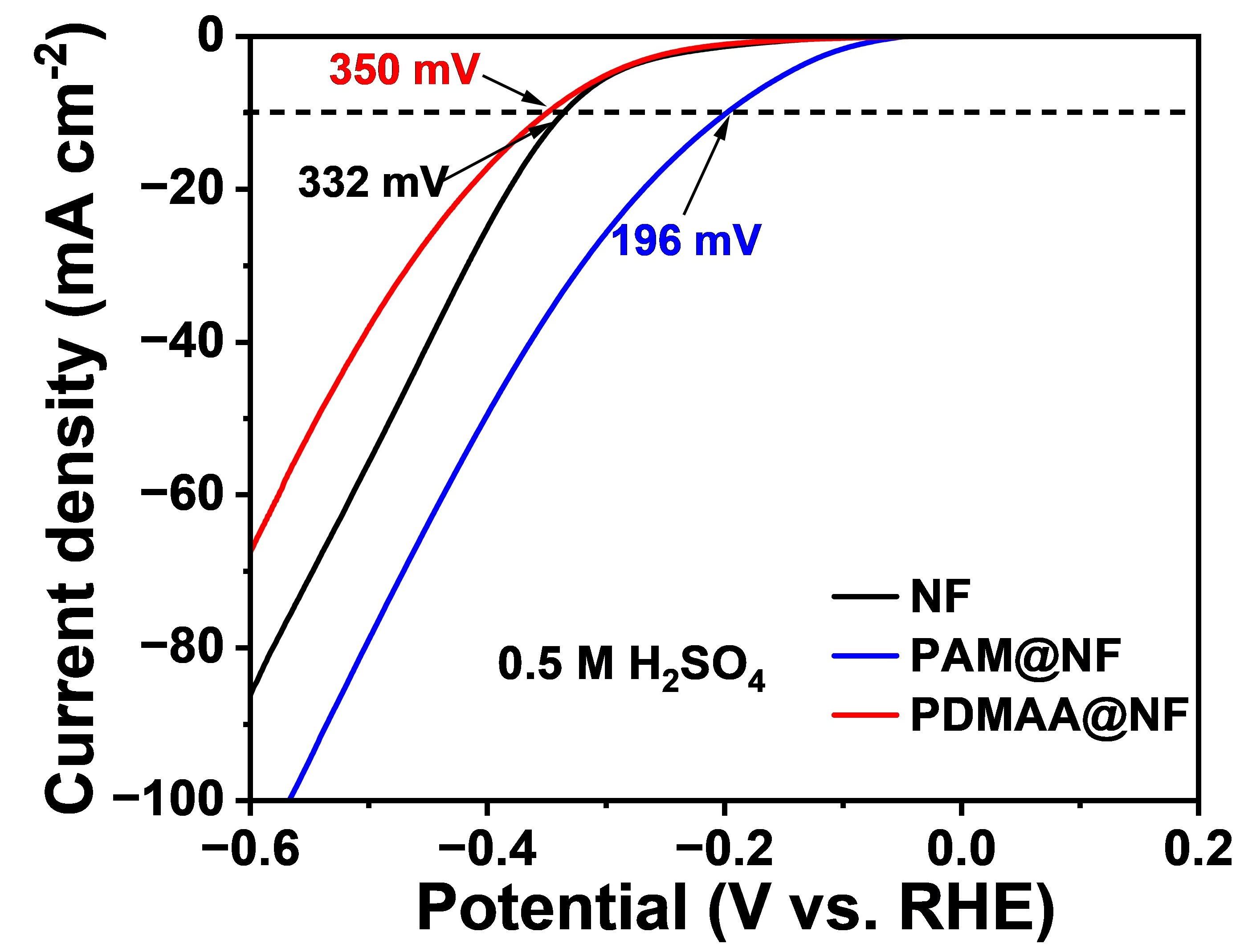
**

**Fig. S18** The HER curves of PAM and PDMAA coated on NF substrates at 0.5 M H_2_SO_4_ electrolyte


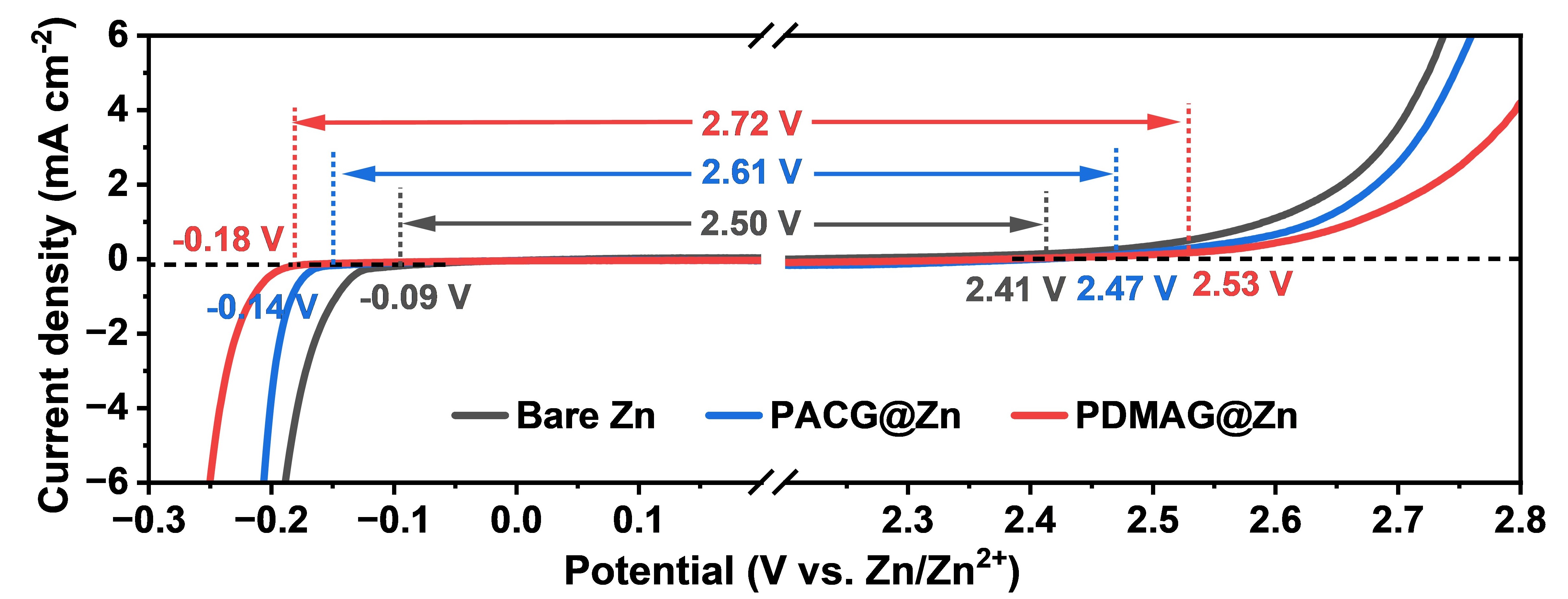


**Fig. S19** The ESW curves of bare Zn, PACG@Zn and PDMAG@Zn.


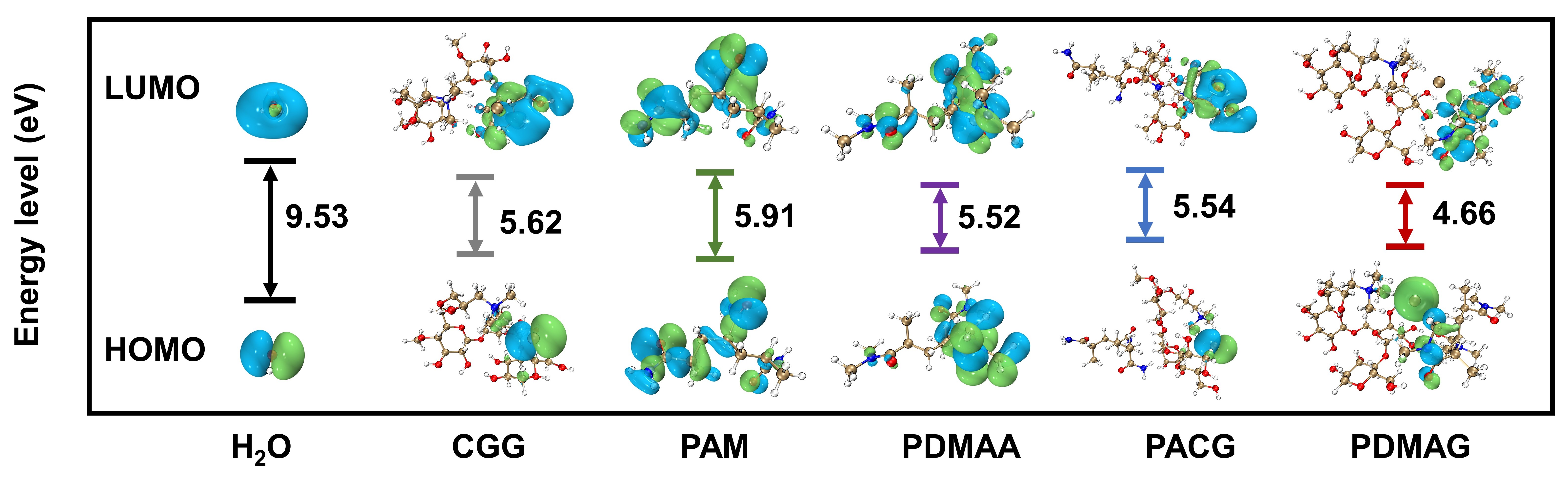


**Fig. S20** The HOMO-LUMO energy levels for H_2_O, CGG, PAM, PDMAA, PACG and PDMAG


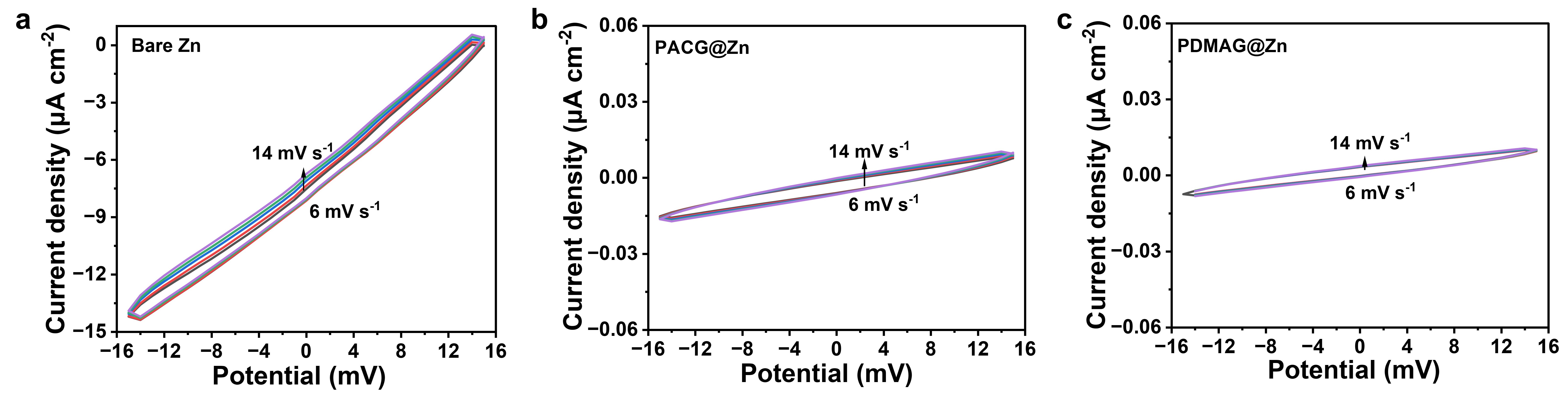


**Fig. S21** The CV curves of symmetrical Zn batteries with **a** bare Zn, **b** PACG@Zn, and **c** PDMAG@Zn under various scanning rates

**
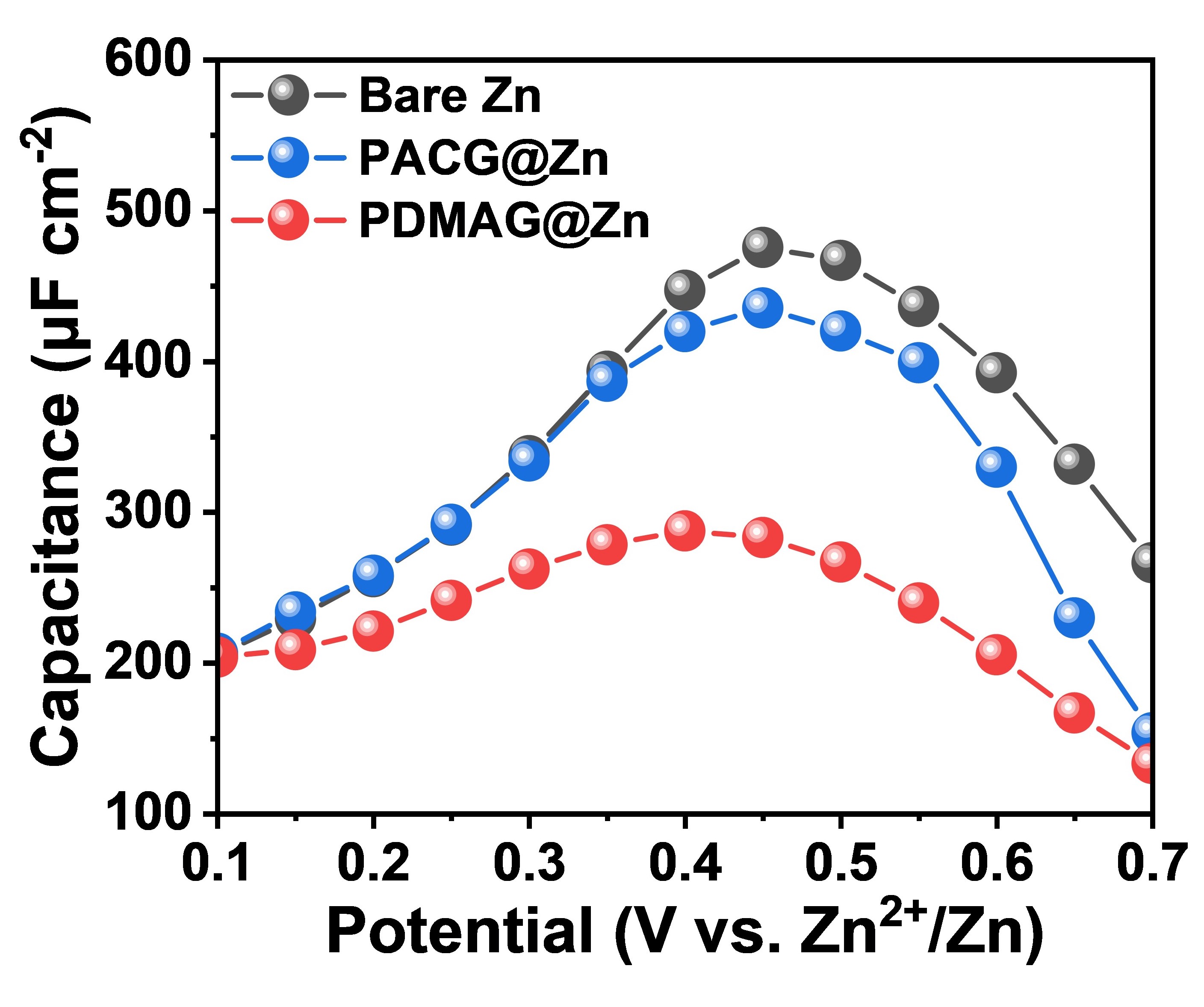
**

**Fig. S22** Differential capacitance curves for bare Zn, PACG@Zn, and PDMAG@Zn

**
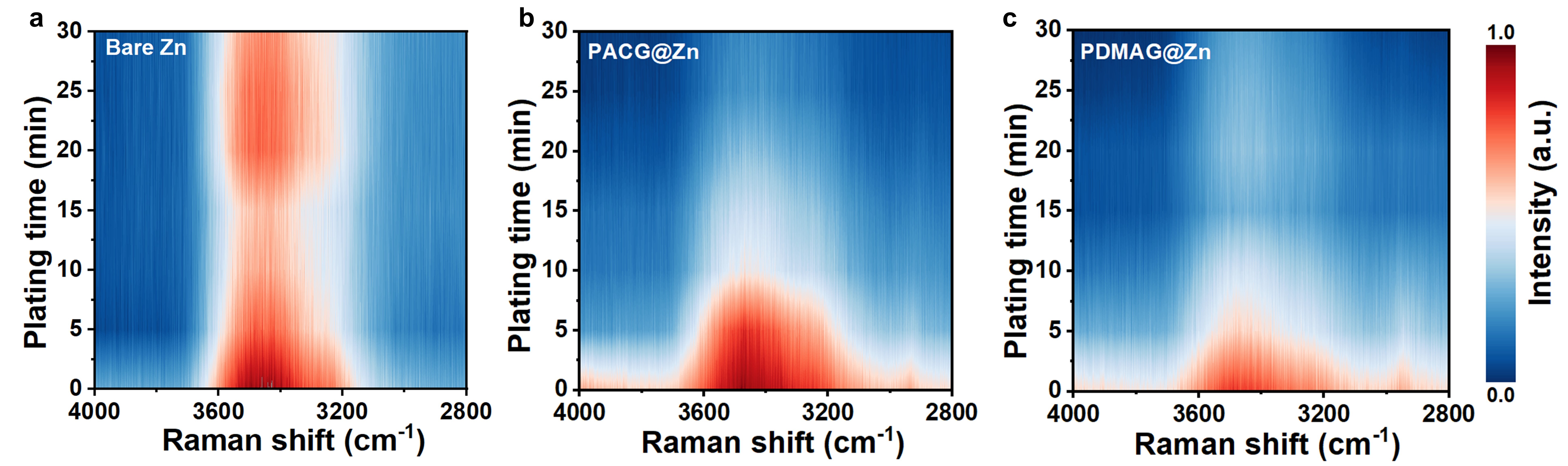
**

**Fig. S23** Raman spectra of H_2_O signals at the electrode/electrolyte interface with different electrodes


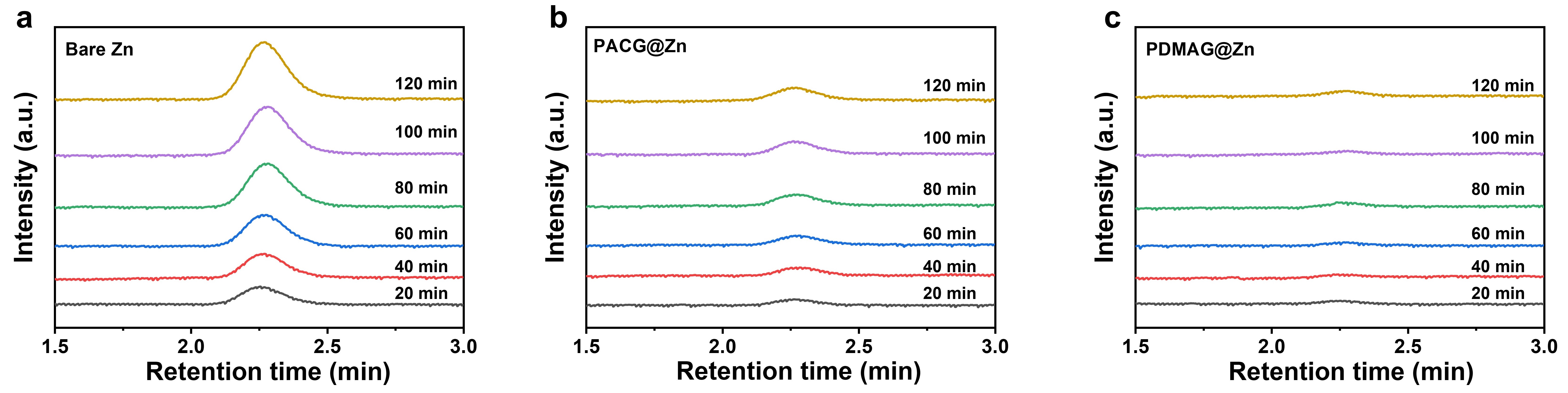


**Fig. S24** In-situ EC-GC curves during Zn plating at 10 mA cm^-2^ with **a** bare Zn, **b** PACG@Zn, and **c** PDMAG@Zn


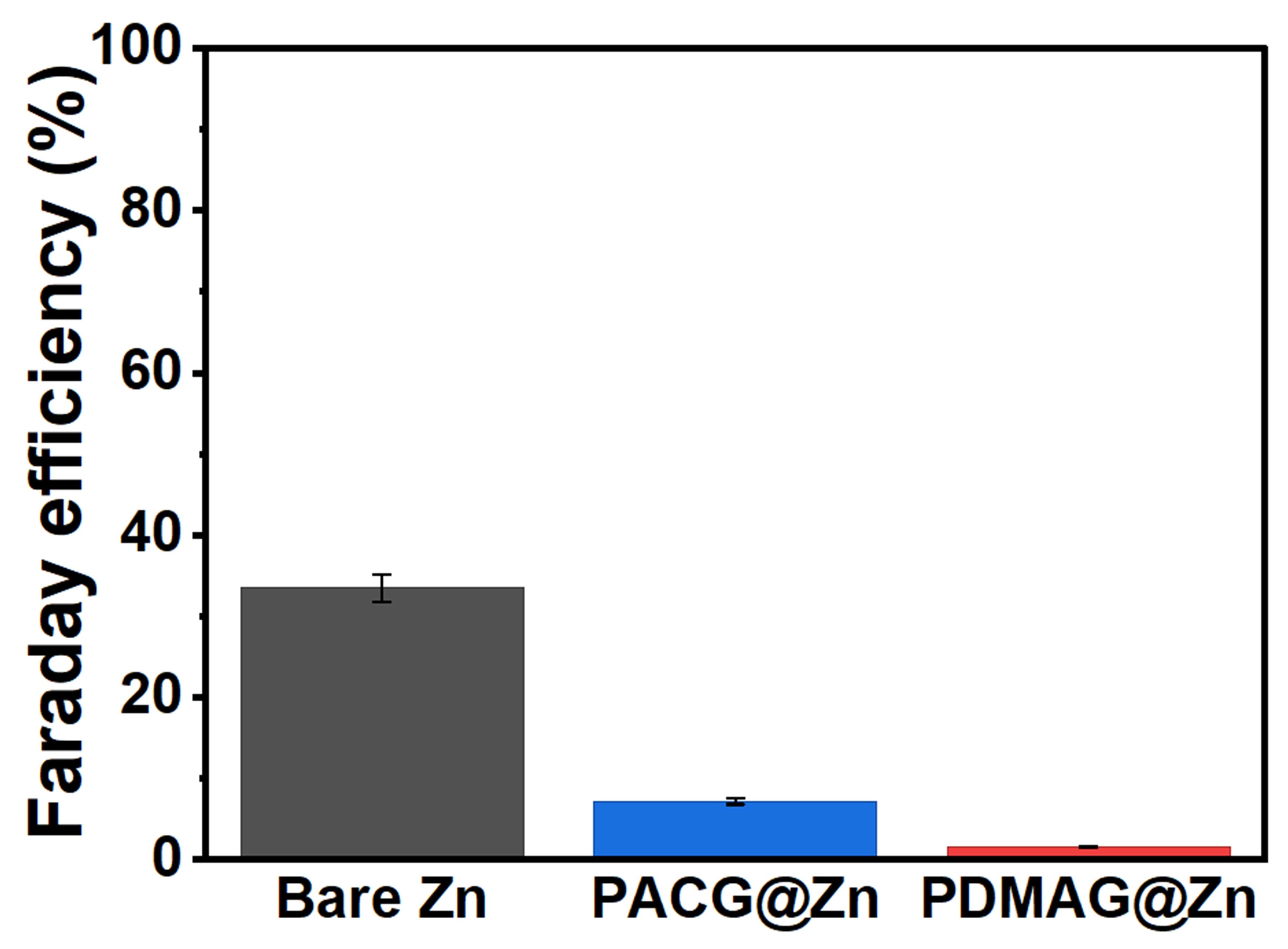


**Fig. S25** Faraday efficiency of different Zn electrodes during Zn plating at 10 mA cm^-2^


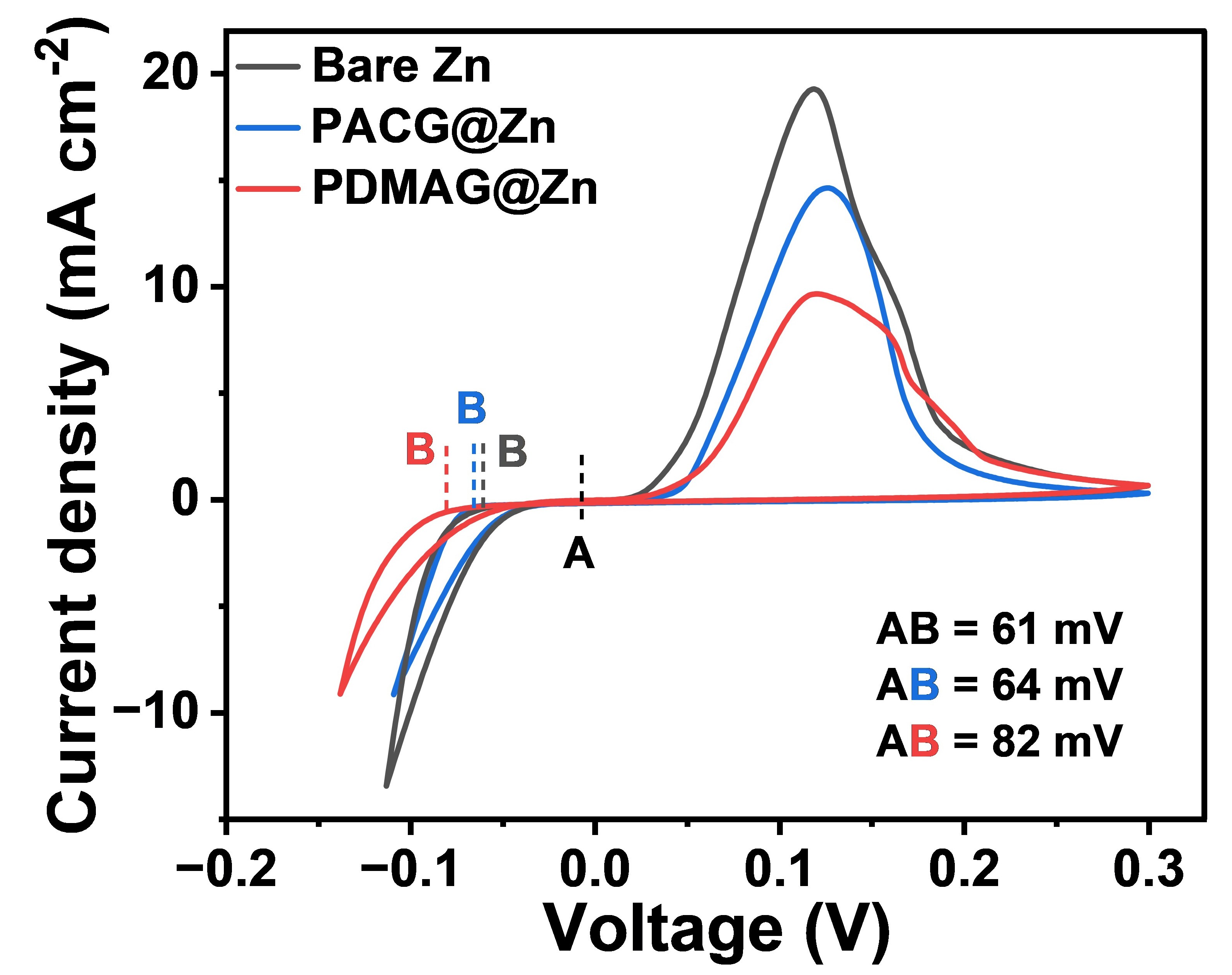


**Fig. S26** CV curves for Zn nucleation on different electrodes


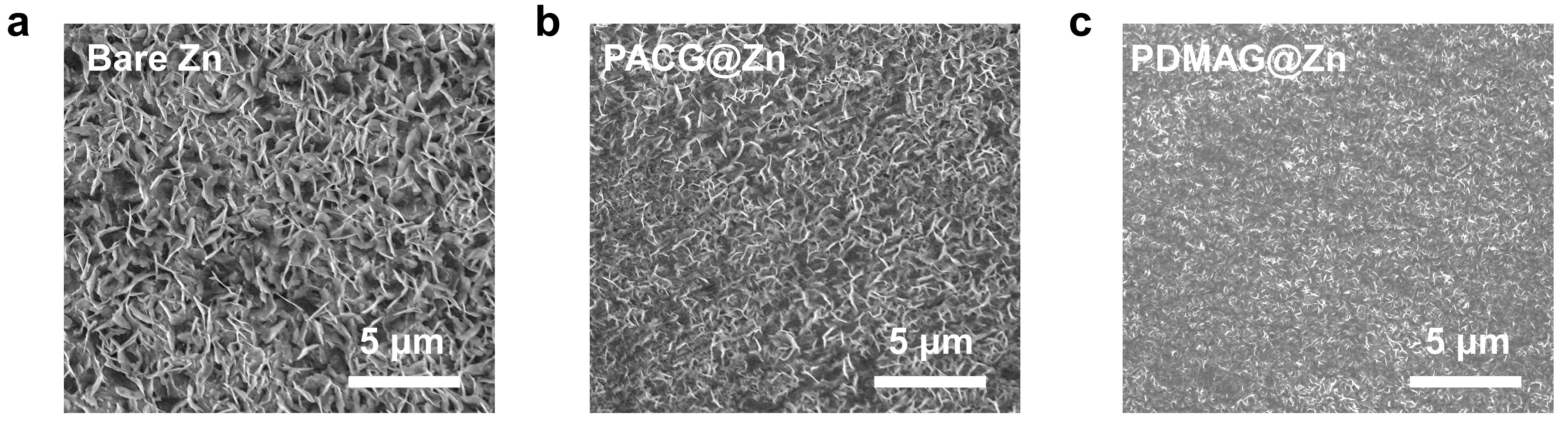


**Fig. S27** SEM images for Zn nucleation on different electrodes


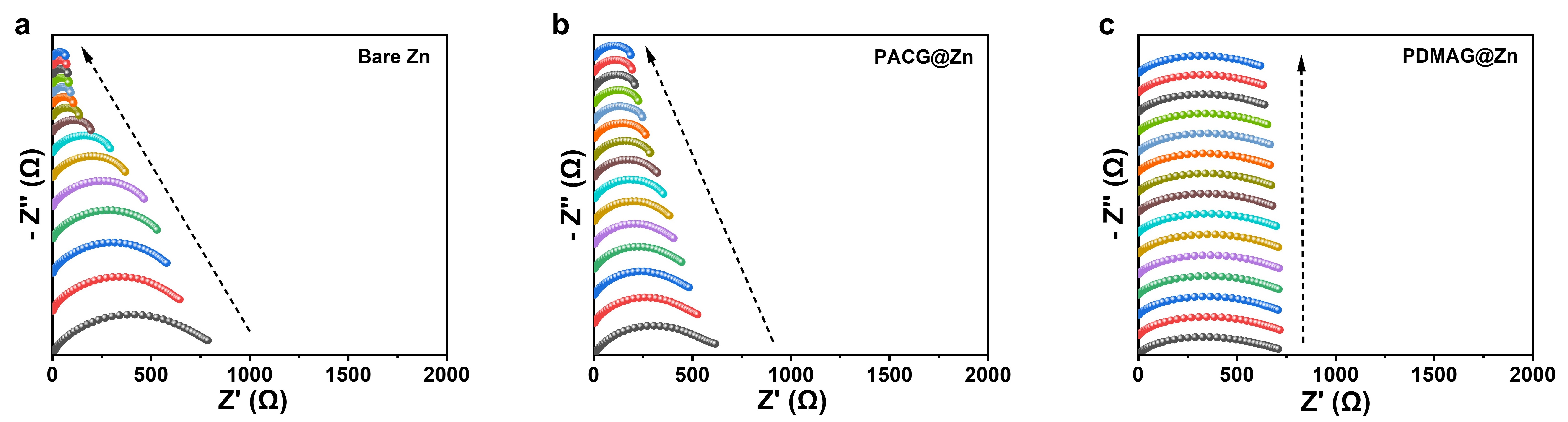


**Fig. S28** The in situ EIS curves of symmetrical Zn batteries with different electrodes


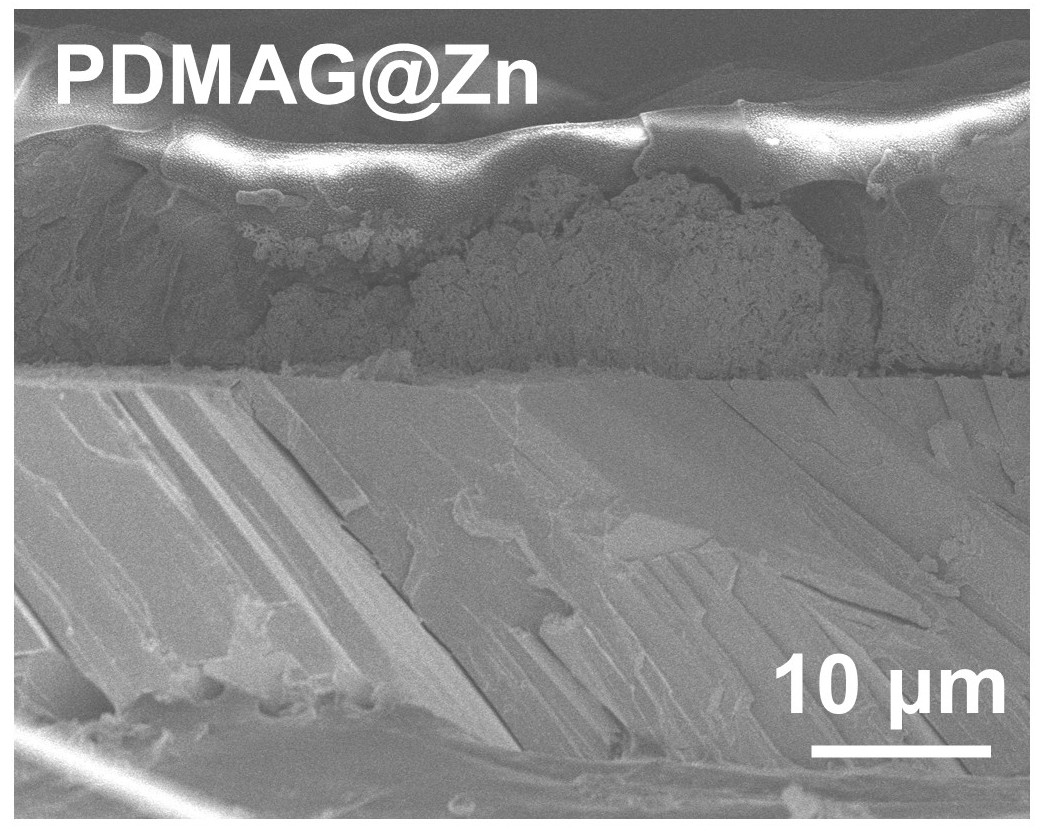


**Fig. S29** SEM images of the zinc anode with PDMAG layer after deposition under 1 mA cm^-2^, 10 mA h cm^-2^


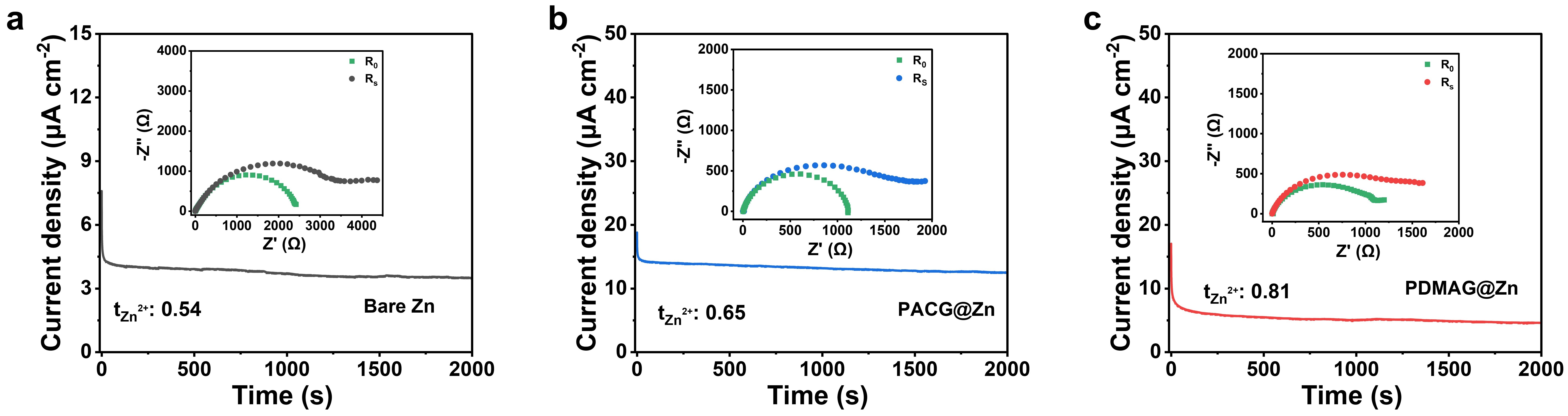


**Fig. S30** The Zn^2+^ transference number measurements for different Zn electrodes


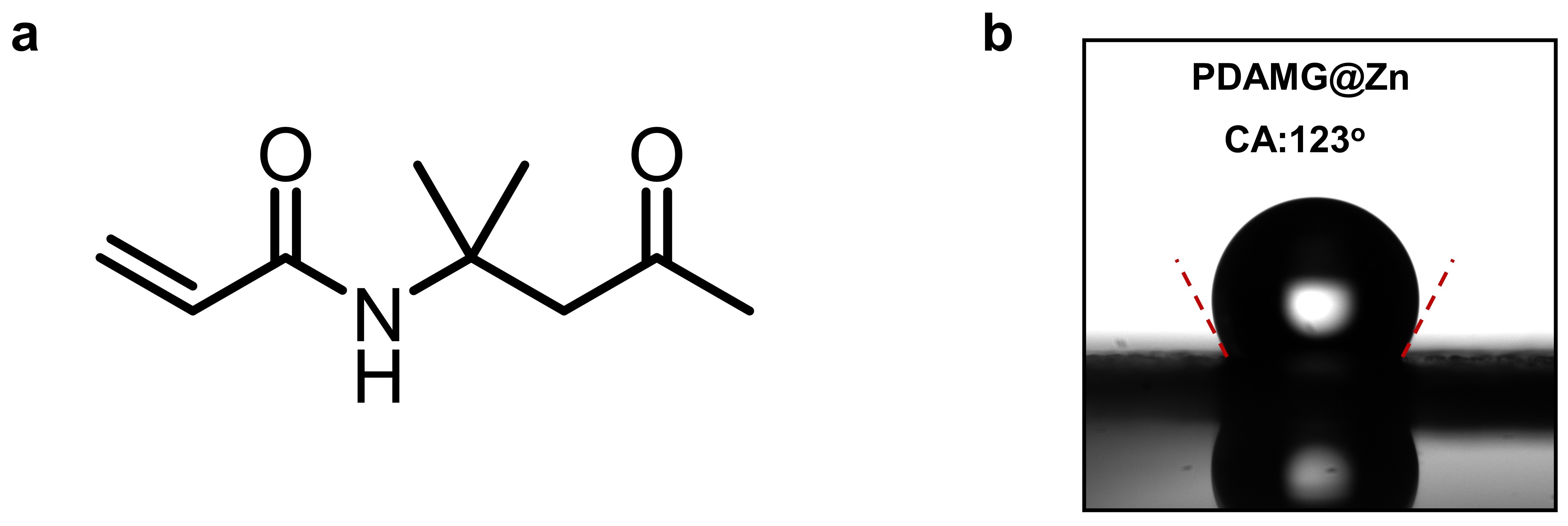


**Fig. S31**  **a** The structure of DAAM monomer and **b** WCA test of PDAMG@Zn


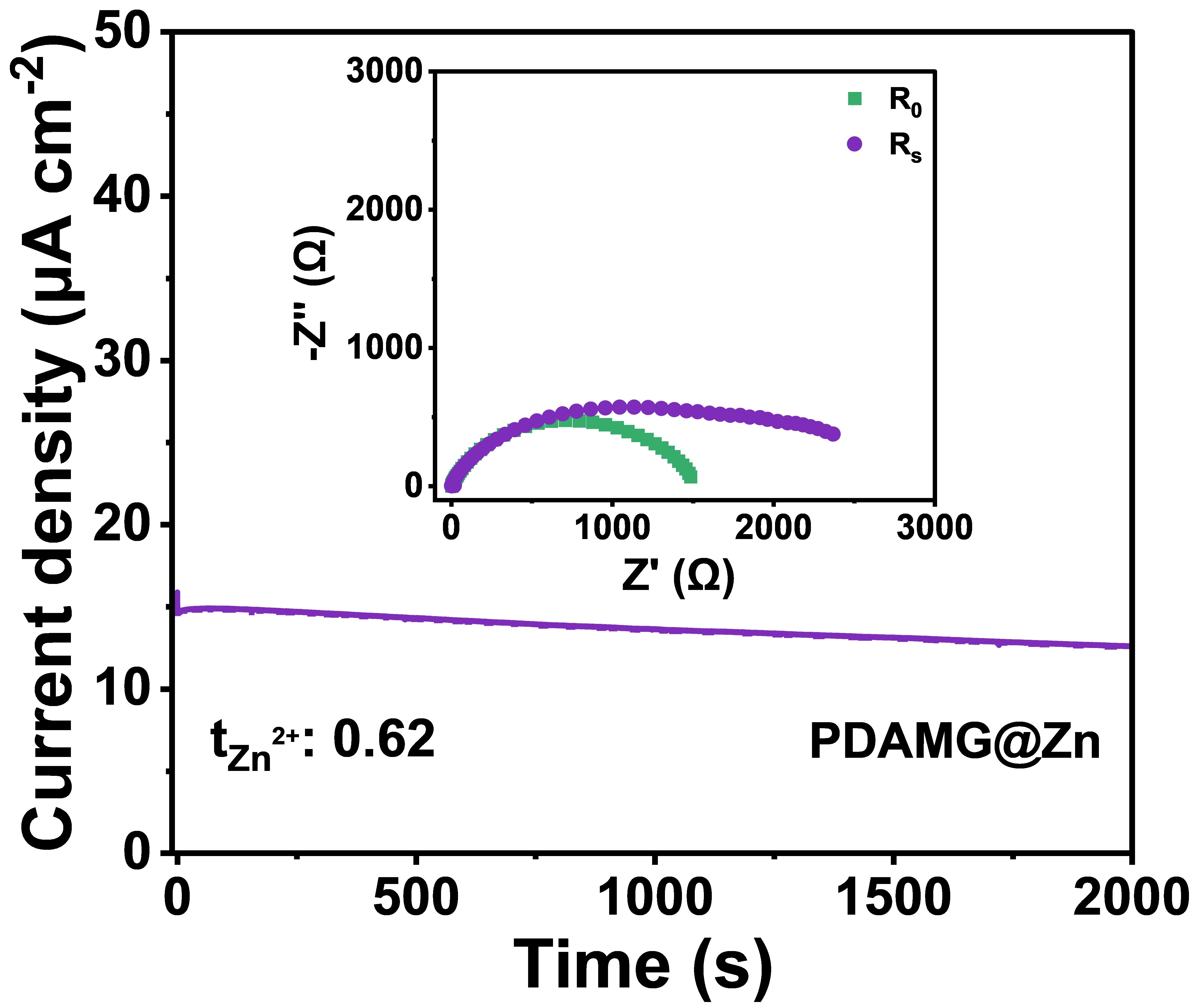


**Fig. S32** The Zn^2+^ transference number measurement for PDAMG@Zn electrode


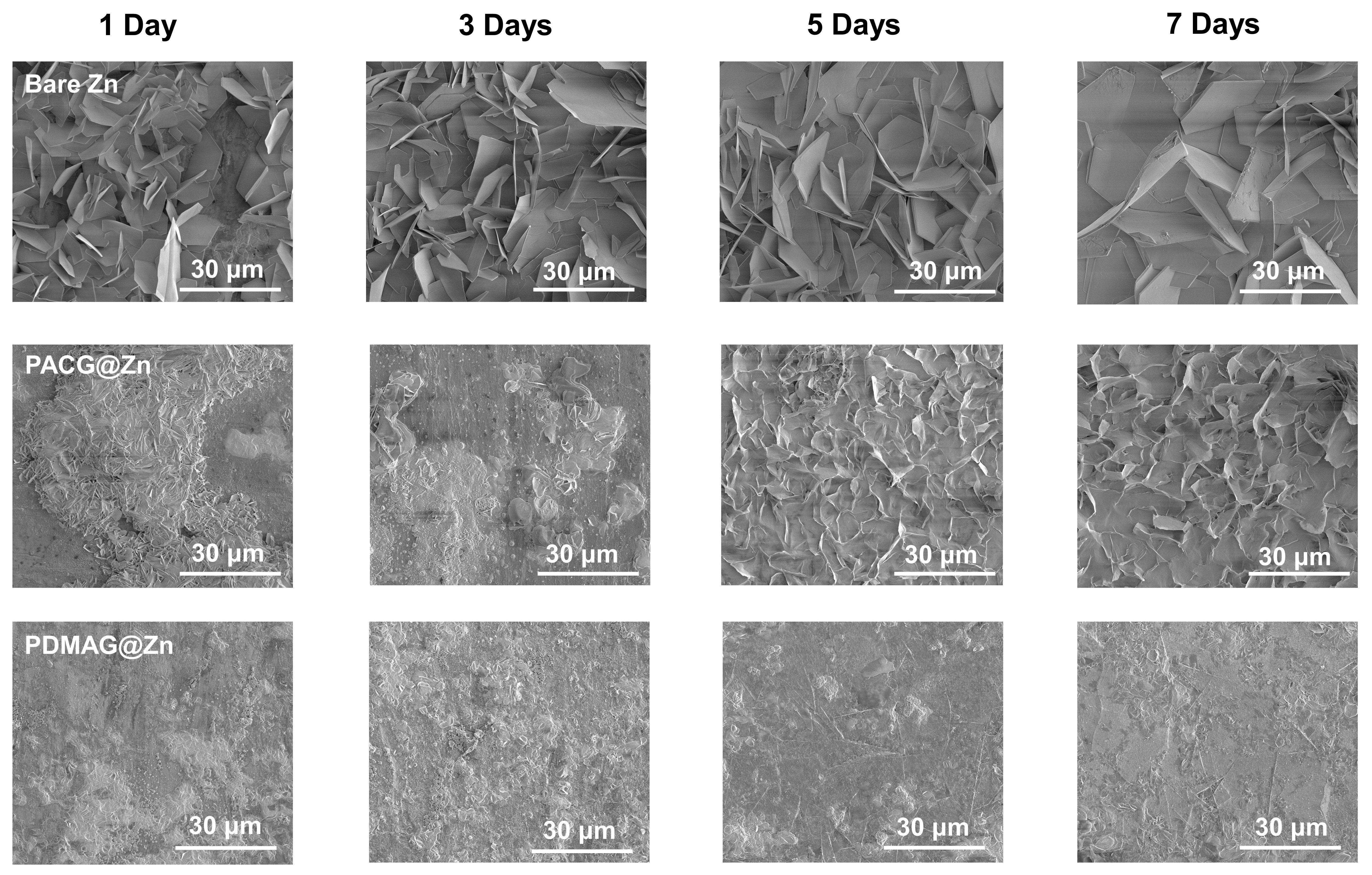


**Fig. S33** The SEM images of bare Zn, PACG@Zn and PDMAG@Zn soaked in 2 M Zn(OTf)_2_ electrolyte for various days


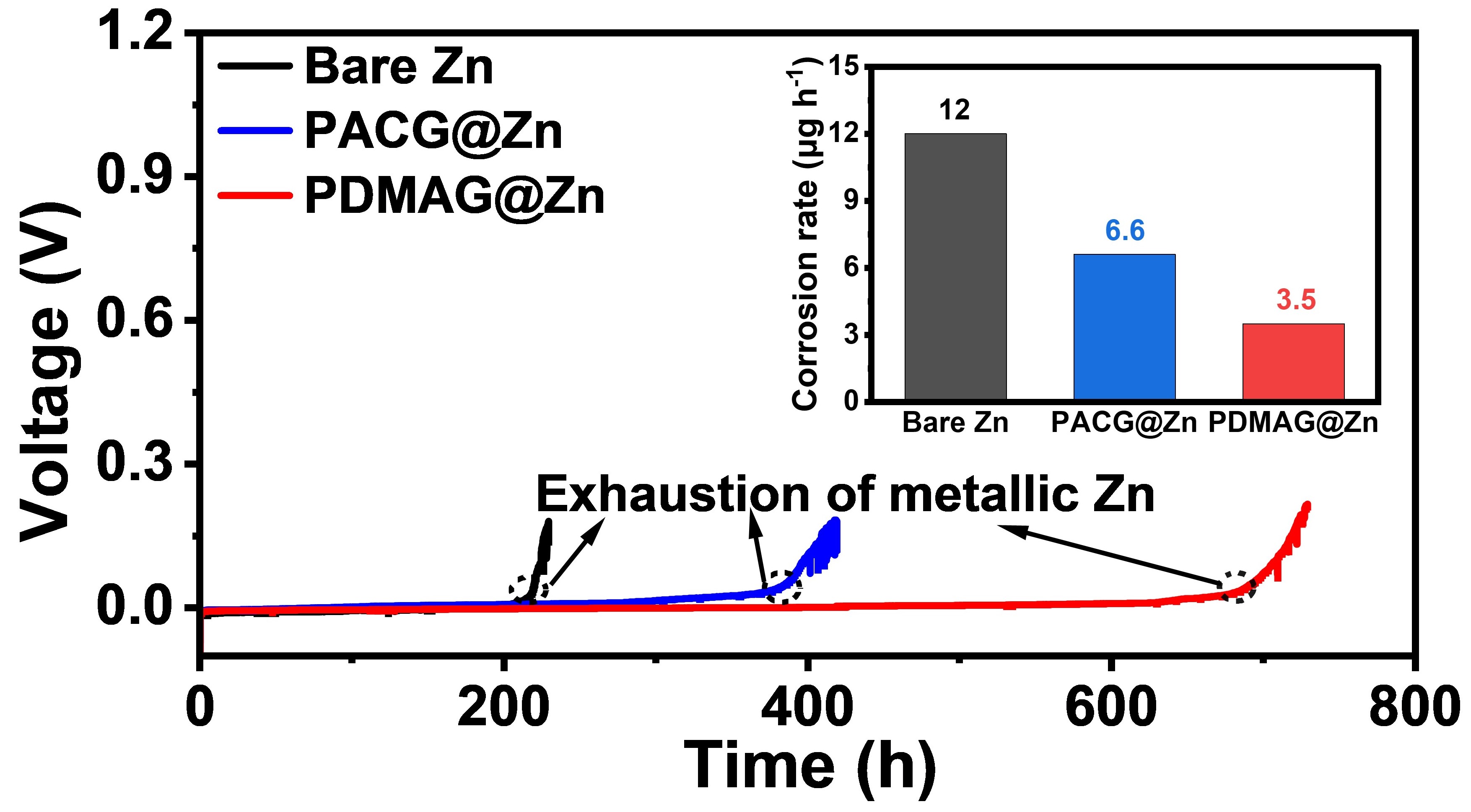


**Fig. S34** Corrosion rate for different Zn electrodes


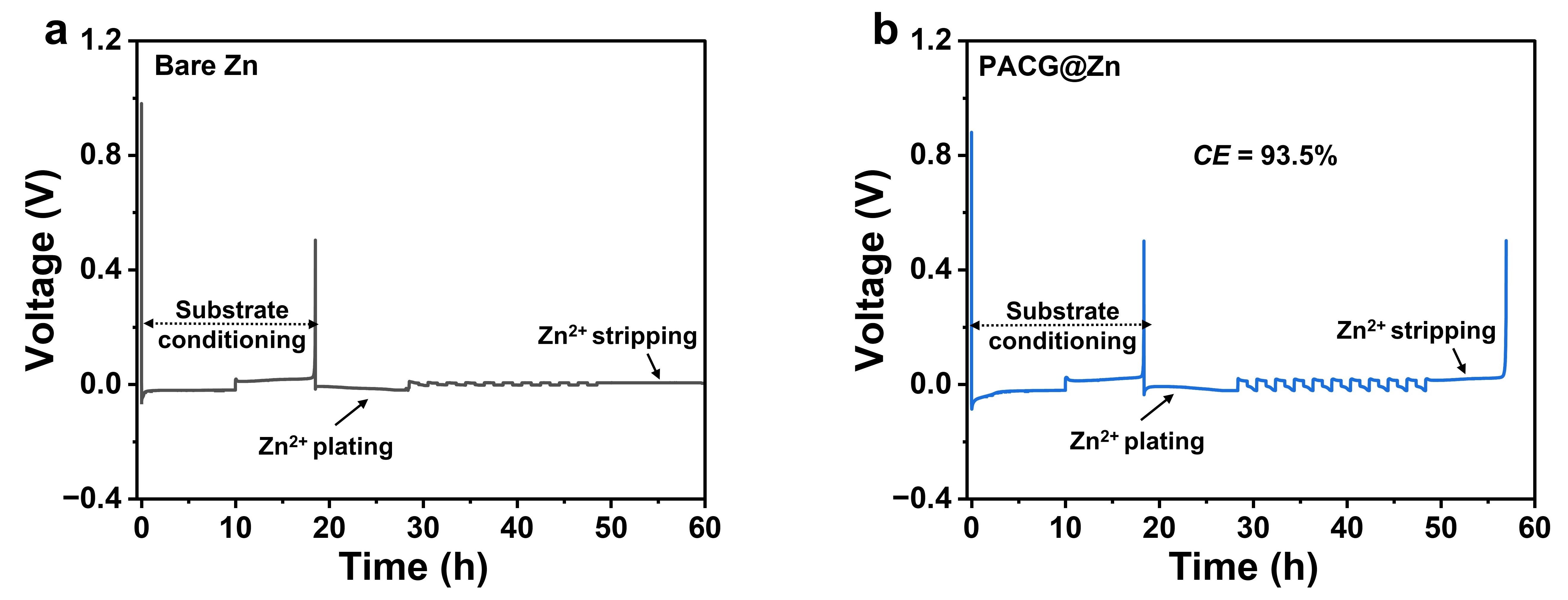


**Fig. S35** The Reservoir-based Zn||Cu batteries for assessing average Zn stripping/plating CE of **a** bare Zn and **b** PACG@Zn


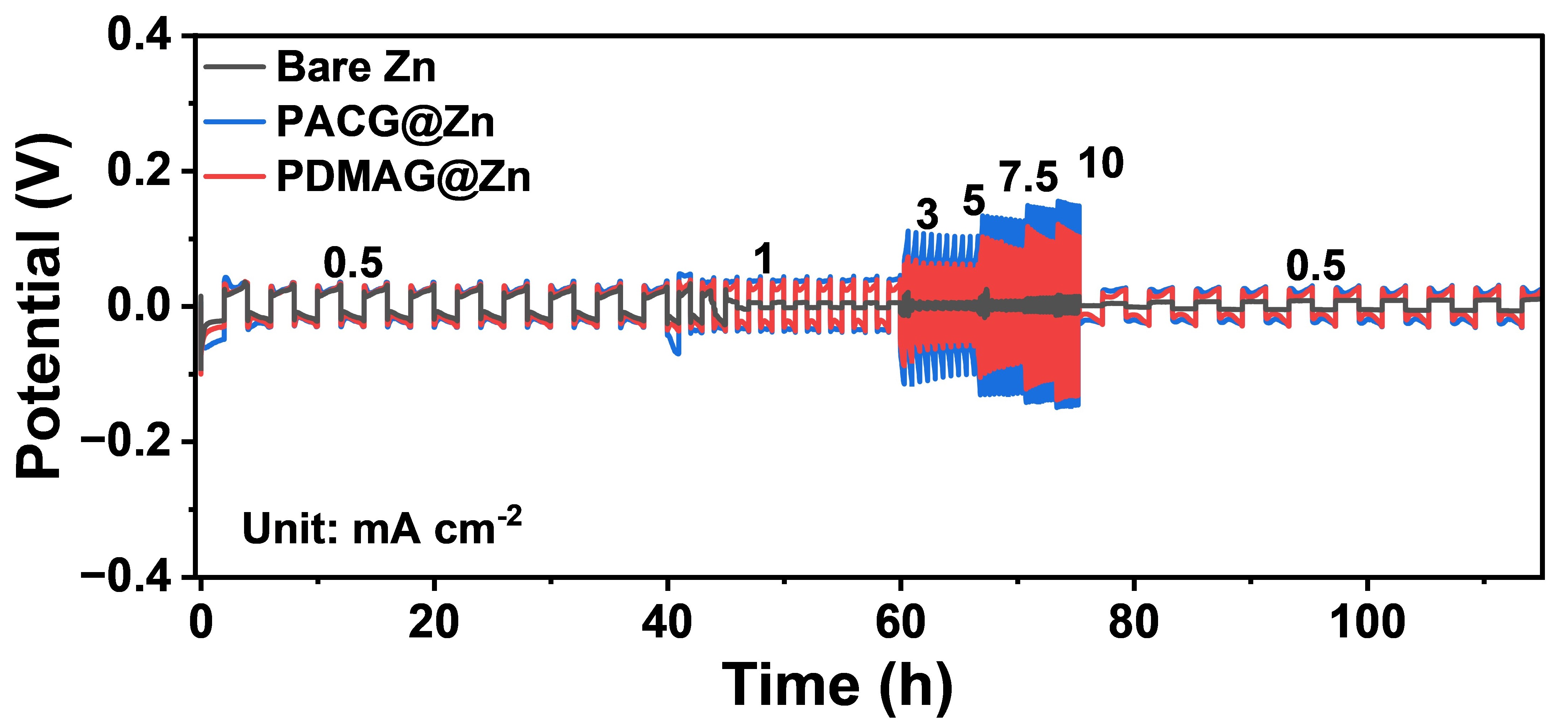


**Fig. S36** The rate performance of symmetrical Zn batteries with different electrodes at various current densities


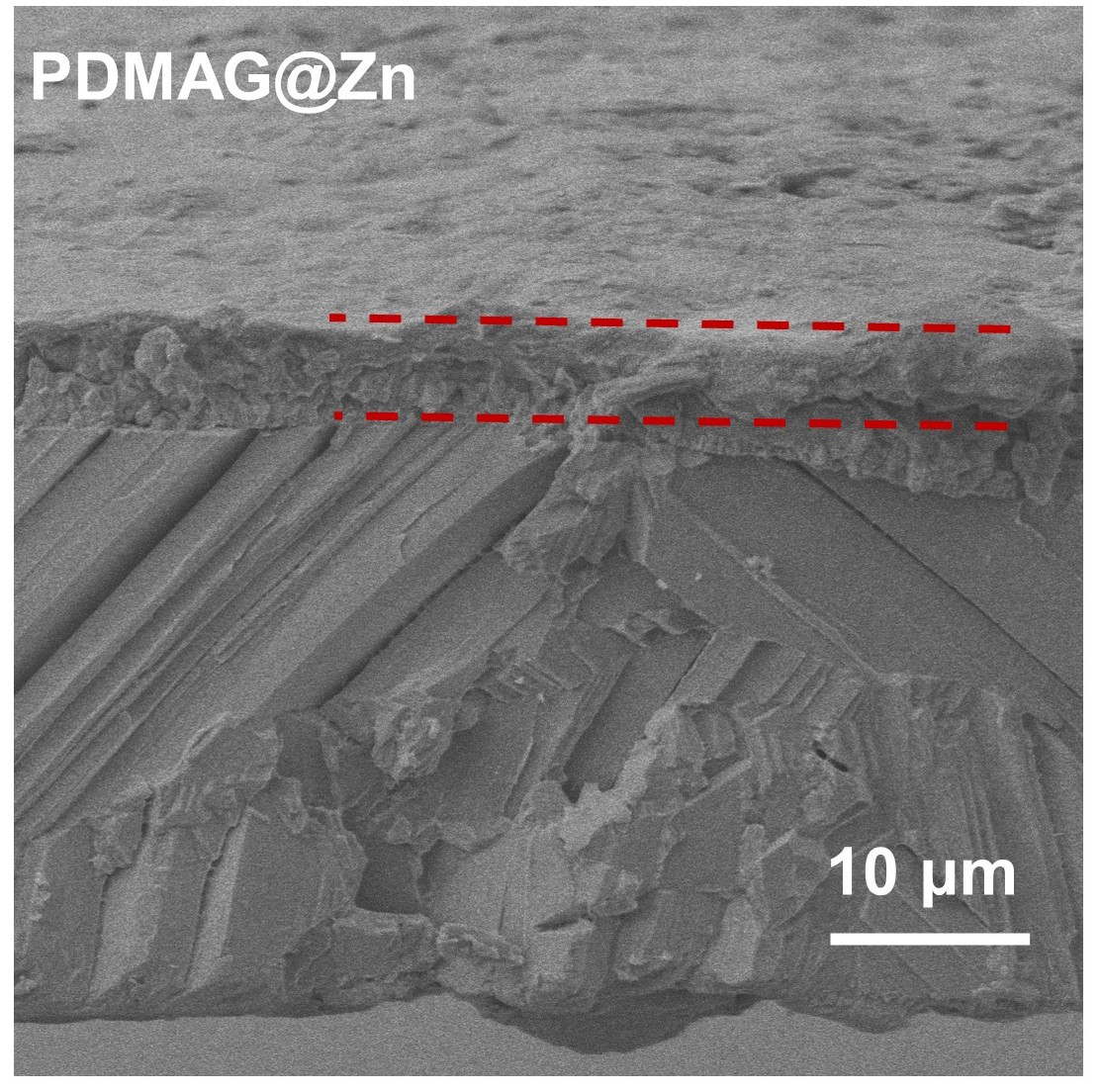


**Fig. R37** Cross-sectional SEM image of PDMAG@Zn after 500 h cycling at 1 mA cm^-2^, 1mA h cm^-2^


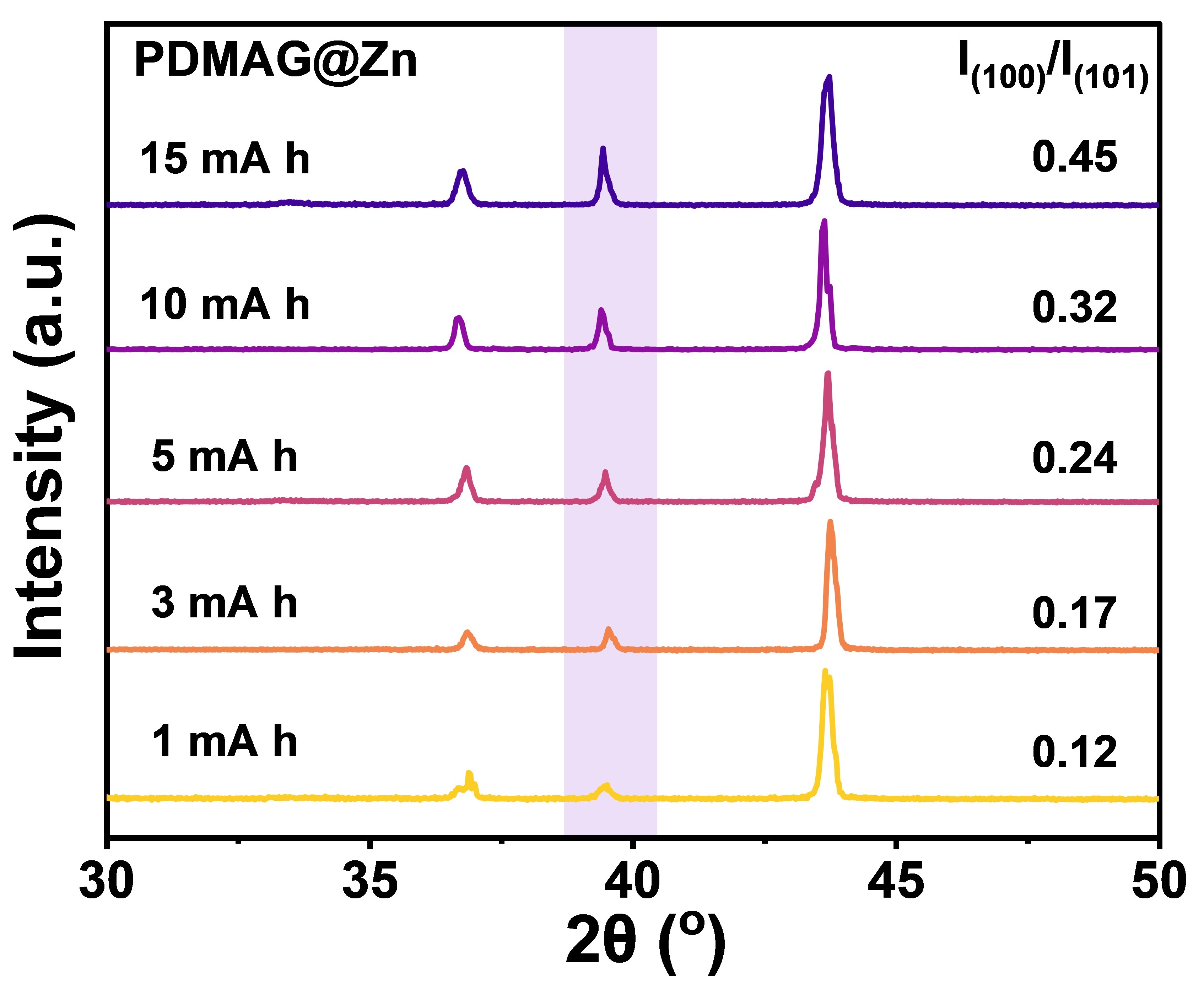


**Fig. S38** XRD spectra of PDMAG@Zn electrodes after plating at 1 mA cm^-2^ with different deposition capacities


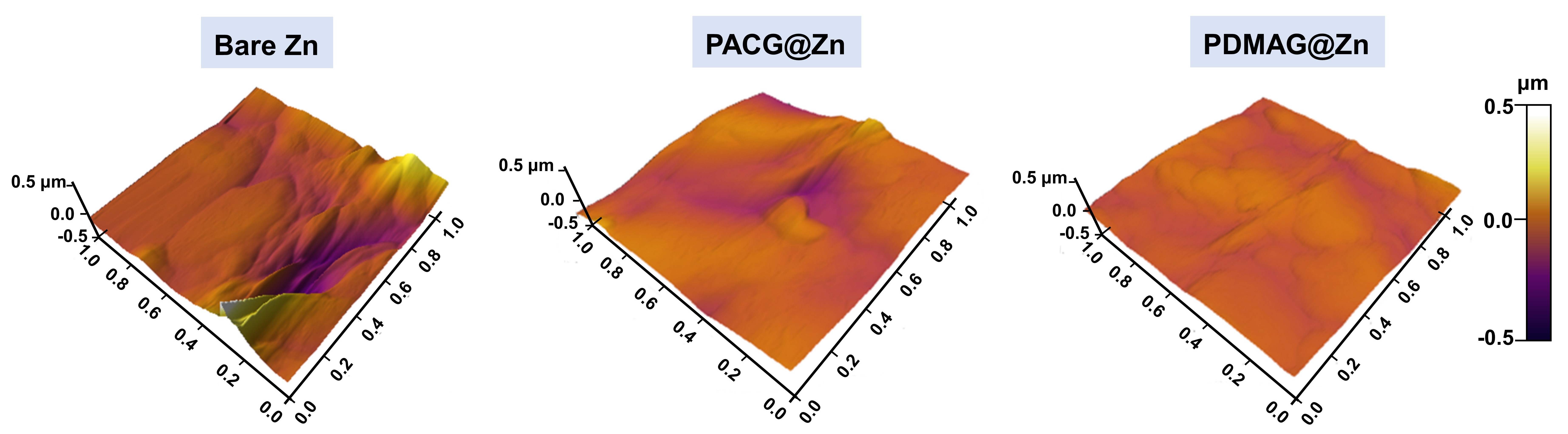


**Fig. S39** AFM images of different electrodes after plating for 1 h at the current density of 10 mA cm^-2^


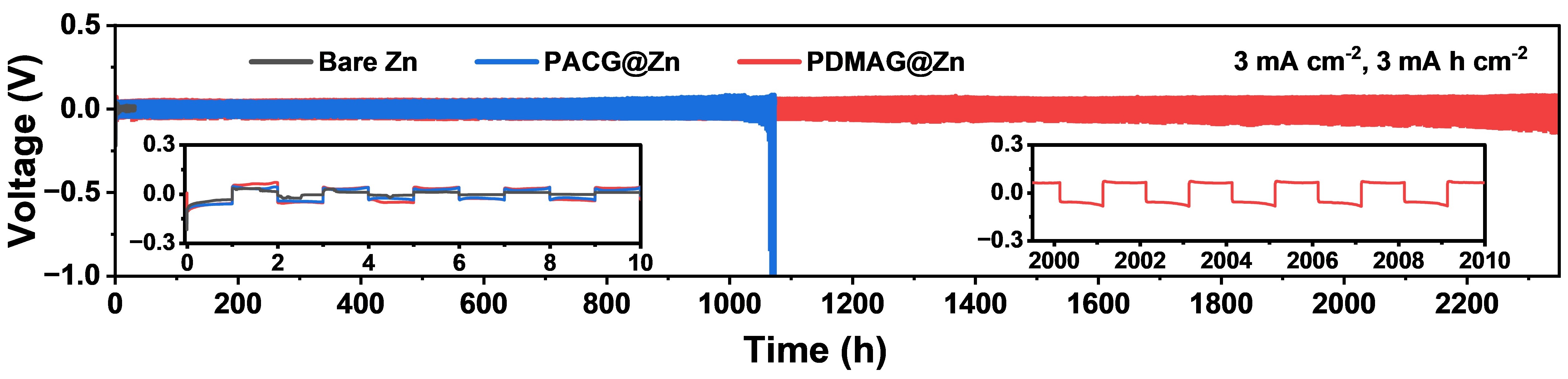


**Fig. S40** The performance of symmetrical Zn batteries with different Zn electrodes at 3 mA cm^-2^, 3 mA h cm^-2^


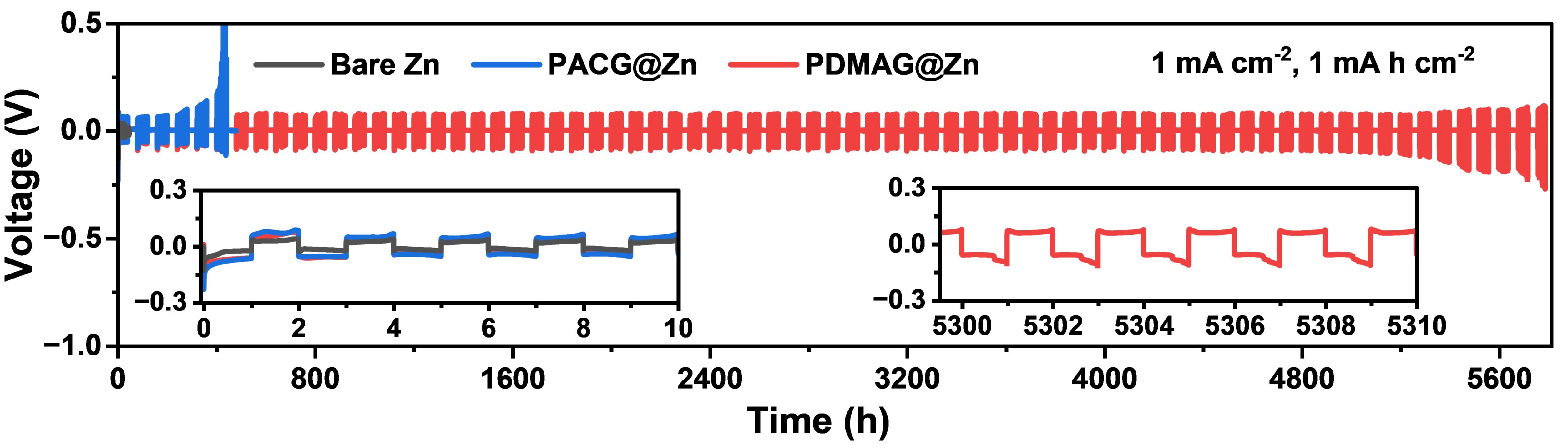


**Fig. S41** The shelving-recovery performance for symmetrical Zn batteries with different Zn electrodes at 1 mA cm^-2^, 1 mA h cm^-2^


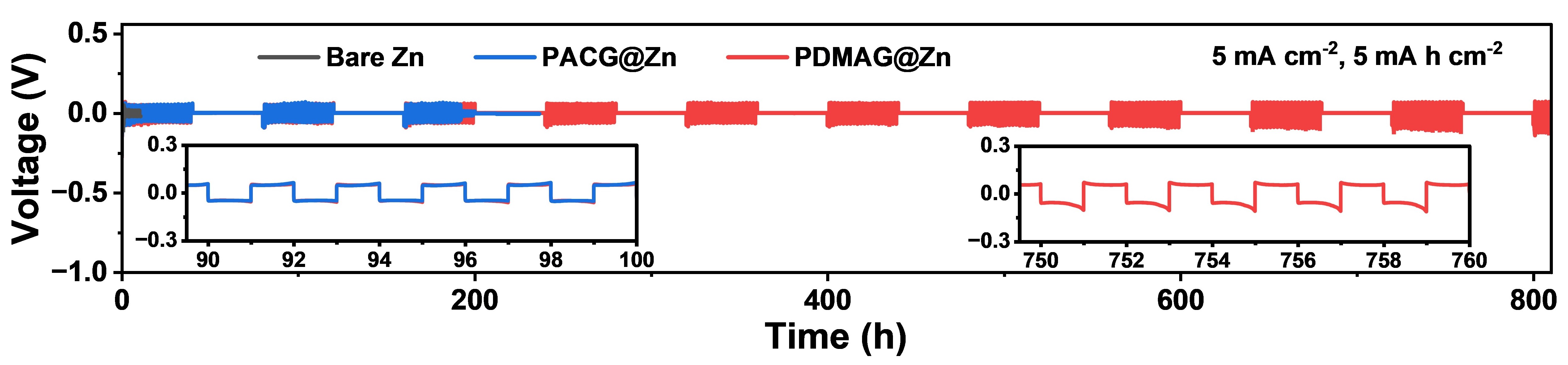


**Fig. S42** The shelving-recovery performance for symmetrical Zn batteries with different Zn electrodes at 5 mA cm^-2^, 5 mA h cm^-2^


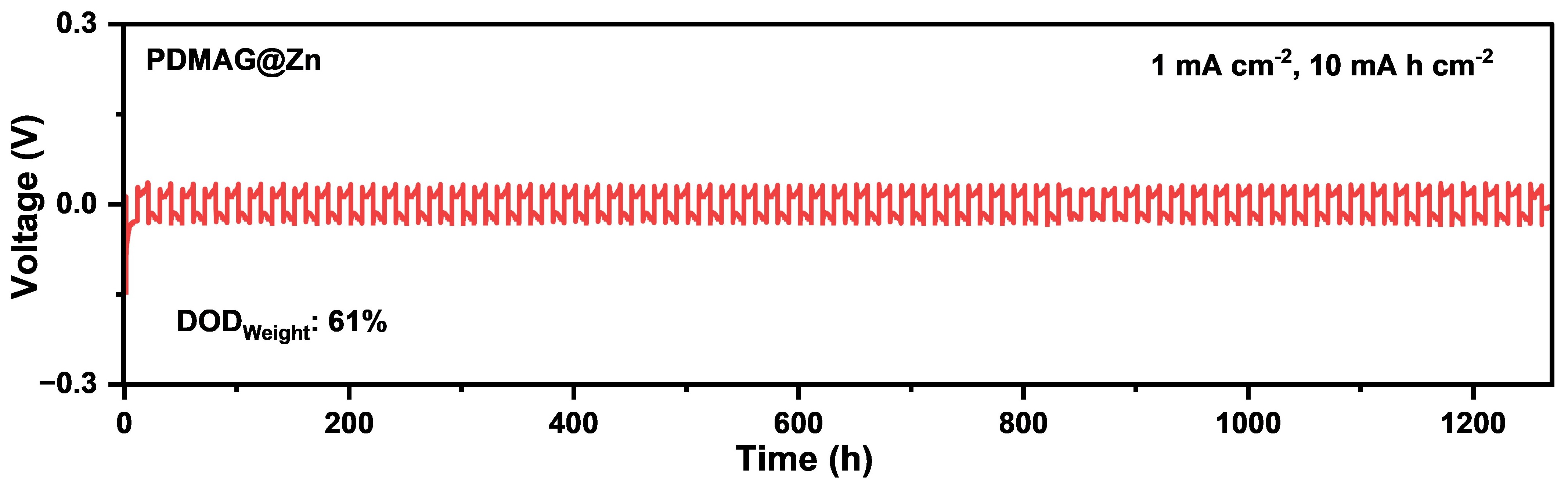


**Fig. S43** The performance of symmetrical PDMAG@Zn battery at 1 mA cm^-2^, 10 mA h cm^-2^


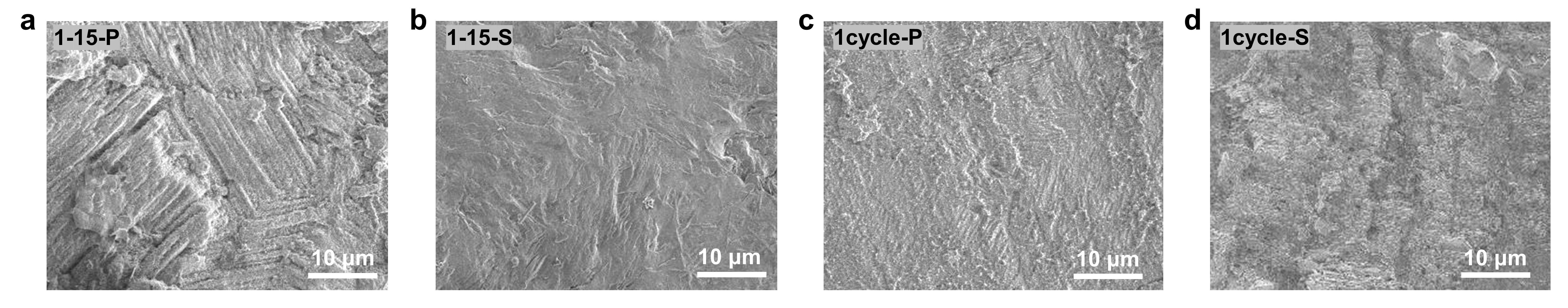


**Fig. S44** SEM images of plating and stripping sides for PDMAG@Zn surface after different cycles at 1 mA cm^-2^, 15 mA h cm^-2^


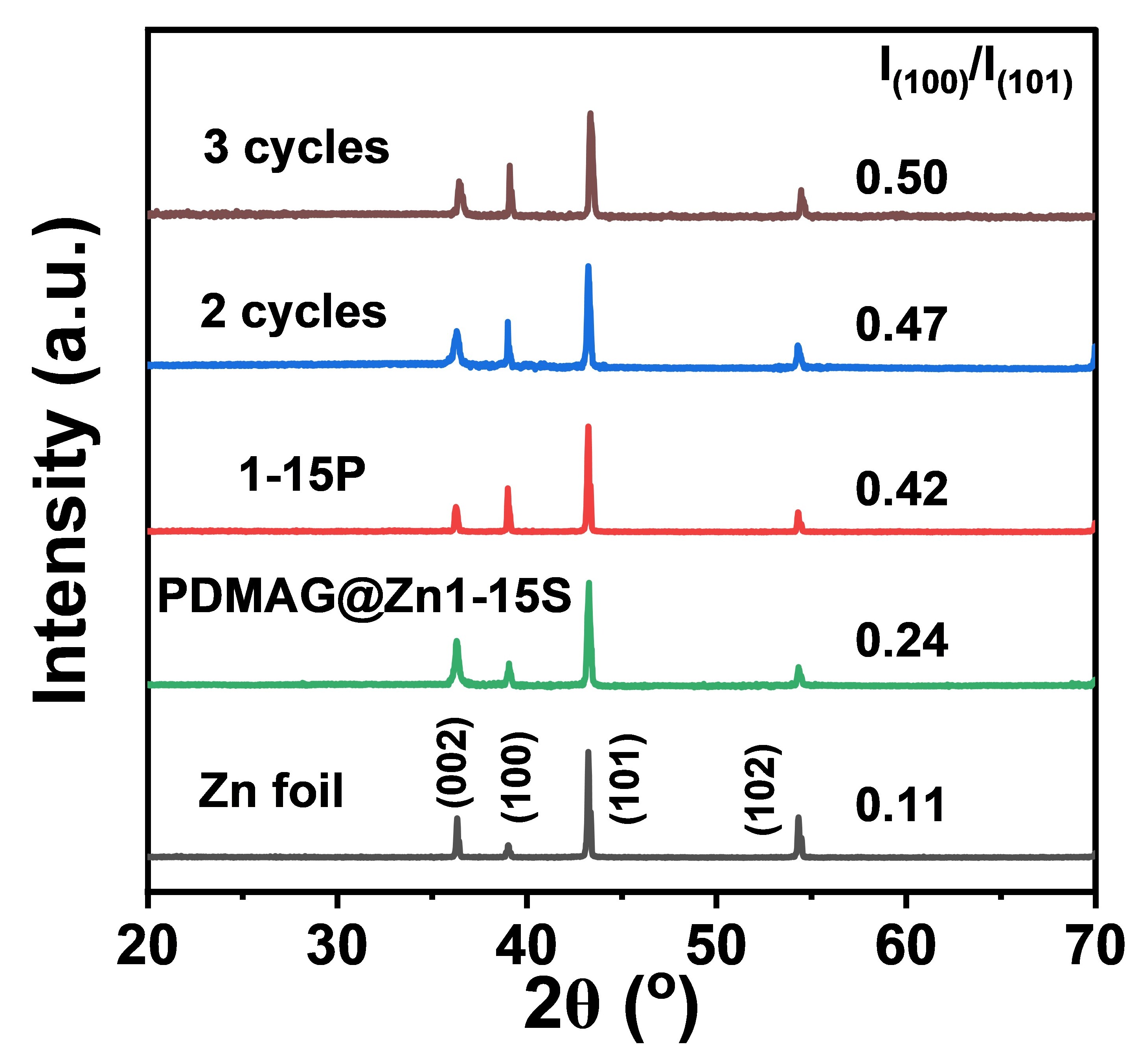


**Fig. S45** The related XRD spectra for PDMAG@Zn after different cycles at 1 mA cm^-2^, 15 mA h cm^-2^


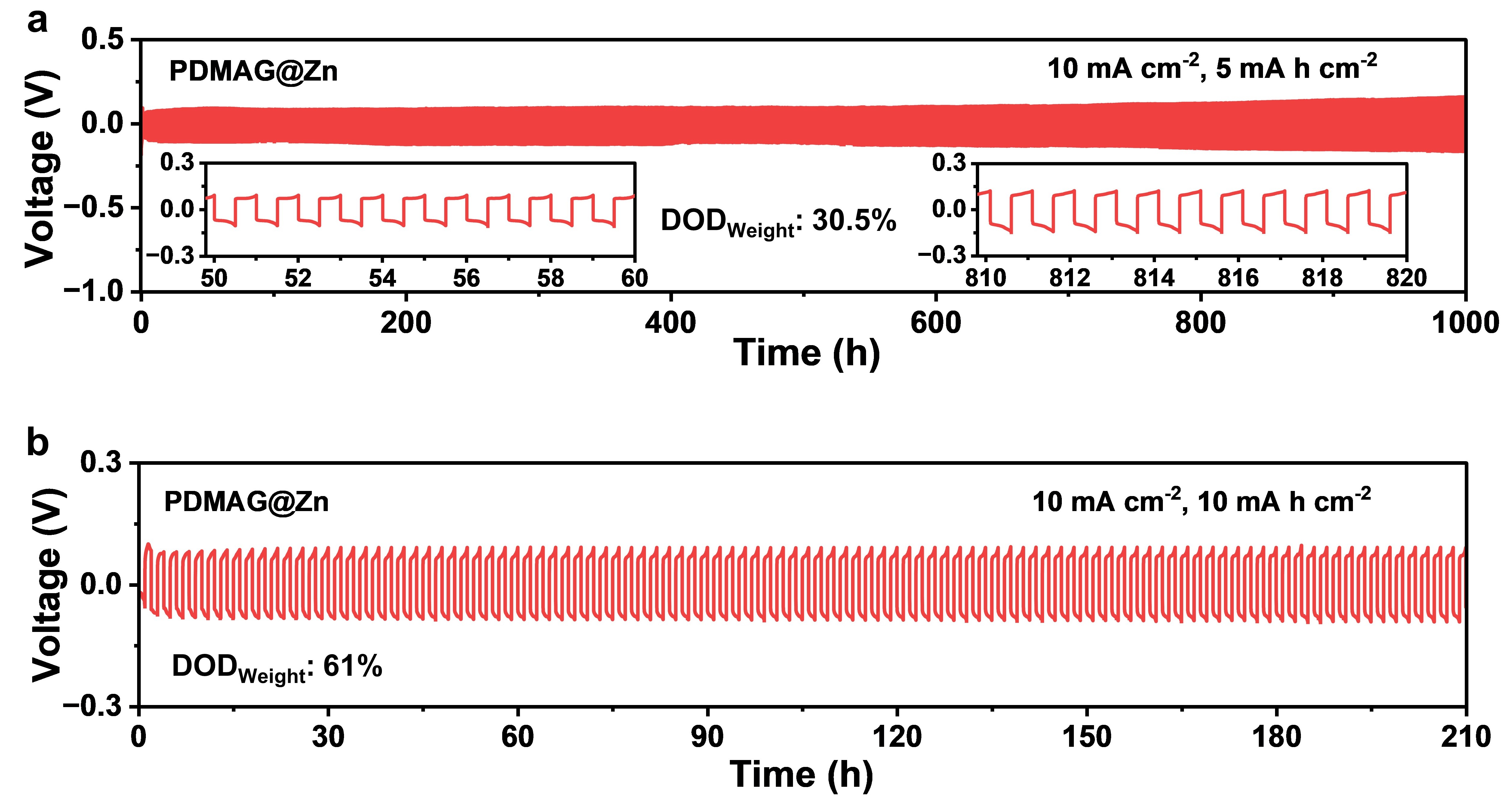


**Fig. S46** The performance of symmetrical Zn batteries with PDMAG@Zn at **a** 10 mA cm^-2^, 5 mA h cm^-2^ and **b** 10 mA cm^-2^, 10 mA h cm^-2^


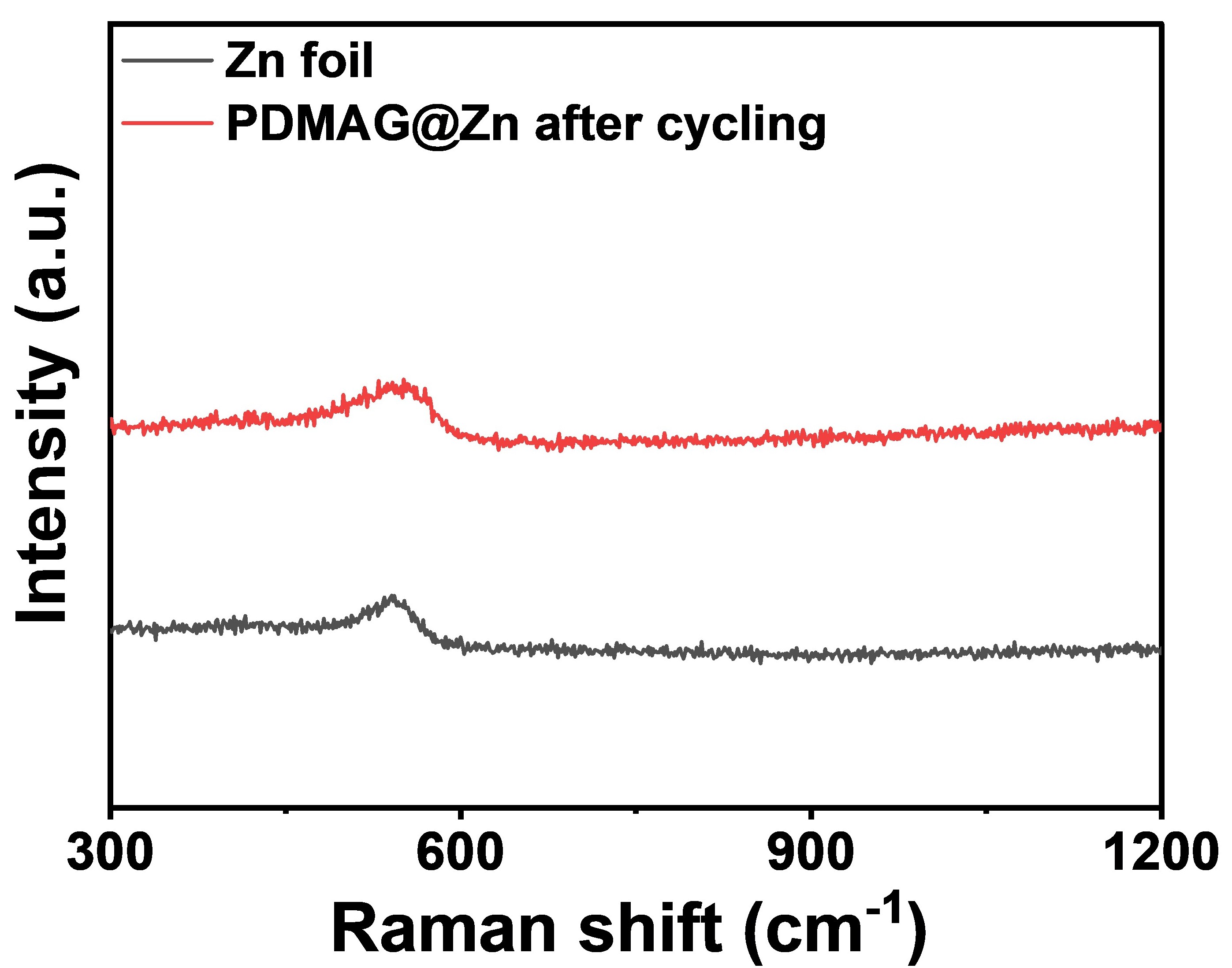


**Fig. S47** Raman spectra of Zn foil and PDMAG@Zn after 30 cycles at 10 mA cm^-2^, 10 mA h cm^-2^


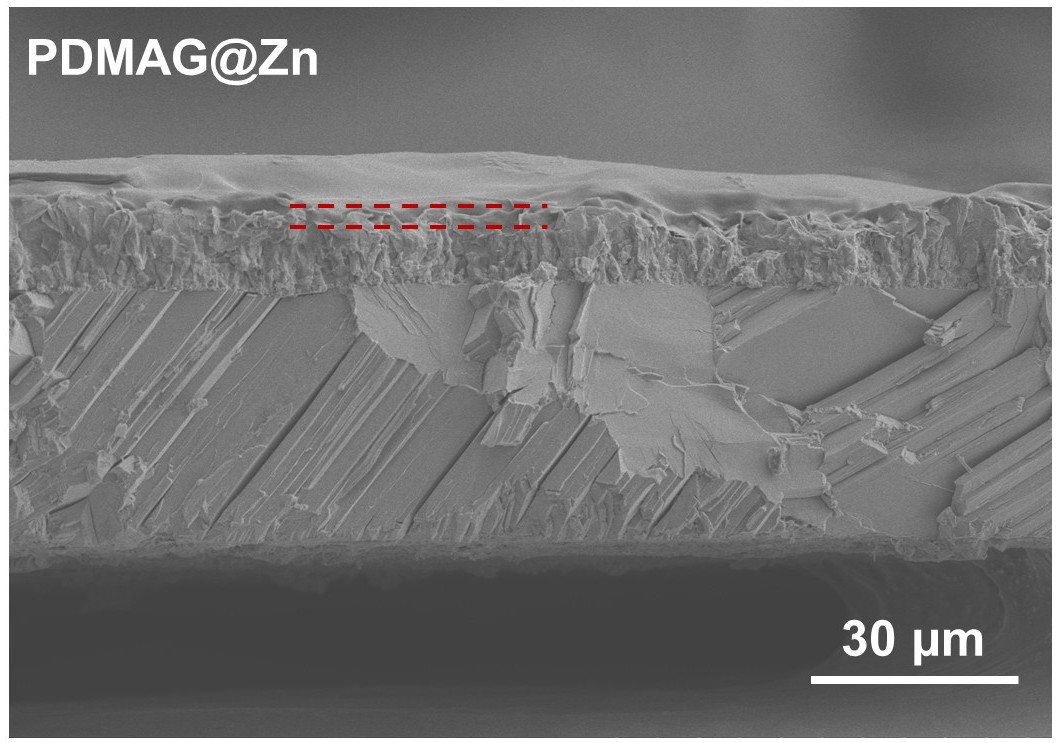


**Fig. S48** The cross-sectional SEM images of PDMAG@Zn after 30 cycles at 10 mA cm^-2^, 10 mA h cm^-2^


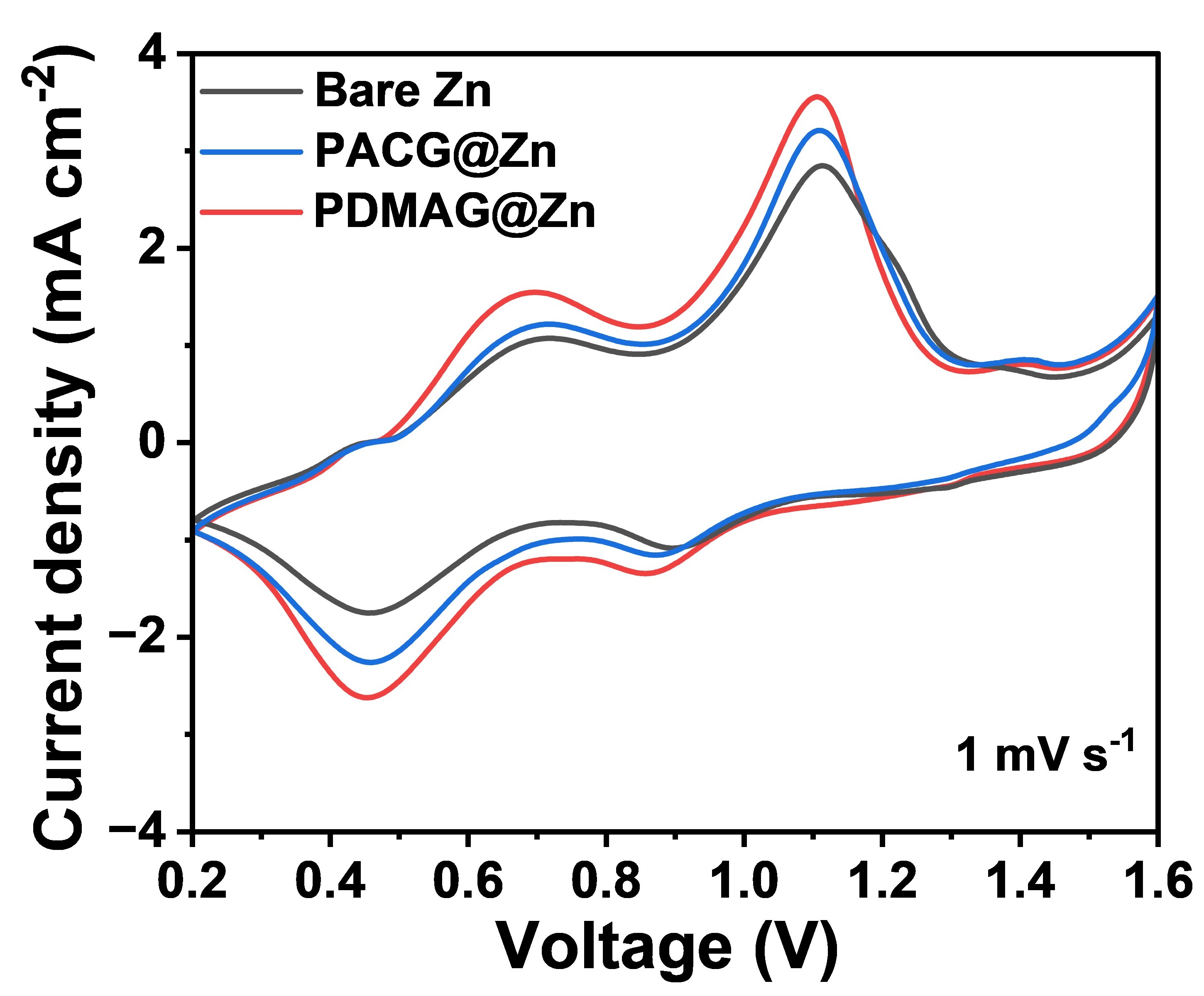


**Fig. S49** CV curves of Zn||V_2_O_5_ batteries with scan rate of 1 mV s^-1^


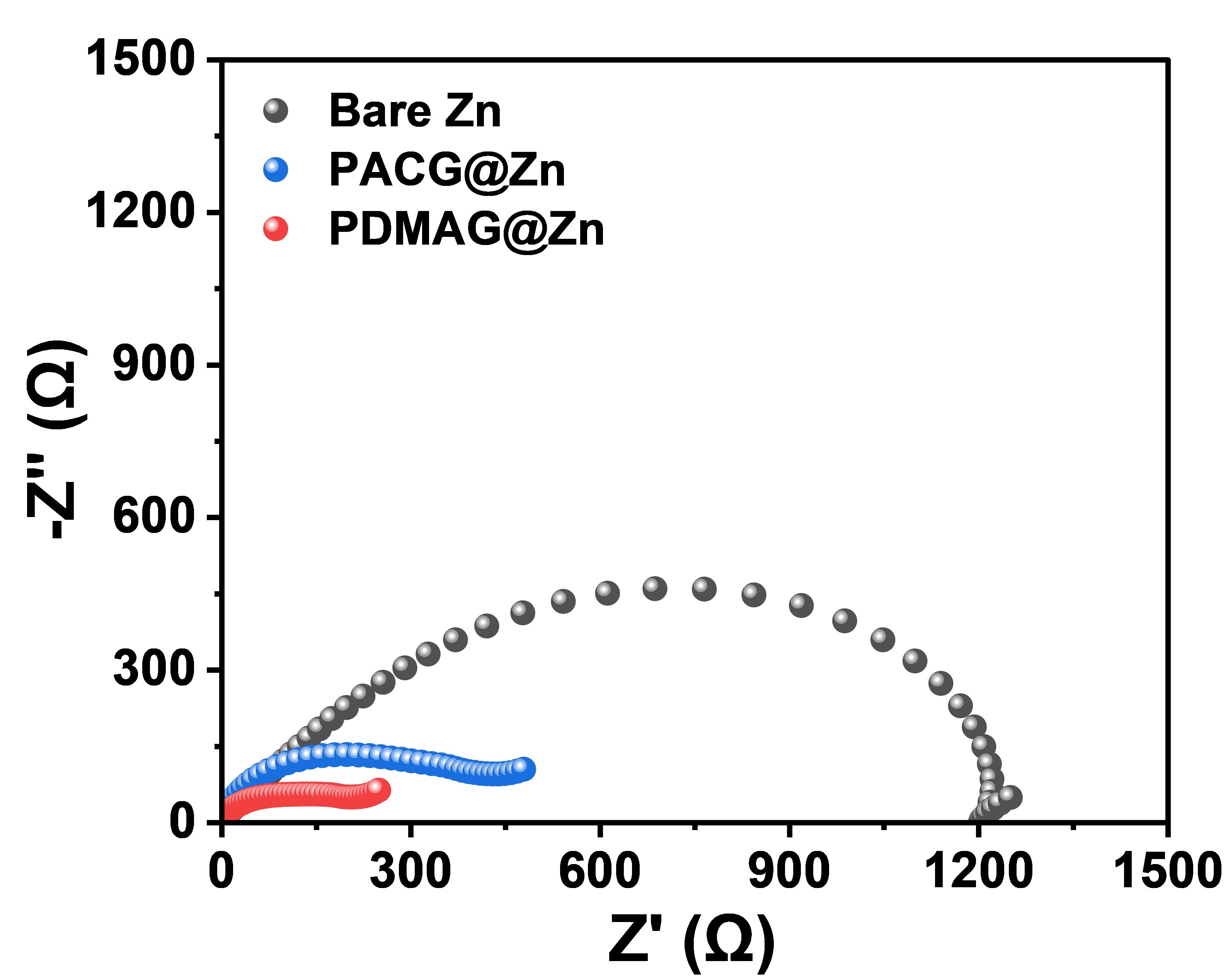


**Fig. S50** The EIS curves of Zn||V_2_O_5_ batteries


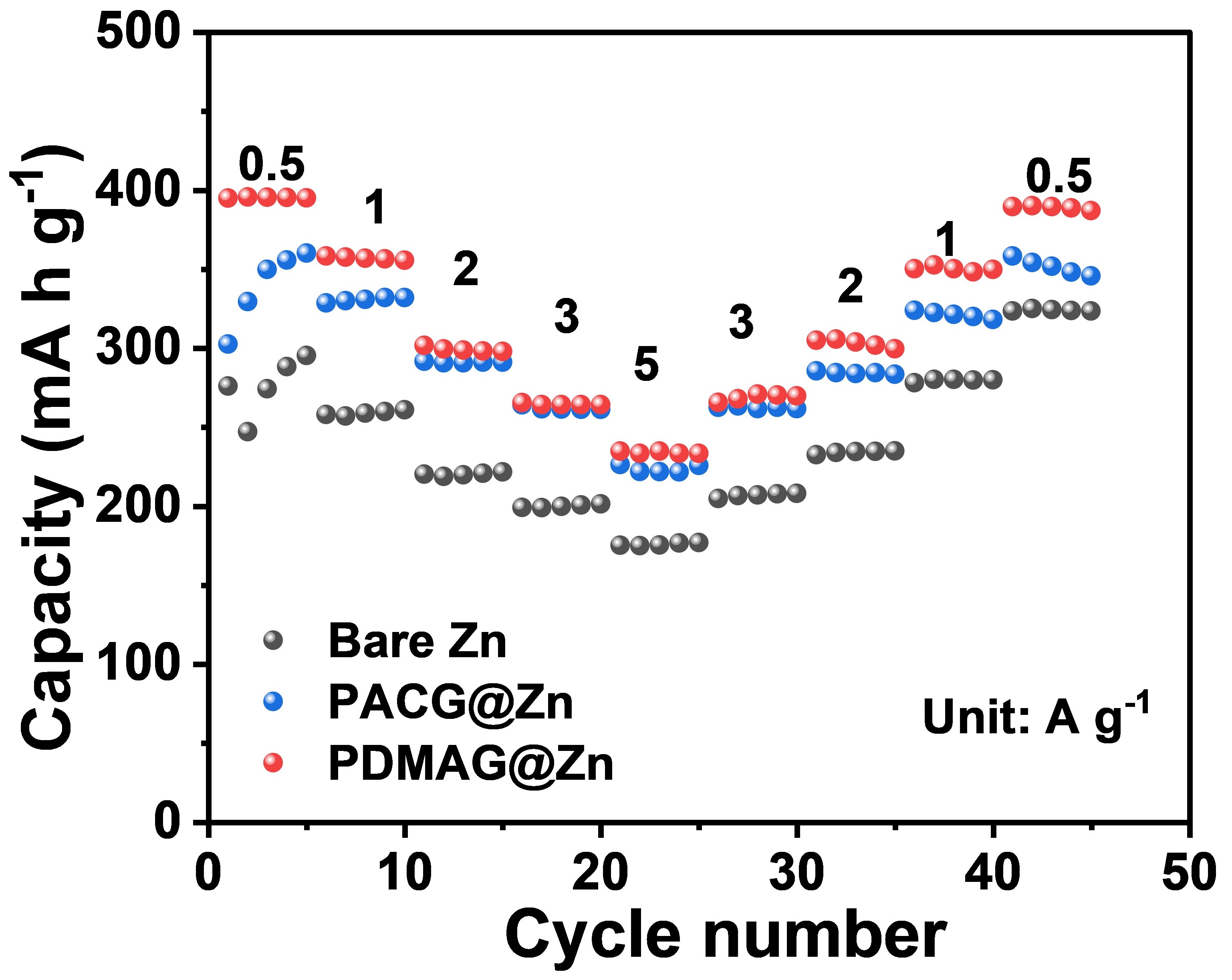


**Fig. S51** The rate performance of Zn||V_2_O_5_ batteries at different current densities


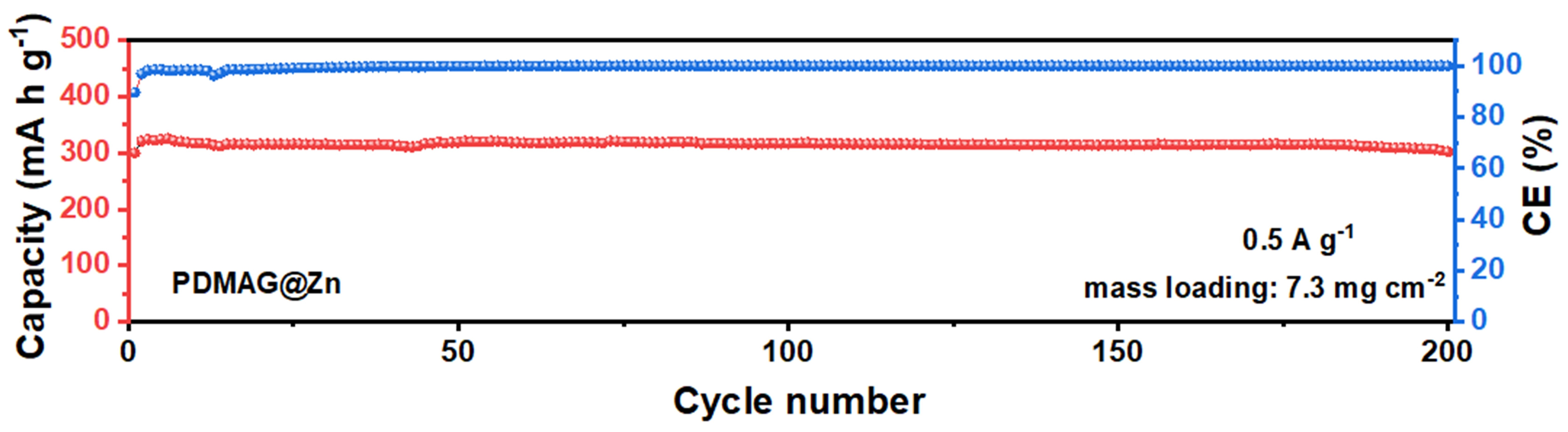


**Fig. S52** The performance for PDMAG@Zn||V_2_O_5_ battery with mass loading of 7.3 mg cm^-2^


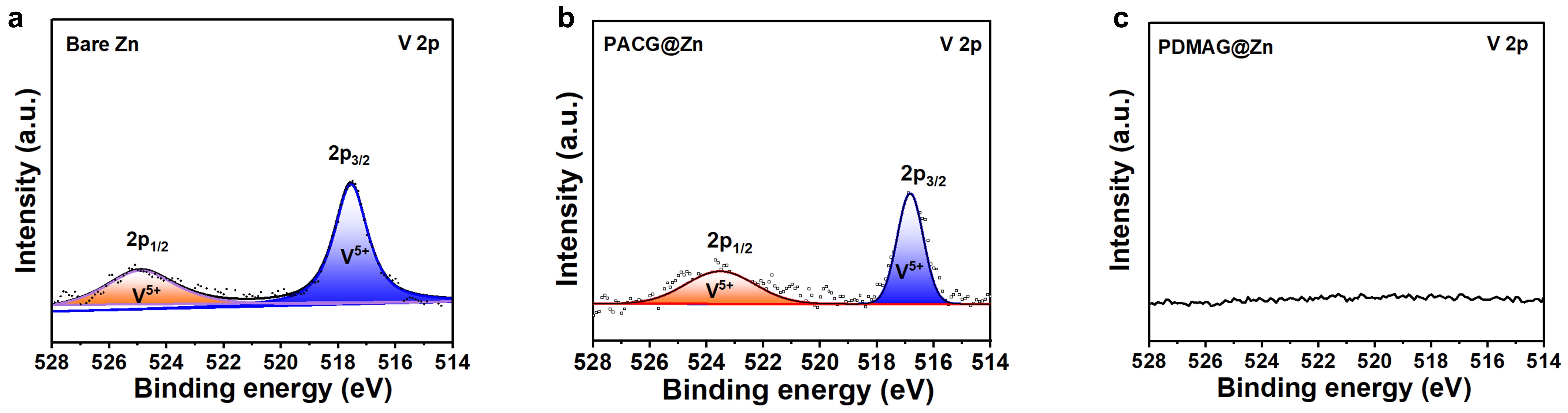


**Fig. S53** The XPS spectra of Zn||V_2_O_5_ batteries with **a** bare Zn, **b** PACG@Zn and **c** PDMAG@Zn after 100 cycles at 5 A g^-1^


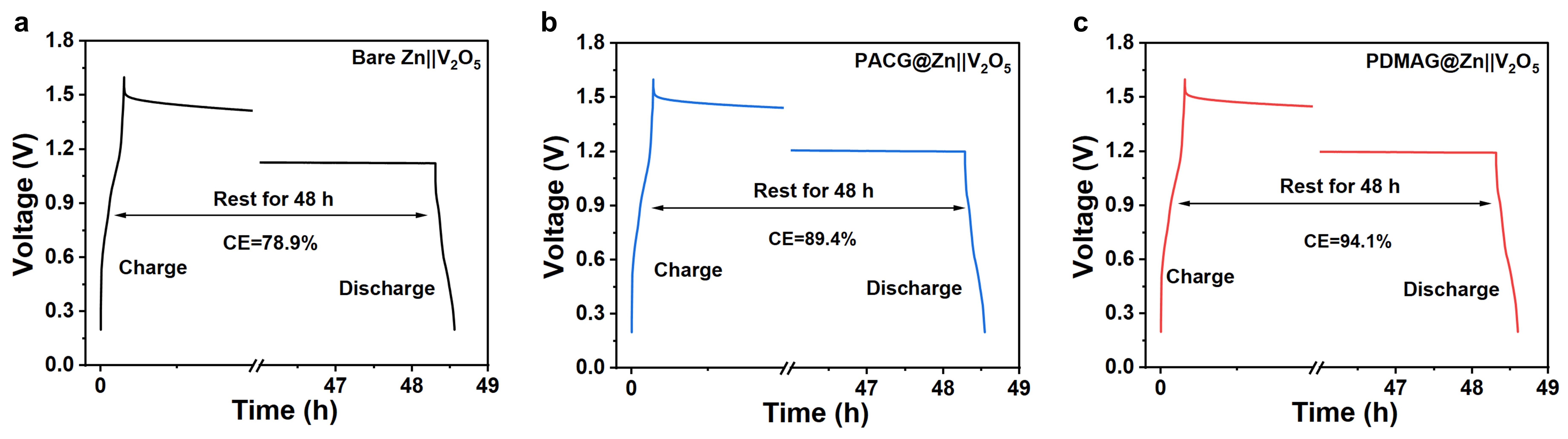


**Fig. S54** The self-discharge behavior of batteries with different Zn anodes


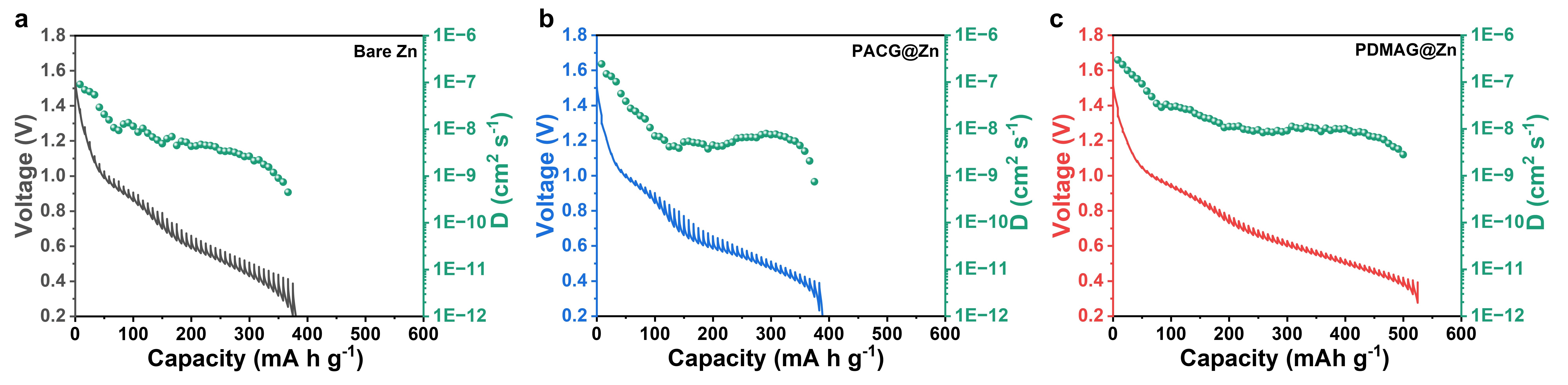


**Fig. S55** GITT tests of Zn||V_2_O_5_ batteries with different Zn anodes


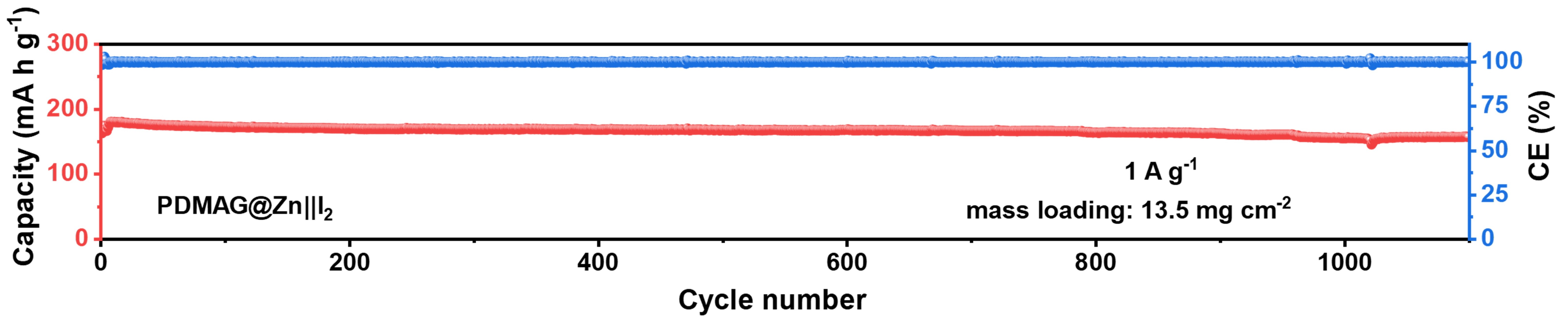


**Fig. S56** The cycling performance for PDMAG@Zn||I_2_ battery at 1 A g^-1^
